# Amaryllidaceae Alkaloids of Norbelladine-Type as Inspiration for Development of Highly Selective Butyrylcholinesterase Inhibitors: Synthesis, Biological Activity Evaluation, and Docking Studies

**DOI:** 10.3390/ijms22158308

**Published:** 2021-08-02

**Authors:** Abdullah Al Mamun, Filip Pidaný, Daniela Hulcová, Jana Maříková, Tomáš Kučera, Monika Schmidt, Maria Carmen Catapano, Martina Hrabinová, Daniel Jun, Lubica Múčková, Jiří Kuneš, Jiří Janoušek, Rudolf Andrýs, Lucie Nováková, Rozálie Peřinová, Negar Maafi, Ondřej Soukup, Jan Korábečný, Lucie Cahlíková

**Affiliations:** 1 ADINACO Research Group, Department of Pharmaceutical Botany, Faculty of Pharmacy, Charles University, Heyrovskeho 1203, 500 05 Hradec Kralove, Czech Republic; almamuna@faf.cuni.cz (A.A.M.); pidanyf@faf.cuni.cz (F.P.); hulcovd@faf.cuni.cz (D.H.); marikoj2@faf.cuni.cz (J.M.); perinovr@faf.cuni.cz (R.P.); negarm@faf.cuni.cz (N.M.); 2 Department of Pharmacognosy, Faculty of Pharmacy, Charles University, Heyrovskeho 1203, 500 05 Hradec Kralove, Czech Republic; janousj2@faf.cuni.cz; 3 Department of Bioorganic and Organic Chemistry, Faculty of Pharmacy, Charles University, Heyrovskeho 1203, 500 05 Hradec Kralove, Czech Republic; kunes@faf.cuni.cz; 4 Department of Toxicology and Military Pharmacy, Faculty of Military Health Sciences, University of Defence, Trebesska 1575, 500 01 Hradec Kralove, Czech Republic; kucera-t@email.cz (T.K.); martina.hrabinova@unob.cz (M.H.); daniel.jun@unob.cz (D.J.); lubica.muckova@unob.cz (L.M.); ondrej.soukup@fnhk.cz (O.S.); 5 Department of Chemistry, Faculty of Science, University of Hradec Králové, Rokitanského 62, 500 03 Hradec Králové, Czech Republic; monika.schmidt@uhk.cz (M.S.); rudolf.andrys@faf.cuni.cz (R.A.); 6 Department of Analytical Chemistry, Faculty of Pharmacy, Charles University, Heyrovskeho 1203, 500 05 Hradec Kralove, Czech Republic; catapanm@faf.cuni.cz (M.C.C.); novakoval@faf.cuni.cz (L.N.); 7 Biomedical Research Centre, University Hospital Hradec Kralove, Sokolska 581, 500 05 Hradec Kralove, Czech Republic

**Keywords:** Alzheimer’s disease, amaryllidaceae alkaloid, norbelladine-type, butyrylcholinesterase, docking studies

## Abstract

Alzheimer’s disease (AD) is a multifactorial neurodegenerative condition of the central nervous system (CNS) that is currently treated by cholinesterase inhibitors and the *N*-methyl-d-aspartate receptor antagonist, memantine. Emerging evidence strongly supports the relevance of targeting butyrylcholinesterase (BuChE) in the more advanced stages of AD. Within this study, we have generated a pilot series of compounds (**1**–**20**) structurally inspired from belladine-type Amaryllidaceae alkaloids, namely carltonine A and B, and evaluated their acetylcholinesterase (AChE) and BuChE inhibition properties. Some of the compounds exhibited intriguing inhibition activity for human BuChE (*h*BuChE), with a preference for BuChE over AChE. Seven compounds were found to possess a *h*BuChE inhibition profile, with IC_50_ values below 1 µM. The most potent one, compound **6**, showed nanomolar range activity with an IC_50_ value of 72 nM and an excellent selectivity pattern over AChE, reaching a selectivity index of almost 1400. Compound **6** was further studied by enzyme kinetics, along with in-silico techniques, to reveal the mode of inhibition. The prediction of CNS availability estimates that all the compounds in this survey can pass through the blood-brain barrier (BBB), as disclosed by the BBB score.

## 1. Introduction

Alzheimer’s disease (AD) is a devastating neurodegenerative age-dependent disorder characterized by memory loss and progressive cognitive impairment [1]. AD currently affects over 50 million people worldwide and is estimated to nearly triple by 2050 [2]. AD influences not only the quality of life of AD patients but also their families, with an enormous economic burden as well.

The etiology of AD has historically been ascribed to disrupted cholinergic neurotransmission [3], with more up-to-date explanations comprising excessive production of amyloid-β (Aβ) toxic species [4], dysfunction and accumulation of post-translationally modified tau protein [4], metal ions imbalance [5], and oxidative stress [6]. Although several clinical candidates have been generated upon these hypotheses over the past three decades of intensive research, current therapy of AD’s mild-to-moderate stage still relies almost exclusively on the administration of acetylcholinesterase (AChE; EC 3.1.1.7) inhibitors [7], represented by galantamine, donepezil, and rivastigmine [8]. Congruently, they halt the degradation of acetylcholine (ACh), thus elevating its levels at the synapses and facilitating the cholinergic receptors’ stimulation. It is noteworthy that in the later stages of the disease, the hydrolysis of ACh is more preferentially under the control of another cholinesterase enzyme, namely butyrylcholinesterase (also known as pseudocholinesterase; BuChE; EC 3.1.1.8) [9]. Notably, the crosstalk between the cholinergic system and other sub-pathologies of AD has been proven, pointing out the privileged role of cholinesterase inhibitors. For example, the cholinergic depletion promoted Aβ burden and tauopathy, both significantly contributing to cognitive decline [10]. Some studies have disclosed that the treatment with cholinesterase inhibitors can shift amyloid precursor protein (APP) processing to the non-amyloidogenic pathway [11], thus generating non-toxic Aβ species. Professor Inestrosa’s group first reported the association between AChE and assembly of Aβ [12]. This study constitutes the basis for intensive research in the field of so-called dual binding site AChE inhibitors that can slow down the assembly of Aβ via the interaction with the peripheral anionic site (PAS) of the enzyme [12]. BuChE has also been suggested to participate in the formation and maturation of Aβ plaques [13,14]. Moreover, some studies have shown that BuChE may contribute to the pathogenesis of type 2 diabetes mellitus (T2DM) by causing insulin resistance; the latter is considered as one of the major risk factors contributing to AD onset [15,16]. Recently, several new structural scaffolds have been designed, synthesized, and studied as new cholinesterase inhibitors [17,18,19].

Natural products (NPs) represent one of the major sources for the development of novel drugs for a variety of disorders, including neurodegenerative ones [20,21]. Drug development starting from NPs can be considered a highly challenging task given the NPs structural complexity, a number of chiral centers, laborious multi-step syntheses, and unfavorable ADMET properties [22]. Only some of the NPs are suitable bioactive pharmacophores to be directly used as clinical drugs. Indeed, after deciphering structure-activity relationships (SAR), NPs can be considered as lead compounds requiring further optimization like potency/selectivity/water solubility improvement, metabolic stability enhancement, and reducing side effects [23]. From the large family of NPs, a significant number of them have been identified as AChE/BuChE inhibitors [24,25,26,27]. With respect to neurodegenerative disorders, only galantamine, the Amaryllidaceae alkaloid isolated from the bulbs of snowdrops, e.g., *Galanthus woronowii*, is currently used in the management of AD symptoms.

**Figure 1:**
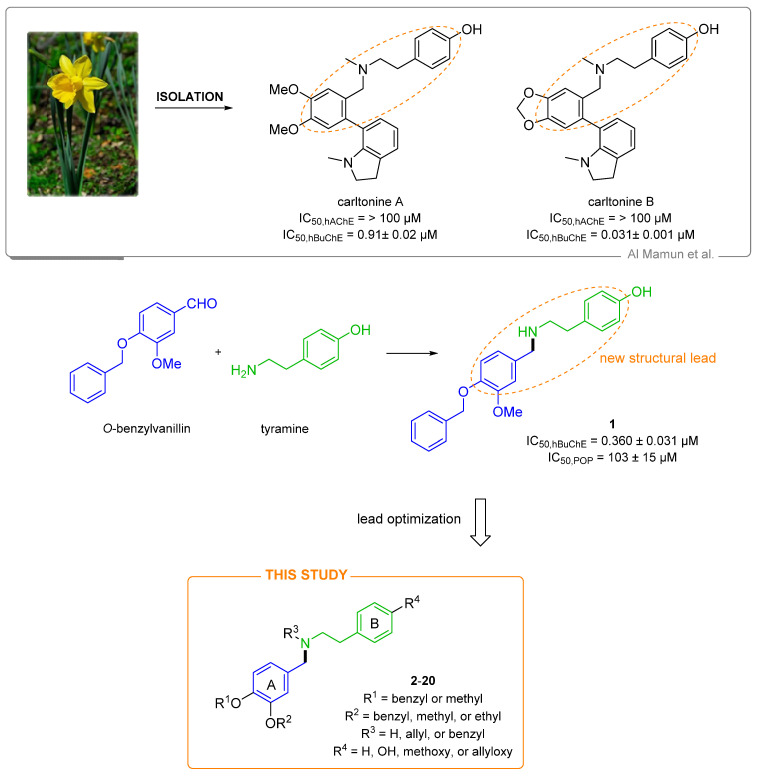
Design of novel cholinesterase inhibitors derived from hit compound **1** and norbelladine-type Amaryllidaceae alkaloids isolated from *Narcissus pseudonarcissus* cv. Carlton, namely carltonine A and B.

In the present study, we report the synthesis of a pilot series (compounds **1**–**20**; Figure 1) of highly selective *h*BuChE inhibitors. Their structure has been inspired by Amaryllidaceae alkaloids (AAs) of the belladine-type, namely carltonine A and B (Figure 1, [28]). We explored the SAR by establishing their AChE/BuChE inhibition properties. We have also determined the prolyloligopeptidase (POP; EC 3.4.21.2) inhibition ability of this class of compounds since its blockade is associated with cognitive improvement. The inhibition of POP mediates the signaling cascade by releasing Ca^2+^ at the endoplasmatic reticulum membrane, which may induce memory enhancement [29]. Some highly selective POP inhibitors entered clinical trial testing for neurodegenerative disorders, but these were terminated, mostly due to their shortcomings related to the compound’s pharmacokinetic profile. Besides, we have also predicted the CNS availability of the selected compounds. Molecular dynamic (MD) simulation was performed for the most active BuChE inhibitor (**6**) to reveal the structural requirements responsible for the ligand binding. Complex **6**-BuChE was compared with BuChE inactive inhibitor and topological isomer **5**.

## 2. Design

AAs are an extensively studied class of NPs. As mentioned previously, one such drug from this family, namely galantamine, was approved for AD treatment in 2001 [8]. Lycorine, haemanthamine, pancratistatine, and narciclasine are known to possess antiproliferative properties [30,31,32]. Montanine governs antidepressive, anticonvulsive, anti-rheumatic, antimicrobial, and antitumor activities [33]. The emerging data and potential applicability of AAs spur the search for new alkaloids from this family.

Indeed, within our previous study, we isolated new alkaloids from *Narcissus pseudonarcissus* cv. Carlton, namely carltonine A and B (Figure 1), demonstrating highly selective in vitro *h*BuChE inhibition potency in the nanomolar range [28] (Figure 1). Unfortunately, these alkaloids are present in plant material only in trace amounts, and they cannot be isolated for either more detailed biological investigation or commercial use. Thus, we decided to preserve some of the crucial structural requirements from carltonine A/B that are plausibly responsible for high BuChE inhibition activity, i.e., the 4-[2-(benzylamino)ethyl]phenol moiety, and modified other molecular regions to elucidate SAR. Specifically, we were interested in (i) the role of the secondary or tertiary amino group (the presence of allyl group), (ii) etherification of the phenolic hydroxyl group at aromatic ring B, and (iii) position of alkoxy or aryloxy substituents at benzene ring A (Figure 1), all concerning cholinesterase inhibitory activity. Some structural aspects related to the design of the novel family of compounds investigated herein can be found in the previously reported studies on disease-modifying multifunctional anti-AD agents. Indeed, one such family of compounds carries a benzylamine moiety, the critical fragment for cholinesterase binding [34]. It has also been observed that various aromatic substitutions in the benzylamine region, along with the introduction of a basic moiety into alicyclic amines, can yield cholinesterase inhibitors endowed with additional activity to beta-secretase 1 (BACE-1) [35,36]. Taking the precedents mentioned above, in this study, we have designed and synthesized 20 novel potential cholinesterase inhibitors derived either from commercially available *O*-benzylvanillin, or *O*-benzylisovanillin tethered to tyramine. Further details regarding the structural modifications are outlined in Figure 1.

## 3. Results and Discussion

### 3.1. Synthesis of Novel hBuChE Inhibitors from O-benzylvanillin, O-benzylisovanillin, and 3-ethoxy-4-methoxybenzaldehyde (***1**–**20***)

**Scheme 1:**
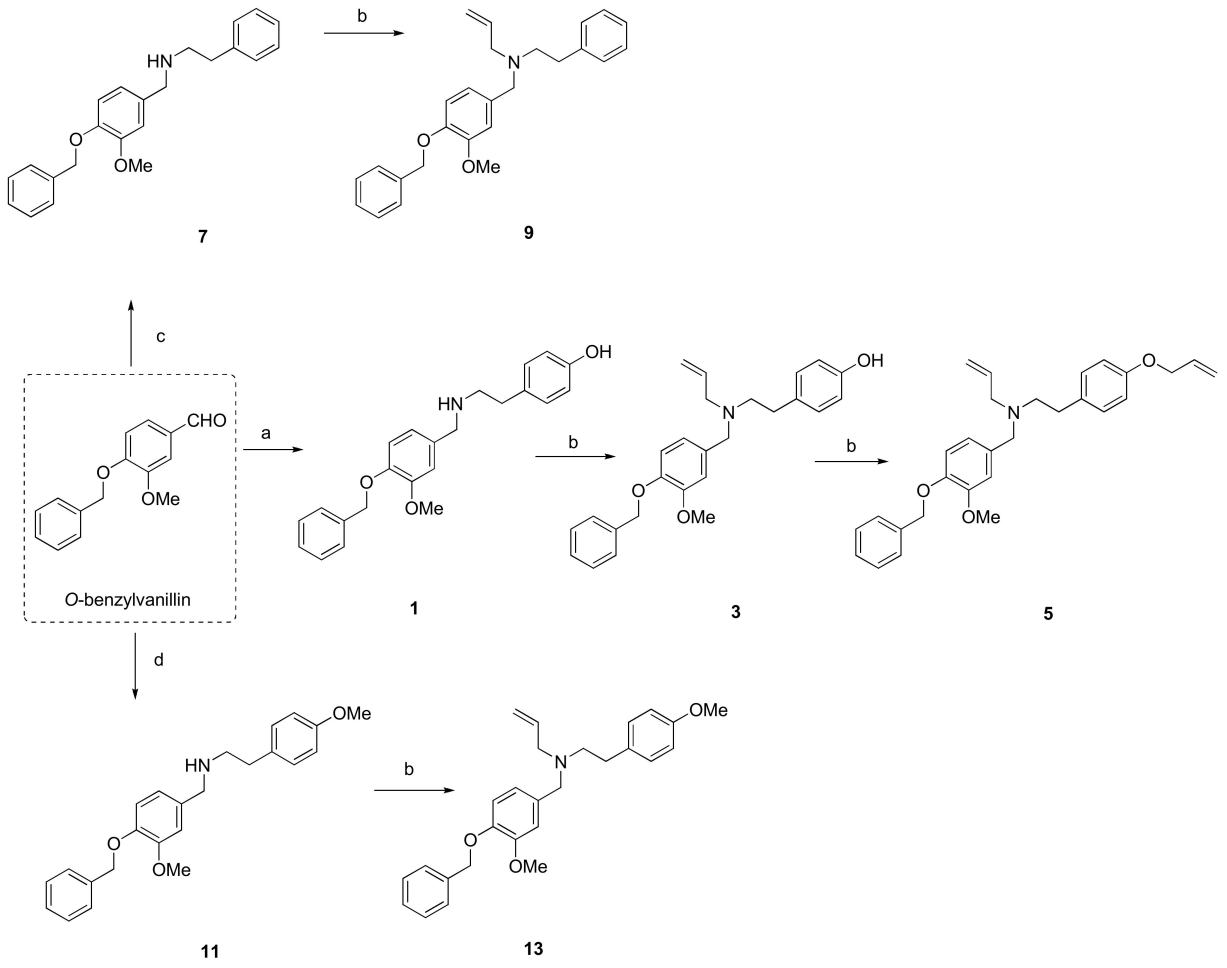
Synthesis of novel *h*BuChE inhibitors **1**, **3**, **5**, **7**, **9**, **11** and **13** starting from *O*-benzylvanillin. Reagents and conditions: (**a**): (1) tyramine, MeOH; (2) NaBH_4_, rt, 3 h; (**b**): allyl bromide (1.3 eq.), NaH (1.2 eq.), THF; (**c**): (1) 2-phenylethan-1-amine, MeOH; (2) NaBH_4_, rt, 3 h; (**d**): (1) 2-(4-methoxyphenyl)ethan-1-amine, MeOH; (2) NaBH_4_, rt, 3 h.

**Scheme 2:**
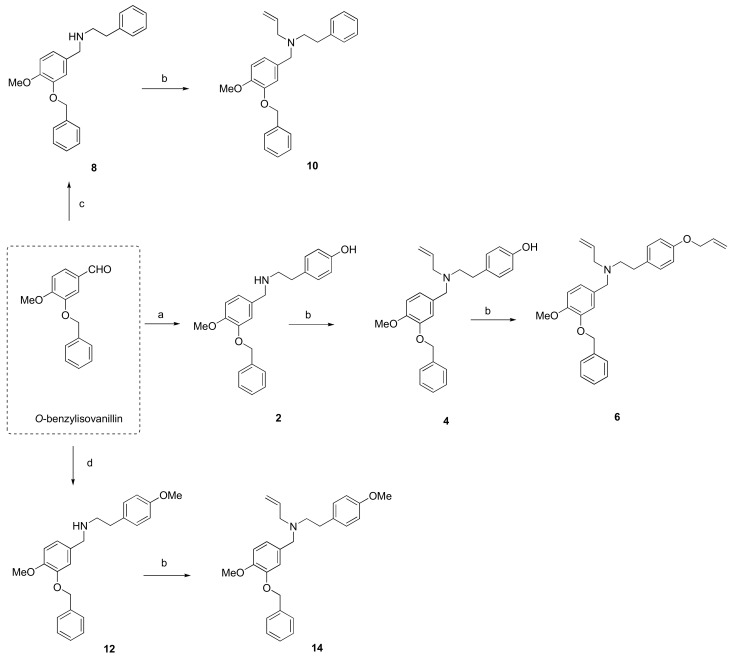
Synthesis of novel *h*BuChE inhibitors **2**, **4**, **6**, **8**, **10**, **12** and **14** starting from *O-*benzylisovanillin. Reagents and conditions: (**a**) (1) tyramine, MeOH; (2) NaBH_4_, rt, 3 h; (**b**) allyl bromide (1.3 eq.), NaH (1.2 eq.), THF; (**c**) (1) 2-phenylethan-1-amine, MeOH; (2) NaBH_4_, rt, 3 h; (**d**) (1) 2-(4-methoxyphenyl)ethan-1-amine, MeOH; (2) NaBH_4_, rt, 3 h.

**Scheme 3:**
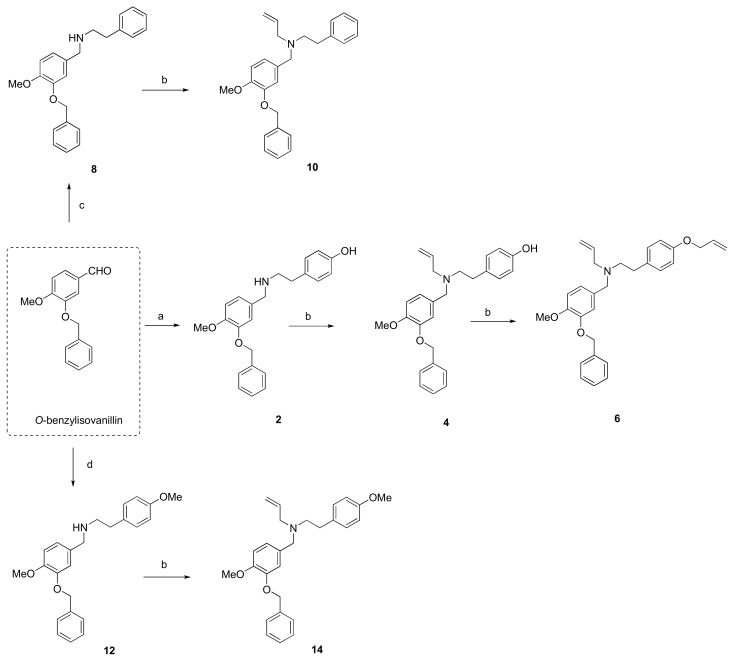
Synthesis of novel *h*BuChE inhibitors **15**–**20** starting from 3-ethoxy-4-methoxybenzaldehyde. Reagents and conditions: (**a**) (1) tyramine, MeOH; (2) NaBH_4_, rt, 3 h; (**b**) allyl bromide (1.3 eq.), NaH (1.2 eq.), THF; (**c**) (1) 2-phenylethan-1-amine, MeOH; (2) NaBH_4_, rt, 3 h; (**d**) benzyl bromide (1.3 eq), NaH (1.2 eq.), THF.

The hit compound **1** was synthesized from commercially available *O*-benzylvanillin and tyramine in excellent yield (95%), according to the published procedure used in the total synthesis of siculine (Scheme 1) [37]. Different structural modifications were explored using condensation of *O*-benzylvanillin (Scheme 1), *O*-benzylisovanillin (Scheme 2), and 3-ethoxy-4-methoxybenzaldehyde (Scheme 3) with primary amines like tyramine, 2-phenylethan-1-amine, and 2-(4-methoxyphenyl)ethan-1-amine, to elucidate the structure-activity relationship of the synthesized compounds. The conditions of the reductive amination furnished desired secondary amines **2**, **7**, **8**, **11**, **12**, **15**, and **18** in good yields. Subsequent reaction of the prepared compounds with an excess of allyl bromide afforded the corresponding *N*-allyl derivatives **3**, **4**, **9**, **10**, **13**, **14**, **16**, and **19**, respectively. In addition to this allylation, compound **18** also underwent another alkylation to give *N*-benzyl derivative **20** (Scheme 3). Further nucleophilic substitution with additional allyl bromide was performed at the phenolic group of compounds **3**, **4**, and **16** to obtain allyl ethers **5**, **6**, and **17**.

In general, a portfolio of 20 compounds with a norbelladine framework was constructed. All prepared derivatives were characterized by LC-MS, HRMS, and NMR analysis. The purity was determined higher than 96% for each synthesized compound (Supplementary Materials).

### 3.2. In Vitro Cholinesterase Inhibitory Activities of New Norbelladine Derivatives (***1**–**20***)

All compounds (**1**–**20**) were initially screened for *h*AChE/*h*BuChE inhibition potency at a concentration of 100 µM according to a slightly modified Ellman’s method [28,38]. Compounds displaying inhibition ability >60% against one or both cholinesterases at the screening concentration were selected for the determination of their IC_50_ values (Table 1). Galantamine and eserine were used as positive controls in the *h*AChE/*h*BuChE assay. The results are summarized in Table 1. All the compounds displayed only weak to moderate *h*AChE inhibition potency (IC_50_ > 20 µM) and were selective towards *h*BuChE. Indeed, all the novel compounds revealed *h*BuChE inhibition potency in the micromolar to low nanomolar range (Table 1). Compound **5** was the only one displaying no inhibition potency against either enzyme.

Regarding *h*AChE inhibition, only two derivatives, namely **10** and **14**, exerted moderate inhibition potency with IC_50_ values of 21.51 ± 0.62 µM, and 37.65 ± 1.68 µM, respectively. The rest of the family demonstrated only weak or no *h*AChE inhibition potency at the screened concentration of 100 µM (Table 1). On the contrary, except for compound **5**, all derivatives showed intriguing inhibition potency against *h*BuChE at the screening concentration; thus, we were able to determine their IC_50_ values to draw the structure-activity relationship. Compound **6**, originating from *O*-benzylisovanillin and tyramine with two allyl substitutions, was the most pronounced *h*BuChE inhibitor (IC_50_ = 72 ± 5 nM). Most strikingly, **6** emerged as the most selective *h*BuChE with a SI value of almost 1400. Surprisingly, its close topological derivative **5** was completely inactive in the *h*BuChE assay (IC_50_ > 100 µM). In this specific case, the complete loss of inhibition ability is associated with the opposite positioning of the methoxy- and benzyloxy-groups on the aromatic ring (further inspected by in silico techniques). The etherification/amination by allyl of **1** gradually decreases the inhibition ability of compounds **3** and **5**, with compound **5**, endowed with two allyl appendages, being completely inactive. When template compound **2** was used, the opposite effect was observed, showing that the double-allyl derivative **6** was one order of magnitude more active than compound **4** (Table 1). Structure modifications in position C-4’ of ring B by OH, H, and OMe in compounds **1**, **7**, and **11**, respectively, were associated with gradual loss of *h*BuChE inhibition potency. This is the case when either the hydroxy group in **1** is removed, like in **7**, or replaced by a methoxy group, like in **11** (IC_50_ = 0.36 ± 0.03 µM, 1.28 ± 0.05 µM, and 2.39 ± 0.27 µM, for **1**, **7**, and **11**, respectively). A similar effect was observed in the series derived from *O*-benzylisovanillin (compounds **2**, **8**, and **12** with IC_50_ values of IC_50_ = 0.29 ± 0.02 µM, 1.10 ± 0.05 µM, and 1.12 ± 0.11 µM, respectively). To elucidate further the necessity of benzyl attachment, a small series stemmed from 3-ethoxy-4-methoxybenzaldehyde (**15**–**20**; Scheme 3) was built. Within this subset, **20** (IC_50_ = 0.69 ± 0.03 µM) was classified as the top-ranked *h*BuChE inhibitor, possessing *N*-benzyl substitution at the central nitrogen atom. This pattern of behavior might be ascribed to better occupancy of the ligand in the bulky gorge of *h*BuChE [39].

Recently, close derivatives with a norbelladine framework (*N*-benzyl-2-phenylethan-1-amine congeners) have been developed by Carmona–Viglianco et al. [40] and screened for their AChE/BuChE potency. The compounds reported herein include the same fundamental unit as compound **1**, but they possess different substitution patterns within the A-ring. Generally, these derivatives yielded moderate to weak inhibition potency against both cholinesterases with IC_50_ values above 10 µM. Most importantly, only one compound displayed selectivity to BuChE higher than 10.

### 3.3. POP Inhibition Activity of Selected Norbelladine Derivatives

Recently, some POP inhibitors have been evaluated in clinical trials to reverse and/or attenuate memory loss. Inhibition of POP can represent an additional supporting approach in AD treatment; thus, the POP inhibition potential of the most active norbelladine derivatives (**1**–**4**, **6** and **14**) was tested. Unfortunately, the low solubility of tested compounds in buffer allowed the determination of IC_50_ only for compound **1**. This compound showed slightly lower POP inhibition potency (IC_50_ = 186 ± 14 µM) compared to used standard POP inhibitor berberine (IC_50_ = 142 ± 21 µM) [41].

### 3.4. Prediction of CNS Availability

Significant brain exposure is a critical factor in the design of novel drugs to confront AD. Pursuant to this prerequisite, we have applied in-silico calculation of the so-called blood-brain barrier (BBB) score [42]. It is a well-validated predictive tool recently developed to distinguish between CNS and non-CNS drugs. It builds upon several physicochemical properties, including molecular weight, topological polar surface area, pKa, number of aromatic rings, heavy atoms, number of hydrogen bond donors, and acceptors. Most importantly, the BBB score exceeded the reliability of other predictive algorithms with a high value of area under the receiver operating characteristic curves (AUC), reaching a value of 0.86. All the compounds with BBB-score values higher than 4.0 are postulated to enter the CNS area. Indeed, all the novel compounds in this study (**1**–**20**) reached a BBB-score ranging between 4.53 to 5.60 (Table 1), showing their ability to permeate the BBB. It is noteworthy that the template drugs carltonine A (BBB score 4.52) and carltonine B (4.54) displayed comparable values to compound **1**, whereas the rest of the family revealed higher BBB score values.

### 3.5. Enzyme Kinetic Analysis of Compound ***6***

**Figure 2:**
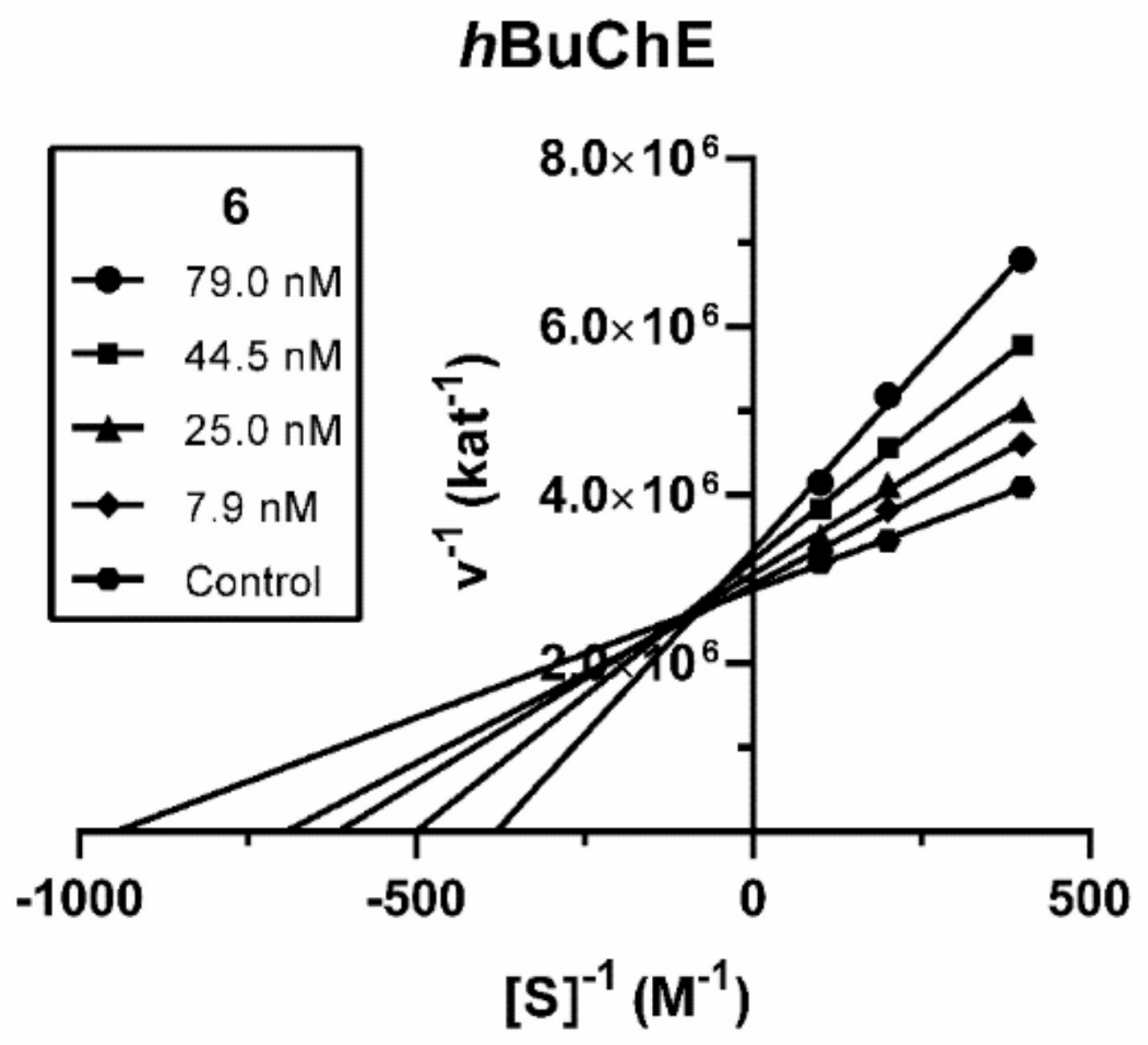
Steady-state mixed-type inhibition of *h*BuChE substrate hydrolysis by compound **6** at different concentrations. Lineweaver−Burk plots of initial velocity at increasing substrate concentrations (2.5–10.0 mM) are presented. Lines were derived from a linear regression of the data points.

Compound **6**, the derivative with the most robust inhibitory activity, was subjected to enzyme kinetics analysis to determine the mode of *h*BuChE inhibition. Inhibition kinetics were elucidated from velocity curves that were measured at several concentrations of substrate butyrylthiocholine and test compound. The type of enzyme inhibition and corresponding kinetic parameters (K*_i_* and *K*_i′_) were determined by nonlinear regression analysis. Results for each type of model of inhibition (competitive, non-competitive, uncompetitive, and mixed) were compared by the sum-of-squares *F*-test. Statistical analysis showed a mixed type of inhibition (*p* < 0.05), which is in line with the Lineweaver–Burk plot, used for visualization of the obtained data (Figure 2).

The intersection of lines is located above the x-axis, which means a reversible binding mode to both free enzyme and enzyme-substrate complex, with a stronger affinity to the free enzyme (*K*_i_ < *K*_i′_). *K*_m_ was increased whereas *V*_max_ was slightly reduced at a higher concentration of **6**. A *K*_i_ value of 60.3 ± 11.6 nM and *K*_i′_ of 347 ± 79 nM were measured against *h*BuChE.

### 3.6. Molecular Dynamic Simulation for Compounds ***5*** and ***6***

**Figure 3:**
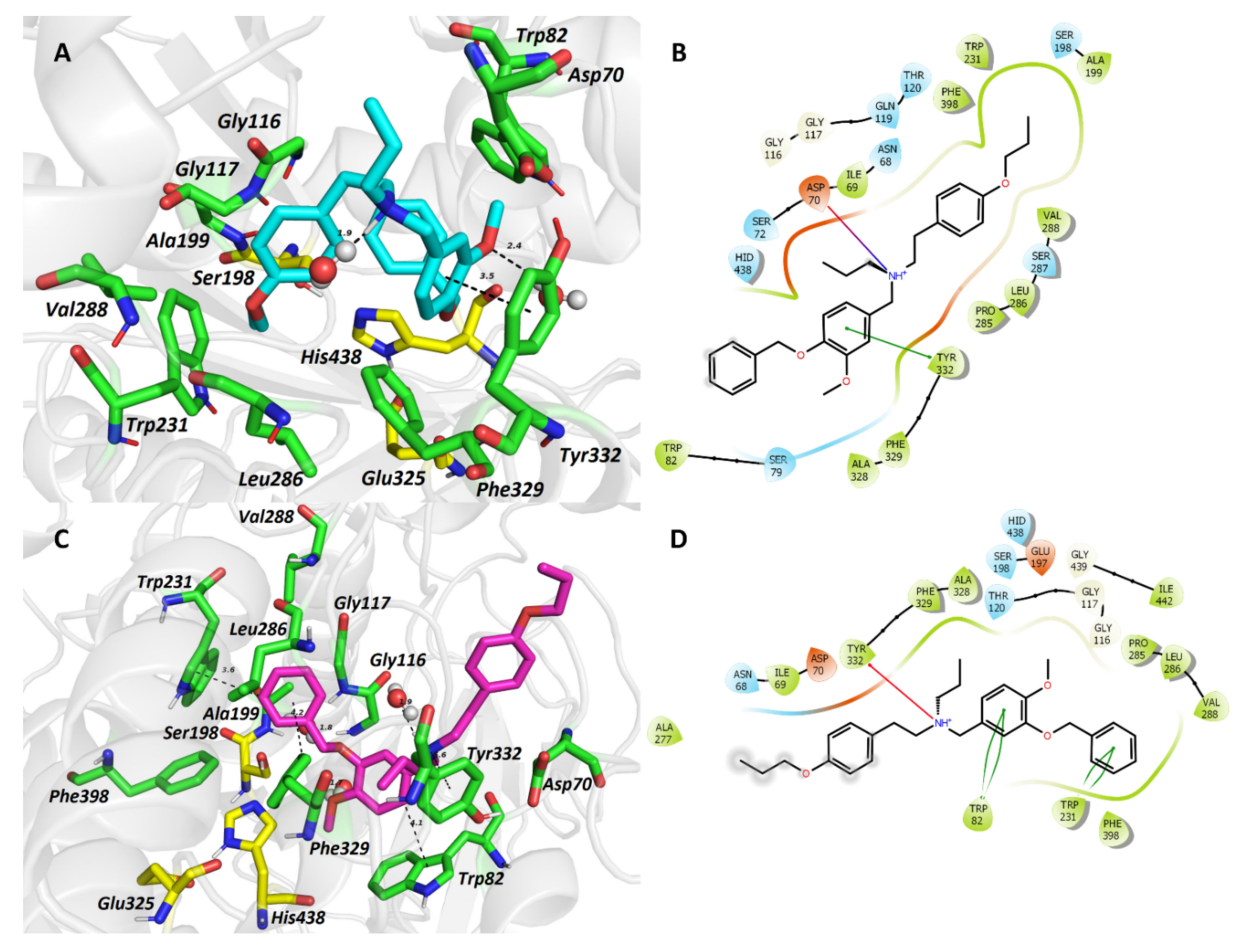
The top-scored docking poses of ligands **5** (**A**,**B**) and **6** (**C**,**D**) in the *h*BuChE active site (PDB ID: 6QAA) [43] from MD simulation. The ligands are displayed in light blue (**5**, **A**) and purple (**6**, **C**); important amino acid residues responsible for ligand anchoring are shown in green. Catalytic triad residues are displayed in yellow. Important interactions are rendered by black dashed lines; distances are measured in Ångstroms (Å). The rest of the receptor is displayed in light-grey cartoon conformation (**A**,**C**). Figures (**A**,**C**) were created with The PyMOL Molecular Graphics System, Version 2.4.1, Schroödinger, LLC. Two-dimensional figures (**B**,**D**) were generated with Maestro 12.3 (Schrödinger Release, Schrödinger, LLC, New York, NY, USA, 2020).

Molecular dynamic simulation was used to determine the binding differences between the topological isomers **5** (*h*BuChE inactive at 100 μM) and **6** (*h*BuChE IC_50_ = 72 nM) in the *h*BuChE active site (PDB ID: 6QAA) [43]. As portrayed in Figure 3A,B, compound **5** is accommodated in a “wrapped” manner. The critical interaction can be classified as parallel π-π stacking between the *A* phenyl ring and Tyr332 (3.5 Å), and the salt bridge formed between the carboxyl of Asp70 and the protonated tertiary amino group (4.8 Å). Two hydrogen bonds with water molecules are also apparent. One can be observed with oxygen from the ligand’s methoxy group (2.4 Å); the second is mediated to the protonated tertiary amino group (1.9 Å). The benzyloxy group is implicated in the formation of T-shaped π-π interactions with Trp82 (4.7 Å) and His438 (4.8 Å). The latter residue is part of the enzyme’s catalytic machinery. The other two catalytic triad residues, namely Ser198 and Glu325, stand aside from the ligand anchoring. The allyl group seems to protrude outside the cavity gorge providing no specific interaction with the enzyme at all. Ring *B* of ligand **5** occupies the oxyanion hole of the enzyme flanked by Gly116, Gly117, and Ala199. The allyloxy appendage contacts the acyl binding pocket (Val288, Leu286) of the enzyme via hydrophobic interaction, and Trp231 by aliphatic-π interaction.

For the 6-*h*BuChE complex (Figure 3C,D), the ligand is accommodated in a more „straight” manner compared to the lodging of **5** with the enzyme cavity. The protonated amino center of **6** is anchored by cation-π to Tyr332 (5.6 Å) and one water molecule (1.9 Å). The benzyloxy moiety orchestrates several hydrophobic interactions, e.g., π-π stackings to Trp231 (T-shaped; 3.6 Å), Phe329 (distorted; 4.2 Å), and Phe398 (distorted; 3.7 Å). Besides, the benzyloxy moiety is also surrounded by residues from the acyl pocket (Leu286, Val288) and oxyanion hole residues (Gly116, Gly117, Ala199). Moreover, π-π contact to His438 (4.4 Å) and van der Waals forces with Ser198 from the catalytic triad can also be observed. Phenyl ring *A* faces Trp82 (4.1 Å) via π-π interaction. The methoxy group attached to phenyl ring *A* is implicated in a hydrogen bridge with one water molecule. Phenyl ring *B* is oriented distally being exposed to Ala277, Ile69, and Asp70 residues.

**Figure 4:**
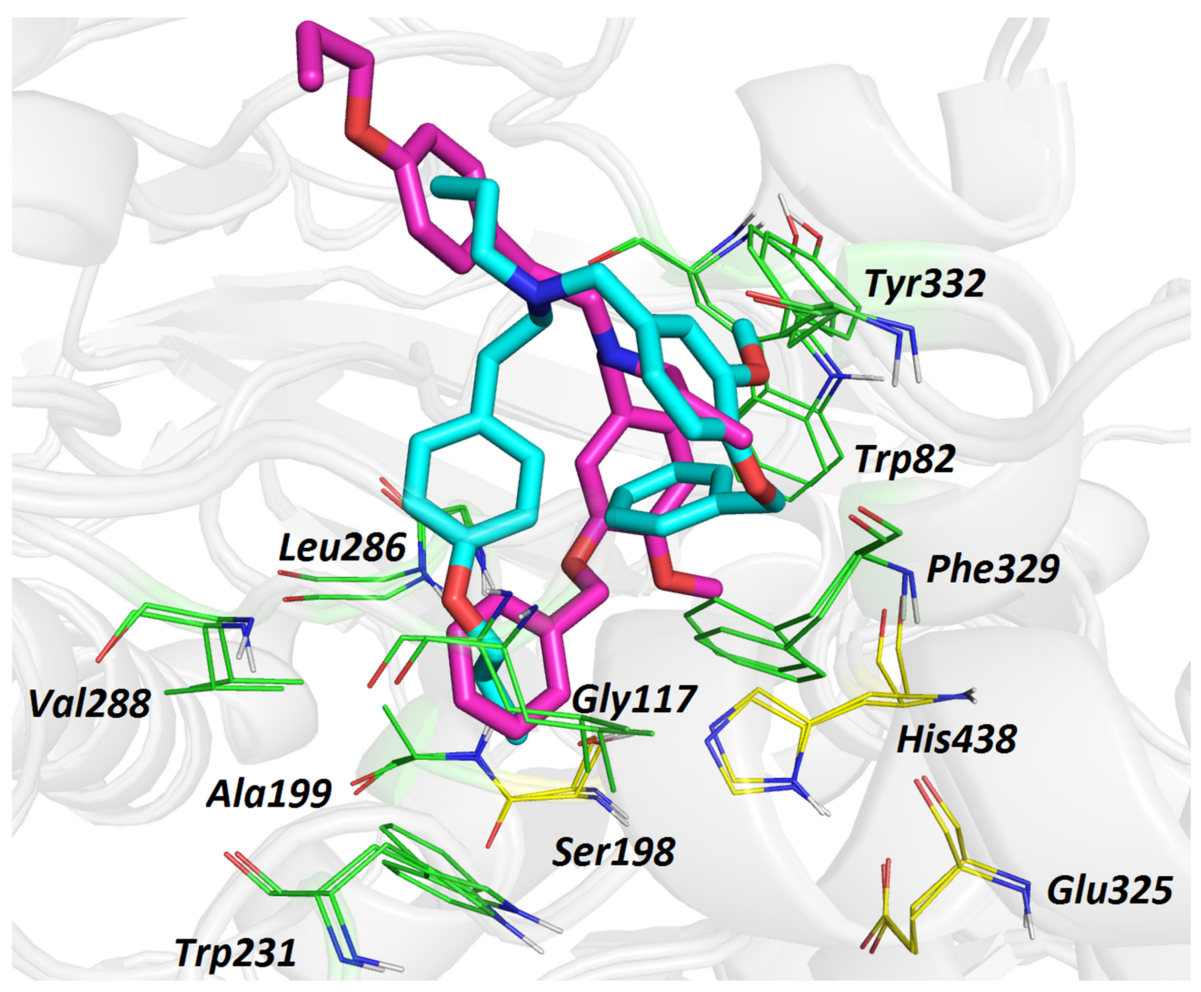
Overlapped top-scored poses for ligands **5** (light blue) and **6** (purple) in the active site of *h*BuChE (PDB ID: 6QAA) [43]. Amino acid residues involved in the interactions with ligands are depicted as either green or yellow (catalytic triad) lines. The rest of the receptor is displayed in light-grey cartoon conformation. Figure was created with The PyMOL Molecular Graphics System, Version 2.4.1, Schroödinger, LLC.

From the MD simulation, it can be concluded that the higher inhibition ability of **6** can be ascribed to the accommodation of its benzyloxy substituent, revealing several crucial interactions with the enzyme. The inability of **5** to lodge in the *h*BuChE active site, like **6**, led to less energetically favored overall topology, i.e., the “wrapped” conformation. For the sake of clarity, we are providing the superimposed spatial view of **5** and **6** (Figure 4), demonstrating their different orientation in the *h*BuChE cavity.

### 3.7. Cytotoxicity of Compound ***6***

The cytotoxicity profile was assessed for compound **6** using the human neuroblastoma SH-SY5Y cell line and MTT assay. At a concentration of 10 µM, compound **6** was completely devoid of cytotoxicity, with viability reaching 95.1 ± 2.6%.

### 3.8. MAO-A and MAO-B Inhibition Potency of Compound ***6***

Since monoamine oxidase (MAO; EC 1.4.3.4) inhibitors are considered as neuroprotective agents, compound **6** was screened at two concentrations (1 and 10 µM) for its inhibition potential against both isoforms of human MAOs. Compound **6** displayed only weak MAO-A and MAO-B inhibition activity (residual activity of enzymes after treatment by **6** was higher than 68% in both concentrations; IC_50_ >10 µM).

## 4. Conclusions

The development of highly selective BuChE inhibitors represents an emerging field of research [44]. There is strong evidence that inhibiting BuChE could benefit AD patients in more progressive stages of the disease compared to AChE-selective inhibitors [45]. Moreover, selective BuChE inhibitors do not exert any adverse effects mediated by the enzyme inhibition. Indeed, it has been reported that selective inhibition of BuChE with cymserine analogs raises ACh levels in the brain and improves cognitive performance without any adverse parasympathetic side effects [45,46]. To date, only a few studies have reported the discovery of selective, reversible BuChE inhibitors [43,44,47,48]. Within this work, we have constructed 20 novel compounds based on the norbelladine framework using carltonine A and B as template scaffolds. The compounds were generated by applying straightforward synthetic routes with moderate to excellent yields. The majority of the developed compounds displayed a highly selective inhibition pattern for *h*BuChE with IC_50_ values below 1 µM. The most selective of them, compound **6**, exerted nanomolar scale inhibition with an IC_50_ of 72 nM. The mode of *h*BuChE inhibition was minutely inspected using enzyme kinetic analysis in tandem with MD simulation. The latter pointed out the differences in the binding modes between *h*BuChE inactive inhibitor **5** and its isomer, top-ranked compound **6**. We have also predicted the CNS availability by applying BBB score calculation, presuming that compounds can pass through the BBB. The observations made herein should pave the way for the structure-based optimization of further norbelladine analogues, potentially applicable in the treatment of neurodegenerative diseases.

## 5. Materials and Methods

### 5.1. General Experimental Procedures

All solvents were treated by using standard techniques before use. All reagents and catalysts were purchased from commercial sources (Sigma Aldrich, Prague, Czech Republic) and used without purification. The NMR spectra were obtained in CDCl_3_ at ambient temperature on a VNMR S500 (Varian) spectrometer operating at 500 MHz for ^1^H and 125.7 MHz for ^13^C and on a JNM-ECZ600R (Jeol) instrument operating at 600 MHz for ^1^H and 151 MHz for ^13^C. Chemical shifts were recorded as *δ* values in parts per million (ppm) and were indirectly referenced to tetramethylsilane (TMS) via the solvent signal (CDCl_3_–7.26 ppm for ^1^H and 77.0 ppm for ^13^C). Coupling constants (*J*) are given in Hz. ESI-HRMS were obtained with a Waters Synapt G2-Si hybrid mass analyzer of a quadrupole-time-of-flight (Q-TOF) type, coupled to a Waters Acquity I-Class UHPLC system. TLC was carried out on Merck precoated silica gel 60 F254 plates. Compounds on the plate were observed under UV light (254 and 366 nm) and visualized by spraying with Dragendorff’s reagent.

### 5.2. Preparation of Carltonine Derivatives: General Procedure for Preparation of Compounds ***1***, ***2***, ***7***, ***8***, ***11***, ***12***, ***15***, and ***18***

One hundred mg of the required aromatic aldehyde (0.41 mmol of *O*-benzylvanillin/*O*-benzylisovanillin; 0.55 mmol of 3-ethoxy-4-methoxybenzaldehyde) was dissolved in 5 mL of methanol, the corresponding amine (0.28–0.38 mmol) was added, and the reaction was stirred for one day. Sodium borohydride (0.28–0.38 mmol) was added to the reaction mixture at 0 °C and the mixture was stirred for a further 3 h at room temperature. The reaction mixture was evaporated to dryness and separated by preparative thin-layer chromatography (To:cHx:Et_2_NH 5:10:2). The NMR spectra for known compounds **1** and **2** [37,49] matched those in the literature. The characterization of all new compounds is given below.

*N*-(4-benzyloxy-3-methoxybenzyl)-2-(4-hydroxyphenyl)ethan-1-amine (**1**) spectra in accordance with literature [49]. Yield: 143 mg (95%), white amorphous solid.

*N*-(3-benzyloxy-4-methoxybenzyl)-2-(4-hydroxyphenyl)ethan-1-amine (**2**) spectra in accordance with literature [37,49]. Yield: 116 mg (78%), white amorphous solid.

#### 5.2.1. *N*-(4-benzyloxy-3-methoxybenzyl)-2-phenylethan-1-amine (**7**)

Yield: 115 mg (80%); white amorphous solid; ^1^H NMR (600 MHz, CDCl_3_) δ: 7.43–7.41 (m, 2H), 7.36–7.32 (m, 2H), 7.31–7.24 (m, 3H), 7.21–7.17 (m, 3H), 6.84 (d, *J* = 2.0 Hz, 1H), 6.80 (d, *J* = 8.1 Hz, 1H), 6.72 (dd, *J* = 8.1 Hz, *J* = 2.0 Hz, 1H), 5.12 (s, 2H), 3.86 (s, 3H), 3.72 (s, 2H), 2.89 (t, *J* = 7.1 Hz, 2H), 2.81 (t, *J* = 7.1 Hz, 2H); ^13^C NMR (151 MHz, CDCl_3_) δ: 149.7, 147.1, 140.1, 137.3, 133.7, 128.7, 128.5, 128.4, 127.8, 127.3, 126.1, 120.1, 114.0, 111.8, 71.2, 56.0, 53.6, 50.5, 36.3; ESI-HRMS *m*/*z* calcd for C_23_H_25_NO_2_ [M+H]^+^: 348.1958, found 348.1962.

#### 5.2.2. *N*-(3-benzyloxy-4-methoxybenzyl)-2-phenylethan-1-amine (**8**)

Yield: 109 mg (76%); beige amorphous solid; ^1^H NMR (500 MHz, CDCl_3_) *δ*: 7.48–7.44 (m, 2H), 7.39–7.34 (m, 2H), 7.32–7.28 (m, 3H), 7.24–7.17 (m, overlap, 3H), 7.93 (s, 1H), 6.86 (d, *J* = 8.1 Hz, overlap, 1H), 6.83 (d, *J* = 8.1 Hz, overlap, 1H), 5.15 (s, 2H), 3.86 (s, 3H), 3.74 (s, 2H), 3.00–2.65 (m, 5H); ^13^C NMR (126 MHz, CDCl_3_) *δ*: 148.9, 148.1, 139.6, 137.1, 131.5, 128.7, 128.5, 128.5, 127.8, 127.4, 126.2, 121.1, 114.2, 111.7, 70.9, 56.0, 53.1, 49.9, 35.8; ESI-HRMS *m*/*z* calcd for C_23_H_25_NO_2_ [M+H]^+^: 348.1958, found 348.1961.

#### 5.2.3. {[4-(benzyloxy)-3-methoxyphenyl]methyl}[2-(4-methoxyphenyl)ethyl]amine (**11**)

Yield: 124 mg (80%); white amorphous solid; ^1^H NMR (600 MHz, CHCl_3_) δ: 7.43–7.40 (m, 2H), 7.36–7.32 (m, 2H), 7.30–7.26 (m, 1H), 7.13–7.07 (m, AA′BB′, 2H), 6.87 (s, 1H), 6.83–6.79 (m, AA′BB′, 2H), 6.79 (d, *J* = 8.2 Hz, 1H), 6.73 (d, *J* = 8.2 Hz, 1H), 5.11 (s, 2H), 3.86 (s, 3H), 3.77 (s, 3H), 3.72 (s, 2H), 2.85 (t, *J* = 6.9 Hz, 2H), 2.77 (t, *J* = 6.9 Hz, 2H); ^13^C NMR (151 MHz, CDCl_3_) δ: 158.1, 149.7, 147.3, 137.3, 131.8, 129.6, 128.5, 127.7, 127.2, 120.3, 114.0, 113.9, 111.2, 71.1, 56.0, 55.2, 53.4, 50.5, 35.1; ESI-HRMS *m*/*z* calcd for C_24_H_27_NO_3_ [M+H]^+^: 378.2064, found 378.2067.

#### 5.2.4. *N*-(3-benzyloxy-4-methoxybenzyl)-2-(4-methoxyphenyl)ethan-1-amine (**12**)

Yield: 115 mg (74%); white amorphous solid; ^1^H NMR (500 MHz, CDCl_3_) δ: 7.47–7.44 (m, 2H), 7.39–7.34 (m, 2H), 7.32–7.28 (m, 1H), 7.13–7.08 (m, AA′BB′, 2H), 6.91 (s, 1H), 6.86–6.80 (m, 4H), 5.14 (s, 2H), 3.86 (s, 3H), 3.78 (s, 3H), 3.72 (s, 2H), 2.82 (t, *J* = 7.0 Hz, 2H), 2.77 (t, *J* = 7.0 Hz, 2H); ^13^C NMR (126 MHz, CDCl_3_) δ: 158.1, 148.9, 148.1, 137.1, 131.6, 129.6, 128.5, 127.8, 127.4, 121.1, 114.2, 113.9, 111.7, 70.9, 56.0, 55.2, 53.1, 50.1, 34.9; ESI-HRMS *m*/*z* calcd for C_24_H_27_NO_3_ [M+H]^+^: 378.2064, found 378.2068.

#### 5.2.5. *N*-(3-ethoxy-4-methoxybenzyl)-2-(4-hydroxyphenyl)ethan-1-amine (**15**)

Yield: 139 mg (84%); white amorphous solid; ^1^H NMR (600 MHz, CDCl_3_) δ: 7.02–6.97 (m, AA′BB′, 2H), 6.81 (s, 1H), 6.80–6.74 (m, 2H), 6.69–6.65 (m, AA′BB′, 2H), 4.00 (q, *J* = 7.0 Hz, 2H), 3.83 (s, 3H), 3.72 (s, 2H), 2.86 (t, *J* = 7.0 Hz, 2H), 2.74 (t, *J* = 7.0 Hz, 2H), 1.40 (t, *J* = 7.0 Hz, 3H); ^13^C NMR (151 MHz, CDCl_3_) δ: 154.7, 148.4, 148.3, 132.2, 131.1, 129.8, 120.4, 115.5, 112.9, 111.3, 64.2, 56.0, 53.5, 50.3, 35.0, 14.8; ESI-HRMS *m*/*z* calcd for C_18_H_23_NO_3_ [M+H]^+^: 302.1751, found 302.1761.

#### 5.2.6. *N*-(3-ethoxy-4-methoxybenzyl)-2-phenylethan-1-amine (**18**)

Yield: 120 mg (76%); white amorphous solid; ^1^H NMR (500 MHz, CDCl_3_) δ: 7.33–7.26 (m, 2H), 7.25–7.18 (m, 3H), 6.85 (s, 1H), 6.81 (bs, 2H), 4.09 (q, *J* = 7.0 Hz, 2H), 3.86 (s, 3H), 3.75 (s, 2H), 2.91 (t, *J* = 6.1 Hz, 2H), 2.85 (t, *J* = 6.1 Hz, 2H), 1.47 (t, *J* = 7.0 Hz, 3H); ^13^C NMR (126 MHz, CDCl_3_) δ: 148.3, 139.9, 132.5, 128.7, 128.4, 126.1, 120.2, 112.7, 111.3, 64.2, 56.0, 53.5, 50.3, 36.2, 14.8; ESI-HRMS *m*/*z* calcd for C_18_H_23_NO_2_ [M+H]^+^: 286.1802, found 286.1805.

### 5.3. Preparation of Carltonine Derivatives: General Procedure for Acylation to Give Compounds ***3**–**6***, ***9***, ***10***, ***13***, ***14***, ***16***, ***17***, ***19***, ***20***

The appropriate substituted *N*-benzyl-2-phenylethylamine derivative (70 mg of **1**, **2**, **7**, **8**, **11**, **12**, and **15**; 30 mg of **18**; 20 mg of **3**, **4**, and **16**) was dissolved in dry THF (2 mL). Sodium hydride (1.2 eq., 60% suspension in mineral oil) was added to the solution at 0 °C, stirred under argon, and after 30 min either allyl- or benzyl bromide (1.2 eq.) was added. The reaction was left stirring under argon for one day at rt. Then, the reaction mixture was evaporated to dryness and separated by preparative thin layer chromatography (cHx:Et_2_NH 9:1). The characterization of all new compounds is given below.

#### 5.3.1. *N*-allyl-*N*-(4-benzyloxy-3-methoxybenzyl)-2-(4-hydroxyphenyl)ethan-1-amine (**3**)

Yield: 46 mg (60%); brown amorphous solid; ^1^H NMR (600 MHz, CDCl_3_) δ: 7.44–7.41 (m, 2H), 7.37–7.32 (m, 2H), 7.30–7.26 (m, 1H), 6.95–6.94 (m, AA′BB′, 2H), 6.90 (d, *J* = 1.6 Hz, 1H), 6.79 (d, *J* = 8.0 Hz, 1H), 6.73 (dd, *J* = 8.0 Hz, *J* = 1.6 Hz, 1H), 6.70–6.64 (m, AA′BB′, 2H), 5.88 (ddt, *J* = 16.0 Hz, *J* = 11.4 Hz, *J* = 6.4 Hz, 1H), 5.19 (d, *J* = 16.0 Hz, 1H), 5.15 (d, *J* = 11.4 Hz, overlap, 1H), 5.12 (s, overlap, 2H), 3.81 (s, 3H), 3.59 (s, 2H), 3.16 (d, *J* = 6.4 Hz, 2H), 2.71–2.68 (m, 4H); ^13^C NMR (151 MHz, CDCl_3_) *δ*: 154.0, 149.6, 147.1, 137.3, 135.3, 132.2, 132.1, 129.8, 128.5, 127.8, 127.3, 121.1, 117.7, 115.2, 113.8, 112.6, 71.1, 57.7, 56.5, 55.9, 55.0, 32.1; ESI-HRMS *m*/*z* calcd for C_26_H_29_NO_3_ [M+H]^+^: 404.2220, found 404.2220.

#### 5.3.2. *N*-allyl-*N*-(3-benzyloxy-4-methoxybenzyl)-2-(4-hydroxyphenyl)ethan-1-amine (**4**)

Yield: 40 mg (52%); white amorphous solid; ^1^H NMR (500 MHz, CDCl_3_) *δ*: 7.45–7.41 (m, 2H), 7.37–7.32 (m, 2H), 7.30–7.26 (m, 1H), 6.98–6.92 (m, 3H), 6.85 (d, *J* = 8.5 Hz, overlap, 1H), 6.83 (d, *J* = 8.5 Hz, overlap, 1H), 6.74–6.69 (m, AA′BB′, 2H), 5.86 (ddt, *J* = 16.8 Hz, *J* = 10.1 Hz, *J* = 6.4 Hz, 1H), 5.22–5.14 (m, 2H), 5.09 (s, 2H), 3.88 (s, 3H), 3.60 (s, 2H), 3.15 (d, *J* = 6.4 Hz, 2H), 2.71–2.63 (m, 4H); ^13^C NMR (126 MHz, CDCl_3_) *δ*: 154.2, 148.8, 148.0, 137.2, 134.8, 131.8, 130.8, 129.7, 128.4, 127.7, 127.4, 121.8, 118.0, 115.3, 114.9, 111.5, 70.8, 57.5, 56.2, 56.1, 54.9, 32.0; ESI-HRMS *m*/*z* calcd for C_26_H_29_NO_3_ [M+H]^+^: 404.2220, found 404.2221.

#### 5.3.3. *N*-allyl-*N*-(4-benzyloxy-3-methoxybenzyl)-2-(4-allyloxyphenyl)ethan-1-amine (**5**)

Yield: 12 mg (55%); white amorphous solid; ^1^H NMR (500 MHz, CDCl_3_) δ: 7.48–7.45 (m, 2H), 7.40–7.36 (m, 2H), 7.34–7.29 (m, 1H), 7.06–7.02 (m, AA′BB′, 2H), 6.91 (d, *J* = 1.9 Hz, 1H), 6.84–6.81 (m, AA′BB′, overlap, 2H), 6.81 (d, *J* = 8.1 Hz, overlap, 1H), 6.75 (dd, *J* = 8.1 Hz, *J* = 1.9 Hz, 1H), 6.06 (ddt, *J* = 17.3 Hz, *J* = 10.5 Hz, *J* = 5.4 Hz, 1H), 5.88 (ddt, *J* = 16.7 Hz, *J* = 10.2 Hz, *J* = 6.3 Hz, 1H), 5.40 (dq, *J* = 17.3 Hz, *J* = 1.4 Hz, 1H), 5.27 (dq, *J* = 10.5 Hz, *J* = 1.4 Hz, 1H), 5.21 (d, *J* = 16.7 Hz, *J* = 1.4 Hz, 1H), 5.19 –5.16 (m, overlap, 1H), 5.15 (s, overlap, 2H), 4.51 (dt, *J* = 5.4 Hz, *J* = 1.4 Hz, 2H), 3.86 (s, 3H), 3.58 (s, 2H), 3.15 (d, *J* = 6.3 Hz, 2H), 2.77–2.65 (m, 4H); ^13^C NMR (151 MHz, CDCl_3_) δ: 156.8, 149.6, 147.0, 137.4, 136.0, 133.5, 132.9, 132.9, 129.6, 128.5, 127.7, 127.3, 120.7, 117.5, 117.1, 114.5, 113.8, 112.3, 71.1, 68.8, 57.8, 56.7, 55.9, 55.2, 32.5; ESI-HRMS *m*/*z* calcd for C_29_H_33_NO_3_ [M+H]^+^: 444.2533, found 444.2531.

#### 5.3.4. *N*-allyl-*N*-(3-benzyloxy-4-methoxybenzyl)-2-(4-allyloxyphenyl)ethan-1-amine (**6**)

Yield: 11 mg (50%); white amorphous solid; ^1^H NMR (500 MHz, CDCl_3_) δ: 7.46–7.42 (m, 2H), 7.37–7.33 (m, 2H), 7.30–7.26 (m, 1H), 7.06–7.01 (m, AA′BB′, 2H), 6.91 (s, 1H), 6.84–6.77 (m, 4H), 6.04 (ddt, *J* = 17.2 Hz, *J* = 10.3 Hz, *J* = 5.3 Hz, 1H), 5.86 (ddt, *J* = 16.6 Hz, *J* = 10.2 Hz, *J* = 6.3 Hz, 1H), 5.40 (dq, *J* = 17.2 Hz, *J* = 1.6 Hz, 1H), 5.26 (dq, *J* = 10.3 Hz, *J* = 1.6 Hz, 1H), 5.14 (d, *J* = 17.2 Hz, *J* = 1.6 Hz, 1H), 5.13–5.09 (m, overlap, 1H), 5.10 (s, overlap, 2H), 4.47 (dt, *J* = 5.3 Hz, *J* = 1.6 Hz, 2H), 3.89 (s, 3H), 3.53 (s, 2H), 3.08 (dt, *J =* 6.3 Hz, *J* = 1.4 Hz, 2H), 2.78–2.62 (m, 4H); ^13^C NMR (151 MHz, CDCl_3_) δ: 156.8, 148.6, 148.0, 137.3, 136.0, 133.5, 132.9, 132.1, 129.6, 128.4, 127.7, 127.4, 121.3, 117.5, 117.0, 114.6, 114.5, 111.4, 70.9, 68.8, 57.6, 56.6, 56.0, 55.1, 32.5.; ESI-HRMS *m*/*z* calcd for C_29_H_33_NO_3_ [M+H]^+^: 444.2533, found 444.2540.

#### 5.3.5. *N*-allyl-*N*-(4-benzyloxy-3-methoxybenzyl)-2-phenylethan-1-amine (**9**)

Yield: 40 mg (51%); white amorphous solid; ^1^H NMR (500 MHz, CDCl_3_) δ: 7.48–7.44 (m, 2H), 7.40–7.36 (m, 2H), 7.34–7.30 (m, 1H), 7.29–7.25 (m, 2H), 7.21–7.14 (m, 3H), 6.91 (d, *J* = 1.9 Hz, 1H), 6.82 (d, *J* = 8.1 Hz, 1H), 6.76 (dd, *J* = 8.1 Hz, *J* = 1.9 Hz, 1H), 5.89 (ddt, *J* = 16.7 Hz, *J* = 10.3 Hz, *J* = 6.3 Hz, 1H), 5.18 (dq, *J* = 16.7 Hz, *J* = 1.7 Hz, 1H), 5.17–5.13 (m, overlap, 1H), 5.16 (s, overlap, 2H), 3.87 (s, 3H), 3.60 (s, 2H), 3.17 (d, *J* = 6.3 Hz, 2H), 2.83–2.77 (m, 2H), 2.77–2.70 (m, 2H); ^13^C NMR (126 MHz, CDCl_3_) δ: 149.6, 147.0, 140.7, 137.4, 135.9, 132.8, 128.8, 128.5, 128.2, 127.7, 127.3, 125.8, 120.7, 117.2, 113.7, 112.3, 71.1, 57.8, 56.7, 55.9, 54.9, 33.4; ESI-HRMS *m*/*z* calcd for C_26_H_29_NO_2_ [M+H]^+^: 388.2271, found 388.2278.

#### 5.3.6. *N*-allyl-*N*-(3-benzyloxy-4-methoxybenzyl)-2-phenylethan-1-amine (**10**)

Yield: 32 mg (41%); white amorphous solid; ^1^H NMR (600 MHz, CDCl_3_) *δ*: 7.45–7.42 (m, 2H), 7.36–7.32 (m, 2H), 7.31–7.22 (m, 3H), 7.18–7.15 (m, 1H), 7.14–7.11 (m, 2H), 6.91 (s, 1H), 6.82 (d, *J* = 8.4 Hz, overlap, 1H), 6.81 (d, *J* = 8.4 Hz, overlap, 1H), 5.81 (ddt, *J* = 17.0 Hz, *J* = 10.2 Hz, *J* = 6.4 Hz, 1H), 5.14 (dq, *J* = 17.0 Hz, *J* = 1.6 Hz, 1H), 5.12–5.08 (m, overlap, 1H), 5.10 (s, overlap, 2H), 3.87 (s, 3H), 3.54 (s, 2H), 3.09 (d, *J* = 6.4 Hz, 2H), 2.77–2.71 (m, 2H), 2.71–2.65 (m, 2H); ^13^C NMR (151 MHz, CDCl_3_) *δ*: 148.6, 148.0, 140.7, 137.3, 135.9, 132.1, 128.8, 128.4, 128.2, 127.7, 127.4, 125.8, 121.4, 117.1, 114.6, 111.5, 70.9, 57.6, 56.6, 56.1, 54.9, 33.4; ESI-HRMS *m*/*z* calcd for C_26_H_29_NO_2_ [M+H]^+^: 388.2271, found 388.2279.

#### 5.3.7. *N*-allyl-*N*-(4-benzyloxy-3-methoxybenzyl)-2-(4-methoxyphenyl)ethan-1-amine (**13**)

Yield: 35 mg (44%); white amorphous solid; ^1^H NMR (500 MHz, CDCl_3_) δ: 7.47–7.44 (m, 2H), 7.39–7.35 (m, 2H), 7.33–7.29 (m, 1H), 7.08–7.03 (m, AA′BB′, 2H), 6.90 (d, *J* = 1.6 Hz, 1H), 6.83–6.79 (m, 3H), 6.75 (dd, *J* = 8.1 Hz, *J* = 1.6 Hz, 1H), 5.88 (ddt, *J* = 17.0 Hz, *J* = 10.1 Hz, *J* = 6.4 Hz, 1H), 5.20 (dq, *J* = 17.0 Hz, *J* = 1.5 Hz, 1H), 5.16–5.12 (m, overlap, 1H), 5.15 (s, overlap, 2H), 3.86 (s, 3H), 3.79 (s, 3H), 3.58 (s, 2H), 3.15 (d, *J* = 6.4 Hz, 2H), 2.77–2.64 (m, 4H); ^13^C NMR (126 MHz, CDCl_3_) δ: 157.8, 149.6, 147.0, 137.4, 135.9, 132.9, 132.7, 129.6, 128.5, 127.7, 127.3, 120.8, 117.1, 113.7, 113.7, 112.4, 71.1, 57.8, 56.7, 55.9, 55.2, 55.2, 32.5; ESI-HRMS *m*/*z* calcd for C_27_H_31_NO_3_ [M+H]^+^: 418.2377, found 418.2390.

#### 5.3.8. *N*-allyl-*N*-(3-benzyloxy-4-methoxybenzyl)-2-(4-methoxyphenyl)ethan-1-amine (**14**)

Yield: 32 mg (42%); brown amorphous solid; ^1^H NMR (500 MHz, CDCl_3_) *δ*: 7.48–7.44 (m, 2H), 7.39–7.34 (m, 2H), 7.32–7.28 (m, 1H), 7.08–7.02 (m, AA′BB′, 2H), 6.93 (s, 1H), 6.84–6.79 (m, AA′BB′, overlap, 2H), 6.83 (d, *J* = 8.3 Hz, overlap, 1H), 6.82 (d, *J* = 8.3 Hz, overlap, 1H), 5.83 (ddt, *J* = 16.7 Hz, *J* = 10.4 Hz, *J* = 6.5 Hz, 1H), 5.18–5.10 (m, overlap, 2H), 5.12 (s, overlap, 2H), 3.89 (s, 3H), 3.77 (s, 3H), 3.55 (s, 2H), 3.10 (d, *J* = 6.5 Hz, 2H), 2.73–2.61 (m, 4H); ^13^C NMR (126 MHz, CDCl_3_) *δ*: 157.8, 148.6, 148.0, 137.3, 136.0, 132.7, 132.1, 129.6, 128.4, 127.7, 127.4, 121.4, 117.0, 114.6, 113.6, 111.4, 70.9, 57.6, 56.6, 56.1, 55.2, 55.1, 32.5; ESI-HRMS *m*/*z* calcd for C_27_H_31_NO_3_ [M+H]^+^: 418.2377, found 418.2381.

#### 5.3.9. *N*-allyl-*N*-(3-ethoxy-4-methoxybenzyl)-2-(4-hydroxyphenyl)ethan-1-amine (**16**)

Yield: 139 mg (84%); white amorphous solid; ^1^H NMR (500 MHz, CDCl_3_) δ: 7.03–6.96 (m, AA′BB′, 2H), 6.88 (s, 1H), 6.84–6.77 (m, 2H), 6.75–6.69 (m, AA′BB′, 2H), 5.89 (ddt, *J* = 16.9 Hz, *J* = 10.3 Hz, *J* = 6.3 Hz, 1H), 5.20 (dq, *J* = 16.9 Hz, *J* = 1.7 Hz, 1H), 5.15 (dq, *J* = 10.3 Hz, *J* = 1.7 Hz, 1H), 4.05 (q, *J* = 7.0 Hz, 2H), 3.87 (s, 3H), 3.58 (s, 2H), 3.16 (d, *J* = 6.3 Hz, 2H), 2.75–2.64 (m, 4H), 1.45 (t, *J* = 7.0 Hz, 3H); ^13^C NMR (126 MHz, CDCl_3_) δ: 153.8, 148.1, 135.8, 132.6, 131.9, 129.8, 120.9, 117.3, 115.1, 113.3, 111.0, 64.2, 57.8, 56.6, 56.0, 55.1, 32.4, 14.8; ESI-HRMS *m*/*z* calcd for C_21_H_27_NO_3_ [M+H]^+^: 342.2064, found 364.2073.

#### 5.3.10. *N*-allyl-*N*-(3-ethoxy-4-methoxybenzyl)-2-(4-allyloxyphenyl)ethan-1-amine (**17**)

Yield: 11 mg (50 %); white amorphous solid; ^1^H NMR (500 MHz, CDCl_3_) δ: 7.09–7.02 (m, AA’BB’, 2H), 6.88 (s, 1H), 6.85–6.77 (m, 4H), 6.06 (ddt, *J* = 17.3 Hz, *J* = 10.5 Hz, *J* = 5.3 Hz, 1H), 5.88 (ddt, *J* = 16.7 Hz, *J* = 10.2 Hz, *J* = 6.4 Hz, 1H), 5.42 (dq, *J* = 17.3 Hz, *J* = 1.5 Hz, 1H), 5.28 (dq, *J* = 10.5 Hz, *J* = 1.5 Hz, 1H), 5.20 (dq, *J* = 16.7 Hz, *J* = 1.7 Hz, 1H), 5.14 (dd, *J* = 10.2 Hz, *J* = 1.7 Hz, 1H), 4.52 (dt, *J* = 5.3 Hz, *J* = 1.5 Hz, 2H), 4.07 (q, *J* = 7.0 Hz, 2H), 3.87 (s, 3H), 3.58 (s, 2H), 3.15 (d, *J* = 6.4 Hz, 2H), 2.77–2.65 (m, 4H), 1.47 (t, *J* = 7.0 Hz, 3H); ^13^C NMR (126 MHz, CDCl_3_) δ: 156.8, 148.1, 136.0, 133.5, 132.9, 132.1, 129.6, 120.8, 117.5, 117.1, 114.5, 113.2, 111.0, 68.8, 64.1, 57.7, 56.6, 55.9, 55.1, 32.5, 14.8; ESI-HRMS *m*/*z* calcd for C_24_H_31_NO_3_ [M+H]^+^: 382.2377, found 382.2378.

#### 5.3.11. *N*-allyl-*N*-(3-ethoxy-4-methoxybenzyl)-2-phenylethan-1-amine (**19**)

Yield: 25 mg (70%); white amorphous solid; ^1^H NMR (500 MHz, CDCl_3_) δ: 7.29–7.24 (m, 2H), 7.21–7.17 (m, 1H), 7.17–7.13 (m, 2H), 6.89 (s, 1H), 6.81 (d, *J* = 8.7 Hz, overlap, 1H), 6.80 (d, *J* = 8.7 Hz, overlap, 1H), 5.89 (ddt, *J* = 16.9 Hz, *J* = 10.0 Hz, *J* = 6.3 Hz, 1H), 5.20 (dq, *J* = 16.9 Hz, *J* = 2.0 Hz, 1H), 5.15 (dq, *J* = 10.0 Hz, *J* = 2.0 Hz, 1H), 4.06 (q, *J* = 7.0 Hz, 2H), 3.87 (s, 3H), 3.59 (s, 2H), 3.16 (d, *J* = 6.3 Hz, 2H), 2.83–2.76 (m, 2H), 2.76–2.69 (m, 2H), 1.46 (t, *J* = 7.0 Hz, 3H); ^13^C NMR (126 MHz, CDCl_3_) δ: 148.1, 140.7, 135.9, 132.1, 128.8, 128.2, 125.8, 120.8, 117.1, 113.2, 111.0, 64.1, 57.8, 56.6, 56.0, 54.9, 33.4, 14.8; ESI-HRMS *m*/*z* calcd for C_21_H_27_NO_2_ [M+H]^+^: 326.2115, found 326.2118.

#### 5.3.12. *N*-benzyl-*N*-(3-ethoxy-4-methoxybenzyl)-2-phenylethan-1-amine (**20**)

Yield: 30 mg (71%); yellow amorphous solid; ^1^H NMR (500 MHz, CDCl_3_) δ: 7.32–7.13 (m, 8H), 7.13–7.06 (m, 2H), 6.89 (d, *J* = 1.9 Hz, 1H), 6.83 (dd, *J* = 8.1 Hz, *J* = 1.9 Hz, 1H), 6.79 (d, *J* = 8.1 Hz, 1H), 4.05 (q, *J* = 7.0 Hz, 2H), 3.87 (s, 3H), 3.63 (s, 2H), 3.58 (s, 2H), 2.82 (dd, *J* = 9.4 Hz, *J* = 5.7 Hz, 2H), 2.74–2.70 (m, 2H), 1.47 (t, *J* = 7.0 Hz, 3H); ^13^C NMR (126 MHz, CDCl_3_) δ: 148.1, 140.6, 139.7, 132.3, 128.8, 128.8, 128.7, 128.1, 128.1, 126.7, 125.8, 120.6, 113.1, 111.0, 64.1, 58.0, 57.8, 56.0, 54.9, 33.5, 14.8; ESI-HRMS *m*/*z* calcd for C_25_H_29_NO_2_ [M+H]^+^: 376.2271, found 376.2277.

### 5.4. hAChE and hBuChE Inhibition Assay

The *h*AChE/*h*BuChE activities were determined using a modified method of Ellman [38], as recently described by our group [50]. A detailed description of the assay can be found in Supplementary Materials.

### 5.5. Kinetic Study of hBuChE Inhibition

Kinetic studies employed the same procedure that was previously described [50]. Supplementary Materials provides details of this assay.

### 5.6. POP Inhibition Assay

The previously applied procedure was used [50]. Detailed description of the assay can be found in Supplementary Materials.

### 5.7. MAOs Inhibition Assay

MAO inhibition assay was performed as previously described [51]. Supplementary Materials provides details of this assay.

### 5.8. Cytotoxicity Assay

The neuroblastoma cell line SH-SY5Y (Sigma-Aldrich, St. Louis, MO, USA) was used to evaluate cytotoxicity of the tested compounds. The cells were cultivated in Dublecco’s modified Eagle’s medium (DMEM, Biosera, Nuaille, France) supplemented with 10% fetal bovine serum (FBS, Biosera), 1% penicillin (10,000 U/mL)–streptomycin (10,000 µg/mL) antibiotic solution (Sigma-Aldrich) and 1% non-essential aminoacid solution (Sigma-Aldrich) at 37 °C in a CO_2_ incubator (Forma™ 310 Direct Heat CO_2_ Incubator, ThermoFisher Scientific, MA, USA) and routinely passaged by trypsinization at 75–85% confluence.

The MTT (3-(4,5-dimethylthiazol-2-yl)-2,5-diphenyl-tetraziolium bromide (Sigma–Aldrich) reduction assay was used for measurement of the compounds’ cytotoxicity, according to Mosmann [51]. MTT is a water-soluble tetrazolium salt that is converted to purple formazan by succinate dehydrogenase in mitochondria of viable cells [52,53]. Cell viability was detected after 24-h incubation with the tested substances. For the assay, SH-SY5Y cells were seeded into 96-well plates in 100 µL volume and at a density of 20 × 10^3^. Cells were allowed to attach overnight before the treatment. The stock solutions of tested compounds were prepared in dimethylsulfoxide (DMSO, Sigma-Aldrich), which were serially diluted in DMEM and added to the cells in a 96-well culture plate. The final concentration of DMSO was less than 0.25% per well. After 24 h incubation, the medium containing serially diluted substances was aspirated from each well and replaced by 100 µL of fresh medium containing MTT (0.5 mg/mL). Plates were subsequently incubated at 37 °C in a CO_2_ incubator for 1 h. Medium containing MTT was then aspirated, and formazan dissolved in 100 μL of DMSO. The optical density of each well was measured using a Spark^®^ multimode microplate reader (Tecan Group Ltd., Männedorf, Switzerland) at 570 nm. The cell viability was expressed as a percentage of the untreated control. Each experiment was performed in triplicate and repeated three independent times.

### 5.9. In-Silico Studies

Molecular docking was used for binding poses calculations. The 3D structure ligands were built by OpenBabel, v. 2.3.2 [54] and optimized by Avogadro, v. 1.2.0 using the force fields GAFF [55]. They were converted into pdbqt-format by OpenBabel, v. 2.3.2. The *h*BuChE structure was gained from the RCSB database (PDB ID: 6QAA, crystal structure of the *h*BuChE with (*S*)-2-(butylamino)-*N*-(2-cycloheptylethyl)-3-(1*H*-indol-3-yl)propanamide, resolution 1.90 Å) and prepared for docking by the function DockPrep of the software Chimera, v. 1.14 [56] and by MGLTools, v. 1.5.4 [57]. The docking calculation was made by Vina, v. 1.1.2 as semi-flexible with flexible ligand and rigid receptor [58].

The docking poses of **5** and **6** were improved by MD simulation. The receptor structure was prepared using the software Chimera. The best-scored docking pose was taken as the initial for MD. The force-field parameters for ligands were assessed by Antechamber [59], v. 20.0 using General Amber force-field 2 [60]. MD simulations were carried out by Gromacs, v. 2018.1 [61]. The complex receptor-ligand was solvated in the periodic water box using the TIP3P model [62]. The system was neutralized by adding Na^+^ and Cl^−^ ions to the concentration of 10 nM. The system energy was minimalized and equilibrated in a 100-ps isothermal-isochoric NVT and then a 100-ps isothermal-isobaric NPT phase. Then, a 10-ns MD simulation was run at the temperature of 300 K. The molecular docking and MD results were 3D visualized by the PyMOL Molecular Graphics System, Version 2.4.1, Schrödinger, LLC.

## Figures and Tables

**Table 1:** In-vitro *h*AChE/*h*BuChE, and POP inhibition by new derivatives (**1**–**20**) of norbelladine, and calculation of BBB score.

Compound	%Inhibition *h*AChE ± SEM ^a^	IC_50_, *h*AChE ± SEM (µM) ^b^	% Inhibition *h*BuChE ± SEM ^a^	IC_50_, *h*BuChE ± SEM (µM) ^b^	SI for *h*BuChE ^c^	IC_50_, POP ± SEM (µM) ^b^	BBB Score ^d^
**1**	30.4 ± 2.1	>100	98.7 ± 0.3	0.36 ± 0.03	>277	186 ± 14	4.53
**2**	35.8 ± 1.2	>100	97.7 ± 0.5	0.29 ± 0.02	>348	>79 ^f^	4.53
**3**	20.8 ± 0.9	>100	96.8 ± 1.1	0.61 ± 0.04	>163	>200 ^f^	4.79
**4**	45.2 ± 2.4	>100	97.9 ± 0.6	0.25 ± 0.01	>394	>79 ^f^	4.79
**5**	3.4 ± 0.5	>100	38.9 ± 0.9	>100	-	n.s.	4.87
**6**	10.1 ± 0.6	>100	98.6 ± 0.9	**0.07 ± 0.01**	>1,389	>79 ^f^	4.87
**7**	23.4 ± 2.5	>100	94.5 ± 0.9	1.28 ± 0.05	>78	n.s.	5.15
**8**	12.6 ± 0.5	>100	96.6 ± 0.4	1.10 ± 0.05	>90	n.s.	5.15
**9**	18.8 ± 1.9	>100	74.9 ± 2.4	5.19 ± 0.28	>19	n.s.	5.04
**10**	72.4 ± 1.1	21.5 ± 0.6	92.0 ± 2.4	1.17 ± 0.04	18	n.s.	5.04
**11**	27.9 ± 0.7	>100	93.5 ± 0.3	2.39 ± 0.27	>41	n.s.	4.87
**12**	0.0 ± 0.0	>100	94.6 ± 0.6	1.12 ± 0.11	>89	n.s.	4.87
**13**	32.7 ± 1.6	>100	90.9 ± 1.5	2.72 ± 0.50	>37	n.s.	4.96
**14**	60.9 ± 0.4	37.7 ± 1.7	95.8 ± 0.9	0.38 ± 0.01	98	>200 *	4.96
**15**	25.8 ± 1.3	>100	75.3 ± 0.6	15.06 ± 2.34	>6	n.s.	4.80
**16**	28.3 ± 1.1	>100	91.7 ± 0.4	1.21 ± 0.08	>82	n.s.	5.21
**17**	29.3 ± 3.9	>100	77.0 ± 1.0	9.89 ± 1.37	10	n.s.	5.39
**18**	0.0 ± 0.0	>100	60.0 ± 1.6	41.1 ± 2.6	>2	n.s.	5.53
**19**	5.9 ± 2.1	>100	80.2 ± 0.2	4.63 ± 0.48	>22	n.s.	5.60
**20**	49.5 ± 0.8	>100	82.5 ± 1.0	0.69 ± 0.03	>145	n.s.	5.13
**galantamine ^e^**	98.8 ± 1.1	2.0 ± 0.1	68.2 ± 1.2	29.31 ± 3.49	0.07	n.s.	5.01
**eserine ^e^**	99.8 ± 0.6	0.20 ± 0.01	99.9 ± 0.5	0.30 ± 0.01	0.67	n.s.	5.02
**berberine ^e^**	-	-	-	-	-	142 ± 21	n.s.
**chlorothiazide ^e^**	-	-	-	-	-	-	2.14
**promazine ^e^**	-	-	-	-	-	-	5.64

^a^ Tested at 100 µM compound concentration; ^b^ Compound concentration required to decrease enzyme activity by 50%; the values are the mean ± SEM of three independent measurements, each performed in triplicate; ^c^ Selectivity index for *h*BuChE is determined as ratio *h*AChE IC_50_/*h*BuChE IC_50_; ^d^ calculated using BBB score [42]; ^e^ Reference compound; ^f^ Due to low solubility of compounds in buffer, the presented values correspond to the highest tested concentration; n.s. stands for not studied.

## Data Availability

The descriptive statistics of the results were calculated, and the charts made using either Microsoft Office 365 Excel (Microsoft, Redmond, WA, USA) or GraphPad Prism 7 biostatistics (GraphPad Software, La Jolla, CA, USA) software. In this study, all the values were expressed as arithmetic means with SD of triplicates, unless otherwise noted. The data presented in this study are available within the article or Supplementary Materials.

## Supplementary Materials

The following are available online at https://www.mdpi.com/article/10.3390/ijms22158308/s1. Figure S1. HRMS of N-allyl-N-(4-benzyloxy-3-methoxybenzyl)-2-(4-hydroxyphenyl)ethan-1-amine (3); Figure S2. ^1^H NMR spectrum of N-allyl-N-(4-benzyloxy-3-methoxybenzyl)-2-(4-hydroxyphenyl)ethan-1-amine (3) in CDCl_3_; Figure S3. ^13^C NMR spectrum of N-allyl-N-(4-benzyloxy-3-methoxybenzyl)-2-(4-hydroxyphenyl)ethan-1-amine (3) in CDCl_3_; Figure S4. HRMS of *N*-allyl-*N*-(3-benzyloxy-4-methoxybenzyl)-2-(4-hydroxyphenyl)ethan-1-amine (4); Figure S5. ^1^H NMR spectrum *N*-allyl-*N*-(3-benzyloxy-4-methoxybenzyl)-2-(4-hydroxyphenyl)ethan-1-amine (4) in CDCl_3_; Figure S6. ^13^C NMR spectrum of *N*-allyl-*N*-(3-benzyloxy-4-methoxybenzyl)-2-(4-hydroxyphenyl)ethan-1-amine (4) in CDCl_3_; Figure S7. HRMS of *N*-allyl-*N*-(4-benzyloxy-3-methoxybenzyl)-2-(4-allyloxyphenyl)ethan-1-amine (5); Figure S8. ^1^H NMR spectrum of *N*-allyl-*N*-(4-benzyloxy-3-methoxybenzyl)-2-(4-allyloxyphenyl)ethan-1-amine (5) in CDCl_3_; Figure S9. ^13^C NMR spectrum of of *N*-allyl-*N*-(4-benzyloxy-3-methoxybenzyl)-2-(4-allyloxyphenyl)ethan-1-amine (5) in CDCl_3_; Figure S10. HRMS of *N*-allyl-*N*-(3-benzyloxy-4-methoxybenzyl)-2-(4-allyloxyphenyl)ethan-1-amine (6); Figure S11. ^1^H NMR spectrum of *N*-allyl-*N*-(3-benzyloxy-4-methoxybenzyl)-2-(4-allyloxyphenyl)ethan-1-amine (6) in CDCl_3_; Figure S12. ^13^C NMR spectrum of *N*-allyl-*N*-(3-benzyloxy-4-methoxybenzyl)-2-(4-allyloxyphenyl)ethan-1-amine (6) in CDCl_3_; Figure S13. HRMS of *N*-(4-benzyloxy-3-methoxybenzyl)-2-phenylethan-1-amine (7); Figure S14. ^1^H NMR spectrum of *N*-(4-benzyloxy-3-methoxybenzyl)-2-phenylethan-1-amine (7) in CDCl_3_; Figure S15. ^13^C NMR spectrum of *N*-(4-benzyloxy-3-methoxybenzyl)-2-phenylethan-1-amine (7) in CDCl_3_; Figure S16. HRMS of *N*-(3-benzyloxy-4-methoxybenzyl)-2-phenylethan-1-amine (8); Figure S17. ^1^H NMR spectrum of *N*-(3-benzyloxy-4-methoxybenzyl)-2-phenylethan-1-amine (8) in CDCl_3_; Figure S18. ^13^C NMR spectrum of *N*-(3-benzyloxy-4-methoxybenzyl)-2-phenylethan-1-amine (8) in CDCl_3_; Figure S19. HRMS of *N*-allyl-*N*-(4-benzyloxy-3-methoxybenzyl)-2-phenylethan-1-amine (9); Figure S20. ^13^C NMR spectrum of *N*-allyl-*N*-(4-benzyloxy-3-methoxybenzyl)-2-phenylethan-1-amine (9) in CDCl_3_; Figure S21. ^13^C NMR spectrum of *N*-allyl-*N*-(4-benzyloxy-3-methoxybenzyl)-2-phenylethan-1-amine (9) in CDCl_3_; Figure S22. HRMS of *N*-allyl-*N*-(3-benzyloxy-4-methoxybenzyl)-2-phenylethan-1-amine (10); Figure S23. ^1^H NMR spectrum of *N*-allyl-*N*-(3-benzyloxy-4-methoxybenzyl)-2-phenylethan-1-amine (10) in CDCl_3_; Figure S24. ^13^C NMR spectrum of *N*-allyl-*N*-(3-benzyloxy-4-methoxybenzyl)-2-phenylethan-1-amine (10) in CDCl_3_; Figure S25. HRMS of *N*-(4-benzyloxy-3-methoxybenzyl)-2-(4-methoxyphenyl)ethan-1-amine (11); Figure S26. ^1^H NMR spectrum of *N*-(4-benzyloxy-3-methoxybenzyl)-2-(4-methoxyphenyl)ethan-1-amine (11) in CDCl_3_; Figure S27. ^13^C NMR spectrum of *N*-(4-benzyloxy-3-methoxybenzyl)-2-(4-methoxyphenyl)ethan-1-amine (11) in CDCl_3_; Figure S28. HRMS of *N*-(3-benzyloxy-4-methoxybenzyl)-2-(4-methoxyphenyl)ethan-1-amine (12); Figure S29. ^1^H NMR spectrum of *N*-(3-benzyloxy-4-methoxybenzyl)-2-(4-methoxyphenyl)ethan-1-amine (12) in CDCl_3_; Figure S30. ^13^C NMR spectrum of *N*-(3-benzyloxy-4-methoxybenzyl)-2-(4-methoxyphenyl)ethan-1-amine (12) in CDCl_3_; Figure S31. HRMS of *N*-allyl-*N*-(4-benzyloxy-3-methoxybenzyl)-2-(4-methoxyphenyl)ethan-1-amine (13); Figure S32. ^1^H NMR spectrum of *N*-allyl-*N*-(4-benzyloxy-3-methoxybenzyl)-2-(4-methoxyphenyl)ethan-1-amine (13) in CDCl_3_; Figure S33. ^13^C NMR spectrum of *N*-allyl-*N*-(4-benzyloxy-3-methoxybenzyl)-2-(4-methoxyphenyl)ethan-1-amine (13) in CDCl_3_; Figure S34. HRMS of *N*-allyl-*N*-(3-benzyloxy-4-methoxybenzyl)-2-(4-methoxyphenyl)ethan-1-amine (14); Figure S35. ^1^H NMR spectrum of *N*-allyl-*N*-(3-benzyloxy-4-methoxybenzyl)-2-(4-methoxyphenyl)ethan-1-amine (14) in CDCl_3_; Figure S36. ^13^C NMR spectrum of *N*-allyl-*N*-(3-benzyloxy-4-methoxybenzyl)-2-(4-methoxyphenyl)ethan-1-amine (14) in CDCl_3_; Figure S37. HRMS of *N*-(3-ethoxy-4-methoxybenzyl)-2-(4-hydroxyphenyl)ethan-1-amine (15); Figure S38. ^1^H NMR spectrum of *N*-(3-ethoxy-4-methoxybenzyl)-2-(4-hydroxyphenyl)ethan-1-amine (15) in CDCl_3_; Figure S39. ^13^C NMR spectrum of *N*-(3-ethoxy-4-methoxybenzyl)-2-(4-hydroxyphenyl)ethan-1-amine (15) in CDCl_3_; Figure S40. HRMS of *N*-allyl-*N*-(3-ethoxy-4-methoxybenzyl)-2-(4-hydroxyphenyl)ethan-1-amine (16); Figure S41. ^1^H NMR spectrum of *N*-allyl-*N*-(3-ethoxy-4-methoxybenzyl)-2-(4-hydroxyphenyl)ethan-1-amine (16) in CDCl_3_; Figure S42. ^13^C NMR spectrum of *N*-allyl-*N*-(3-ethoxy-4-methoxybenzyl)-2-(4-hydroxyphenyl)ethan-1-amine (16) in CDCl_3_; Figure S43. HRMS of *N*-allyl-*N*-(3-ethoxy-4-methoxybenzyl)-2-(4-allyloxyphenyl)ethan-1-amine (17); Figure S44. ^1^H NMR spectrum of *N*-allyl-*N*-(3-ethoxy-4-methoxybenzyl)-2-(4-allyloxyphenyl)ethan-1-amine (17) in CDCl_3_; Figure S45. ^13^C NMR spectrum of *N*-allyl-*N*-(3-ethoxy-4-methoxybenzyl)-2-(4-allyloxyphenyl)ethan-1-amine (17) in CDCl_3_; Figure S46. HRMS of *N*-(3-ethoxy-4-methoxybenzyl)-2-phenylethan-1-amine (18); Figure S47. ^1^H NMR spectrum of *N*-(3-ethoxy-4-methoxybenzyl)-2-phenylethan-1-amine (18) in CDCl_3_; Figure S48. ^13^C NMR spectrum of *N*-(3-ethoxy-4-methoxybenzyl)-2-phenylethan-1-amine (18) in CDCl_3_; Figure S49. HRMS of *N*-allyl-*N*-(3-ethoxy-4-methoxybenzyl)-2-phenylethan-1-amine (19); Figure S50. ^1^H NMR spectrum of *N*-allyl-*N*-(3-ethoxy-4-methoxybenzyl)-2-phenylethan-1-amine (19) in CDCl_3_; Figure S51. ^13^C NMR spectrum of N-allyl-N-(3-ethoxy-4-methoxybenzyl)-2-phenylethan-1-amine (19) in CDCl_3_; Figure S52. HRMS of N-benzyl-N-(3-ethoxy-4-methoxybenzyl)-2-phenylethan-1-amine (20); Figure S53. ^1^H NMR spectrum of N-benzyl-N-(3-ethoxy-4-methoxybenzyl)-2-phenylethan-1-amine (20) in CDCl_3_; Figure S54. ^13^C NMR spectrum of N-benzyl-N-(3-ethoxy-4-methoxybenzyl)-2-phenylethan-1-amine (20) in CDCl_3_; Biological assays in detail: hAChE and hBuChE Inhibition Assay; Kinetic Study of Cholinesterase Inhibition; POP inhibition assay; MAOs inhibition assay.

## Sample Availability

Samples of the compounds are available from the authors.
