# Supplementary material for: Amaryllidaceae Alkaloids of Norbelladine-Type as Inspiration for Development of Highly Selective Butyrylcholinesterase Inhibitors: Synthesis, Biological Activity Evaluation, and Docking Studies
Source: Int J Mol Sci. 2021 Aug 2;22(15):8308. doi: 10.3390/ijms22158308 (PMC8348983; doi:10.3390/ijms22158308)
Supplement: Supplementary file 1 [file ijms-22-08308-s001.zip › ijms-1319651-supplementary.pdf]

Supplementary Material

Amaryllidaceae alkaloids of norbelladine-type as inspiration for development of highly selective butyrylcholinesterase inhibitors: synthesis, biological activity evaluation, and docking studies

Abdullah Al Mamun[1], Filip Pidaný[1], Daniela Hulcová[1,2], Jana Maříková[1,3], Tomáš Kučera[4], Monika Schmidt[5], Maria Carmen Catapano[6], Martina Hrabinová[4,7], Daniel Jun[4], Ľubica Múčková[4,7], Jiří Kuneš[3], Jiří Janoušek[2], Rudolf Andrýs[5], Lucie Nováková[6], Rozálie Peřinová[1], Negar Maafi[1], Ondřej Soukup[4,7], Jan Korábečný[4,7,*], Lucie Cahlíková[1,*]

1 ADINACO Research Group, Department of Pharmaceutical Botany, Faculty of Pharmacy, Charles University, Heyrovskeho 1203, Hradec Kralove 500 05, Czech Republic; almamuna@faf.cuni.cz (A.A.M.); pidany@faf.cuni.cz (F.P.); perinovr@faf.cuni.cz (R.P.); negarm@faf.cuni.cz (N.M.)
2 Department of Pharmacognosy, Faculty of Pharmacy, Charles University, Heyrovskeho 1203, 500 05 Hradec Kralove, Czech Republic; hulcovd@faf.cuni.cz (D.H.); janousj2@faf.cuni.cz (J.J.)
3 Department of Bioorganic and Organic Chemistry, Faculty of Pharmacy, Charles University, Heyrovskeho 1203, 500 05 Hradec Kralove, Czech Republic; marikoj2@faf.cuni.cz (J.M.); kunes@faf.cuni.cz (J.K.)
4 Department of Toxicology and Military Pharmacy, Trebesska 1575, 500 05 Hradec Kralove, Czech Republic; kucera-t@email.cz (T.K.); martina.hrabinova@unob.cz (M.H.); daniel.jun@unob.cz (D.J.); lubica.muckova@unob.cz (L.M)
5 Department of Chemistry, Faculty of Science, University of Hradec Králové, Rokitanského 62, 50003 Hradec Králové, Czech Republic; monika.schmidt@uhk.cz (M.S.); rudolf.andrys@faf.cuni.cz (R.A.)
6 Department of Analytical Chemistry, Faculty of Pharmacy, Charles University, Heyrovskeho 1203, 500 05 Hradec Kralove, Czech Republic; catapanm@faf.cuni.cz (M.C.C.); novakoval@faf.cuni.cz (L.N.)
7 Biomedical Research Centre, University Hospital Hradec Kralove, Sokolska 581, 500 05 Hradec Kralove, Czech Republic; ondrej.soukup@fnhk.cz (O.S.)

*Correspondence: cahlikova@faf.cuni.cz (L.C.), ORCID ID: 0000-0002-1555-8870, Tel. +420 495 067 311; jan.korabecny@fnhk.cz (J.Ko.), ORCID ID: 0000-0001-6977-7596, Tel.: + 420 495 833 447

Table of contents

ESI-HRMS, ^{1}H NMR and ^{13}C NMR spectra of new compounds 4

Figure S1. HRMS of N-allyl-N-(4-benzyloxy-3-methoxybenzyl)-2-(4-hydroxyphenyl)ethan-1-amine (3) 4

Figure S2. ^{1}H NMR spectrum of N-allyl-N-(4-benzyloxy-3-methoxybenzyl)-2-(4-hydroxyphenyl)ethan-1-amine (3) in $CDCl_3$ 4

Figure S3. ^{13}C NMR spectrum of N-allyl-N-(4-benzyloxy-3-methoxybenzyl)-2-(4-hydroxyphenyl)ethan-1-amine (3) in $CDCl_3$.... 5

Figure S4. HRMS of *N*-allyl-*N*-(3-benzyloxy-4-methoxybenzyl)-2-(4-hydroxyphenyl)ethan-1-amine (4).... 5

Figure S5. ^{1}H NMR spectrum *N*-allyl-*N*-(3-benzyloxy-4-methoxybenzyl)-2-(4-hydroxyphenyl)ethan-1-amine (4) in $CDCl_3$ 6

Figure S6. ^{13}C NMR spectrum of *N*-allyl-*N*-(3-benzyloxy-4-methoxybenzyl)-2-(4-hydroxyphenyl)ethan-1-amine (4) in $CDCl_3$ 6

Figure S7. HRMS of *N*-allyl-*N*-(4-benzyloxy-3-methoxybenzyl)-2-(4-allyloxyphenyl)ethan-1-amine (5).... 7

Figure S8. ^{1}H NMR spectrum of *N*-allyl-*N*-(4-benzyloxy-3-methoxybenzyl)-2-(4-allyloxyphenyl)ethan-1-amine (5) in $CDCl_3$.... 7

Figure S9. ^{13}C NMR spectrum of of *N*-allyl-*N*-(4-benzyloxy-3-methoxybenzyl)-2-(4-allyloxyphenyl)ethan-1-amine (5) in $CDCl_3$. 8

Figure S10. HRMS of *N*-allyl-*N*-(3-benzyloxy-4-methoxybenzyl)-2-(4-allyloxyphenyl)ethan-1-amine (6).... 8

Figure S11. ^{1}H NMR spectrum of *N*-allyl-*N*-(3-benzyloxy-4-methoxybenzyl)-2-(4-allyloxyphenyl)ethan-1-amine (6) in $CDCl_3$.... 9

Figure S12. ^{13}C NMR spectrum of *N*-allyl-*N*-(3-benzyloxy-4-methoxybenzyl)-2-(4-allyloxyphenyl)ethan-1-amine (6) in $CDCl_3$.... 9

Figure S13. HRMS of *N*-(4-benzyloxy-3-methoxybenzyl)-2-phenylethan-1-amine (7).... 9

Figure S14. ^{1}H NMR spectrum of *N*-(4-benzyloxy-3-methoxybenzyl)-2-phenylethan-1-amine (7) in $CDCl_3$.... 10

Figure S15. ^{13}C NMR spectrum of *N*-(4-benzyloxy-3-methoxybenzyl)-2-phenylethan-1-amine (7) in $CDCl_3$ 10

Figure S16. HRMS of *N*-(3-benzyloxy-4-methoxybenzyl)-2-phenylethan-1-amine (8).... 11

Figure S17. ^{1}H NMR spectrum of *N*-(3-benzyloxy-4-methoxybenzyl)-2-phenylethan-1-amine (8) in $CDCl_3$.... 11

Figure S18. ^{13}C NMR spectrum of *N*-(3-benzyloxy-4-methoxybenzyl)-2-phenylethan-1-amine (8) in $CDCl_3$.... 12

Figure S19. HRMS of *N*-allyl-*N*-(4-benzyloxy-3-methoxybenzyl)-2-phenylethan-1-amine (9) 12

Figure S20. ^{1}H NMR spectrum of *N*-allyl-*N*-(4-benzyloxy-3-methoxybenzyl)-2-phenylethan-1-amine (9) in $CDCl_3$ 13

Figure S21. ^{13}C NMR spectrum of *N*-allyl-*N*-(4-benzyloxy-3-methoxybenzyl)-2-phenylethan-1-amine (9) in $CDCl_3$.... 13

Figure S22. HRMS of *N*-allyl-*N*-(3-benzyloxy-4-methoxybenzyl)-2-phenylethan-1-amine (10) 14

Figure S23. ^{1}H NMR spectrum of *N*-allyl-*N*-(3-benzyloxy-4-methoxybenzyl)-2-phenylethan-1-amine (10) in $CDCl_3$.... 14

Figure S24. ^{13}C NMR spectrum of *N*-allyl-*N*-(3-benzyloxy-4-methoxybenzyl)-2-phenylethan-1-amine (10) in $CDCl_3$.... 15

Figure S25. HRMS of *N*-(4-benzyloxy-3-methoxybenzyl)-2-(4-methoxyphenyl)ethan-1-amine (11).... 15

Figure S26. ^{1}H NMR spectrum of *N*-(4-benzyloxy-3-methoxybenzyl)-2-(4-methoxyphenyl)ethan-1-amine (11) in $CDCl_3$.... 16

Figure S27. ^{13}C NMR spectrum of *N*-(4-benzyloxy-3-methoxybenzyl)-2-(4-methoxyphenyl)ethan-1-amine (11) in $CDCl_3$.... 16

Figure S28. HRMS of *N*-(3-benzyloxy-4-methoxybenzyl)-2-(4-methoxyphenyl)ethan-1-amine (12).... 17

Figure S29. ^{1}H NMR spectrum of *N*-(3-benzyloxy-4-methoxybenzyl)-2-(4-methoxyphenyl)ethan-1-amine (12) in $CDCl_3$.... 17

Figure S30. ^{13}C NMR spectrum of *N*-(3-benzyloxy-4-methoxybenzyl)-2-(4-methoxyphenyl)ethan-1-amine (12) in $CDCl_3$.... 18

Figure S31. HRMS of *N*-allyl-*N*-(4-benzyloxy-3-methoxybenzyl)-2-(4-methoxyphenyl)ethan-1-amine (13) 18

Figure S32. ^{1}H NMR spectrum of *N*-allyl-*N*-(4-benzyloxy-3-methoxybenzyl)-2-(4-methoxyphenyl)ethan-1-amine (13) in $CDCl_3$ 19

Figure S33. ^{13}C NMR spectrum of *N*-allyl-*N*-(4-benzyloxy-3-methoxybenzyl)-2-(4-methoxyphenyl)ethan-1-amine (13) in $CDCl_3$19

Figure S34. HRMS of *N*-allyl-*N*-(3-benzyloxy-4-methoxybenzyl)-2-(4-methoxyphenyl)ethan-1-amine (14) 20

Figure S35. ^{1}H NMR spectrum of *N*-allyl-*N*-(3-benzyloxy-4-methoxybenzyl)-2-(4-methoxyphenyl)ethan-1-amine (14) in $CDCl_3$ 20

Figure S36. ^{13}C NMR spectrum of *N*-allyl-*N*-(3-benzyloxy-4-methoxybenzyl)-2-(4-methoxyphenyl)ethan-1-amine (14) in $CDCl_3$21

Figure S37. HRMS of *N*-(3-ethoxy-4-methoxybenzyl)-2-(4-hydroxyphenyl)ethan-1-amine (15) 21

Figure S38. ^{1}H NMR spectrum of *N*-(3-ethoxy-4-methoxybenzyl)-2-(4-hydroxyphenyl)ethan-1-amine (15) in $CDCl_3$ 22

Figure S39. ^{13}C NMR spectrum of *N*-(3-ethoxy-4-methoxybenzyl)-2-(4-hydroxyphenyl)ethan-1-amine (15) in $CDCl_3$ 22

Figure S40. HRMS of *N*-allyl-*N*-(3-ethoxy-4-methoxybenzyl)-2-(4-hydroxyphenyl)ethan-1-amine (16) 23

Figure S41. ^{1}H NMR spectrum of *N*-allyl-*N*-(3-ethoxy-4-methoxybenzyl)-2-(4-hydroxyphenyl)ethan-1-amine (16) in $CDCl_3$ 23
Figure S42. ^{13}C NMR spectrum of *N*-allyl-*N*-(3-ethoxy-4-methoxybenzyl)-2-(4-hydroxyphenyl)ethan-1-amine (16) in $CDCl_3$ 24
Figure S43. HRMS of *N*-allyl-*N*-(3-ethoxy-4-methoxybenzyl)-2-(4-allyloxyphenyl)ethan-1-amine (17) 24
Figure S44. ^{1}H NMR spectrum of *N*-allyl-*N*-(3-ethoxy-4-methoxybenzyl)-2-(4-allyloxyphenyl)ethan-1-amine (17) in $CDCl_3$....... 25
Figure S45. ^{13}C NMR spectrum of *N*-allyl-*N*-(3-ethoxy-4-methoxybenzyl)-2-(4-allyloxyphenyl)ethan-1-amine (17) in $CDCl_3$ 25
Figure S46. HRMS of *N*-(3-ethoxy-4-methoxybenzyl)-2-phenylethan-1-amine (18) 26
Figure S47. ^{1}H NMR spectrum of *N*-(3-ethoxy-4-methoxybenzyl)-2-phenylethan-1-amine (18) in $CDCl_3$........ 26
Figure S48. ^{13}C NMR spectrum of *N*-(3-ethoxy-4-methoxybenzyl)-2-phenylethan-1-amine (18) in $CDCl_3$ 27
Figure S49. HRMS of *N*-allyl-*N*-(3-ethoxy-4-methoxybenzyl)-2-phenylethan-1-amine (19)........ 27
Figure S50. ^{1}H NMR spectrum of *N*-allyl-*N*-(3-ethoxy-4-methoxybenzyl)-2-phenylethan-1-amine (19) in $CDCl_3$........ 28
Figure S51. ^{13}C NMR spectrum of *N*-allyl-*N*-(3-ethoxy-4-methoxybenzyl)-2-phenylethan-1-amine (19) in $CDCl_3$........ 28
Figure S52. HRMS of *N*-benzyl-*N*-(3-ethoxy-4-methoxybenzyl)-2-phenylethan-1-amine (20)........ 29
Figure S53. ^{1}H NMR spectrum of *N*-benzyl-*N*-(3-ethoxy-4-methoxybenzyl)-2-phenylethan-1-amine (20) in $CDCl_3$........ 29
Figure S54. ^{13}C NMR spectrum of *N*-benzyl-*N*-(3-ethoxy-4-methoxybenzyl)-2-phenylethan-1-amine (20) in $CDCl_3$........ 30
Biological assays in detail 31
hAChE and hBuChE Inhibition Assay 31
Kinetic Study of Cholinesterase Inhibition 31
POP inhibition assay 32
MAOs inhibition assay 32

ESI-HRMS, ^{1}H NMR and ^{13}C NMR spectra of new compounds

Figure S1. HRMS of N-allyl-N-(4-benzyloxy-3-methoxybenzyl)-2-(4-hydroxyphenyl)ethan-1-amine (**3**)

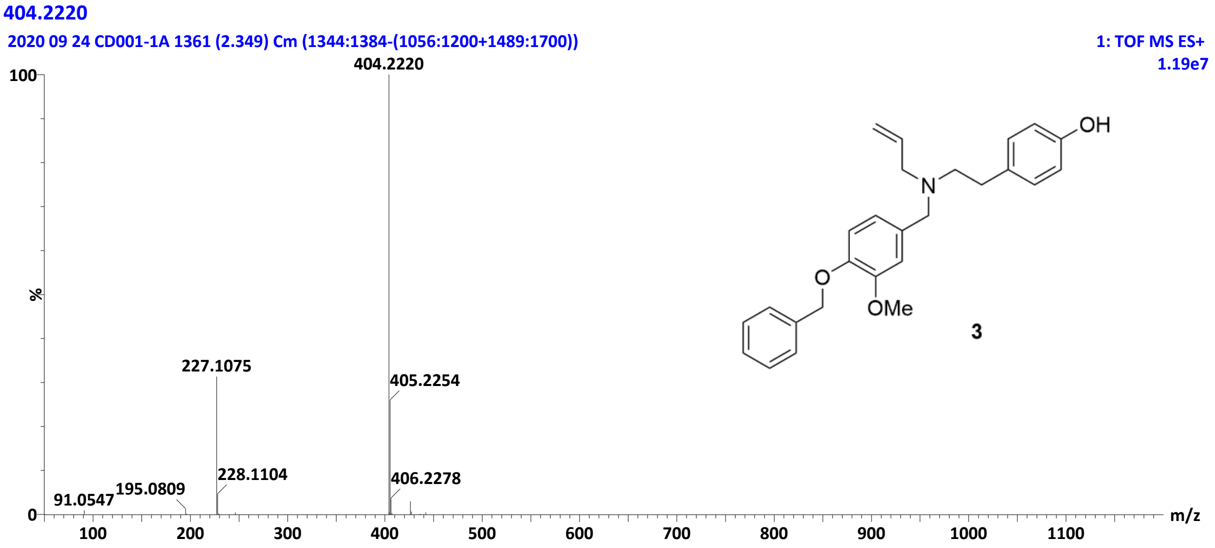

Figure S2. ^{1}H NMR spectrum of N-allyl-N-(4-benzyloxy-3-methoxybenzyl)-2-(4-hydroxyphenyl)ethan-1-amine (3) in $CDCl_3$

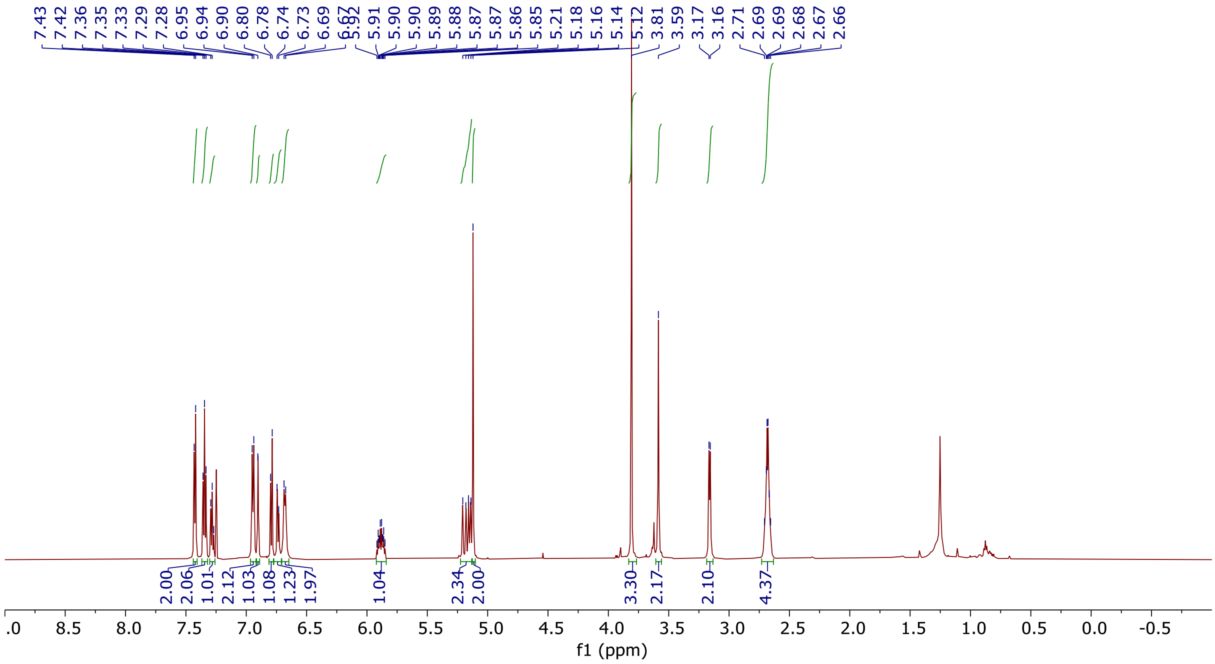

Figure S3. ^{13}C NMR spectrum of N-allyl-N-(4-benzyloxy-3-methoxybenzyl)-2-(4-hydroxyphenyl)ethan-1-amine (3) in $CDCl_3$

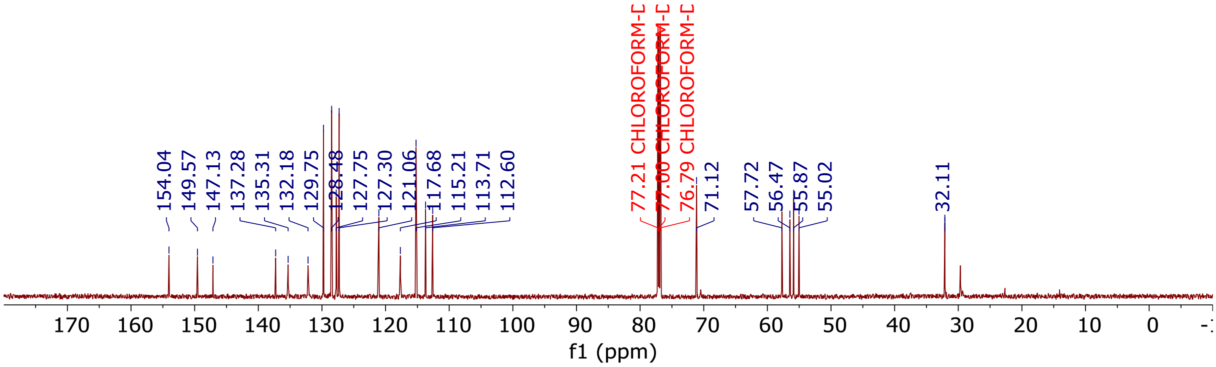

Figure S4. HRMS of *N*-allyl-*N*-(3-benzyloxy-4-methoxybenzyl)-2-(4-hydroxyphenyl)ethan-1-amine (**4**)

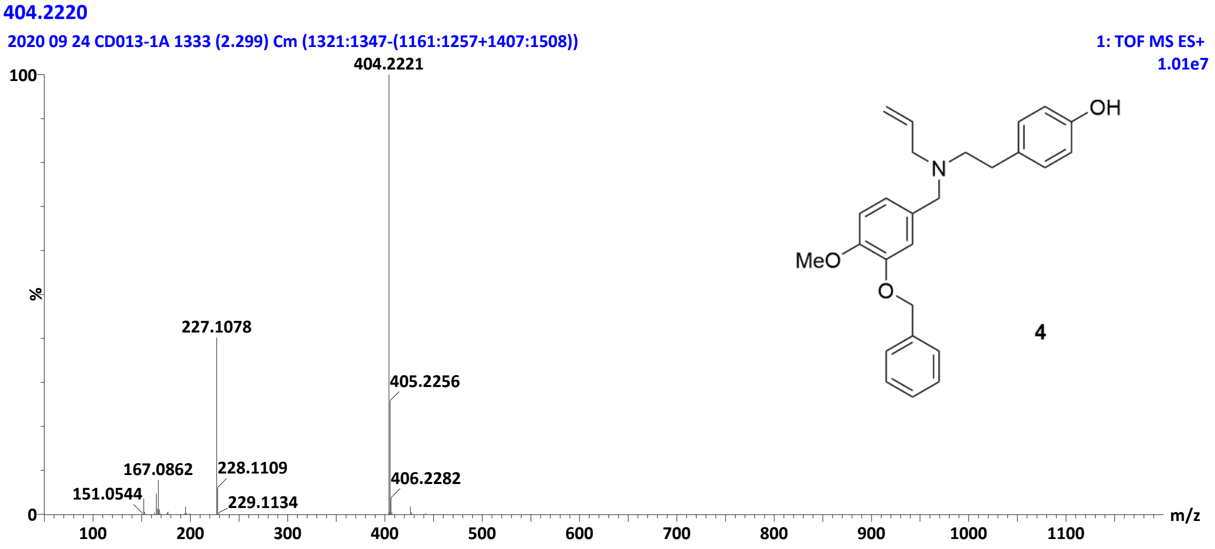

Figure S5. ^{1}H NMR spectrum *N*-allyl-*N*-(3-benzyloxy-4-methoxybenzyl)-2-(4-hydroxyphenyl)ethan-1-amine (4) in $CDCl_3$

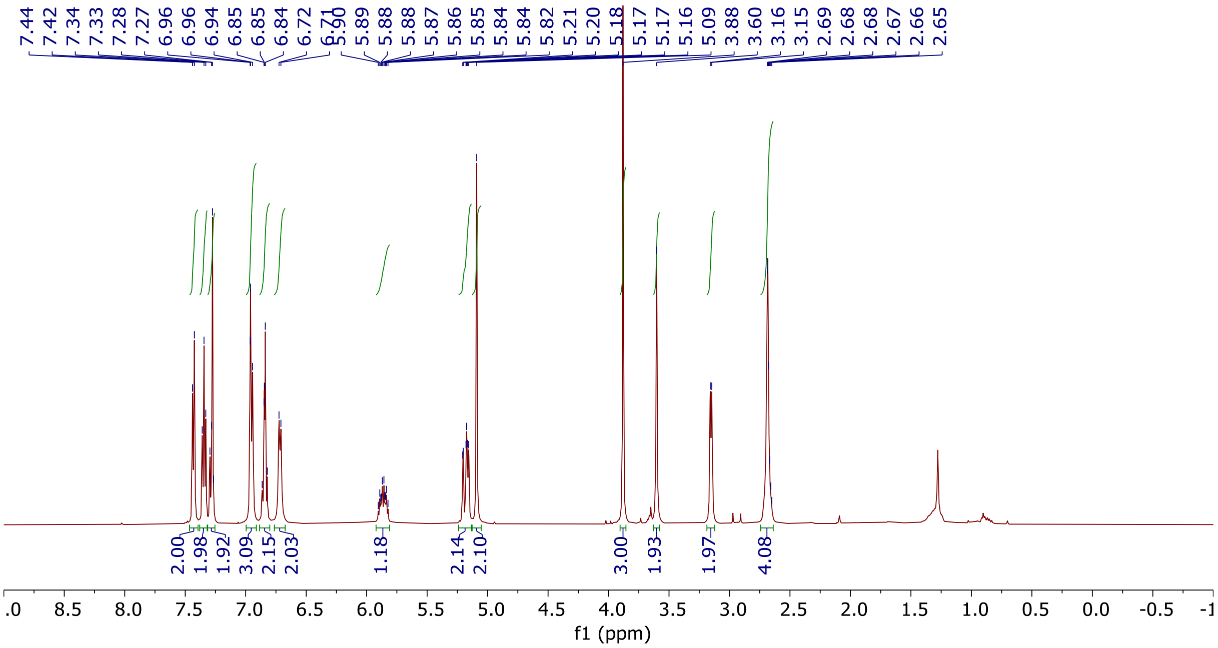

Figure S6. ^{13}C NMR spectrum of *N*-allyl-*N*-(3-benzyloxy-4-methoxybenzyl)-2-(4-hydroxyphenyl)ethan-1-amine (4) in $CDCl_3$

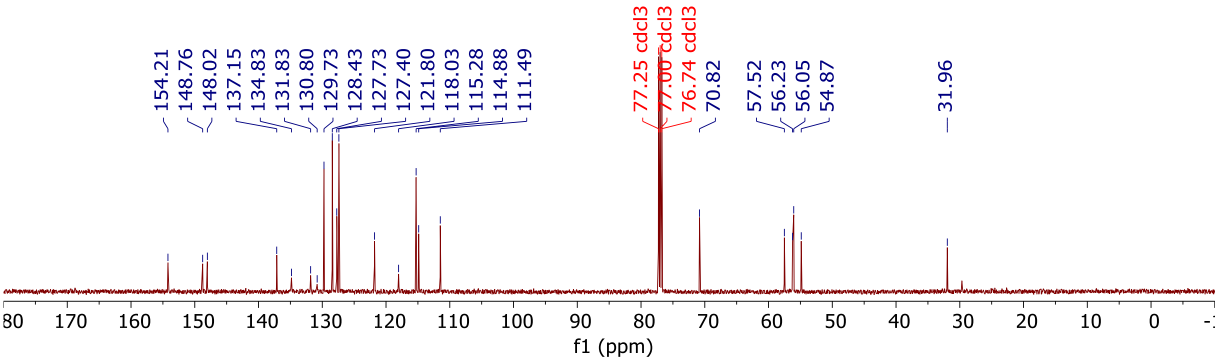

Figure S7. HRMS of *N*-allyl-*N*-(4-benzyloxy-3-methoxybenzyl)-2-(4-allyloxyphenyl)ethan-1-amine (**5**)

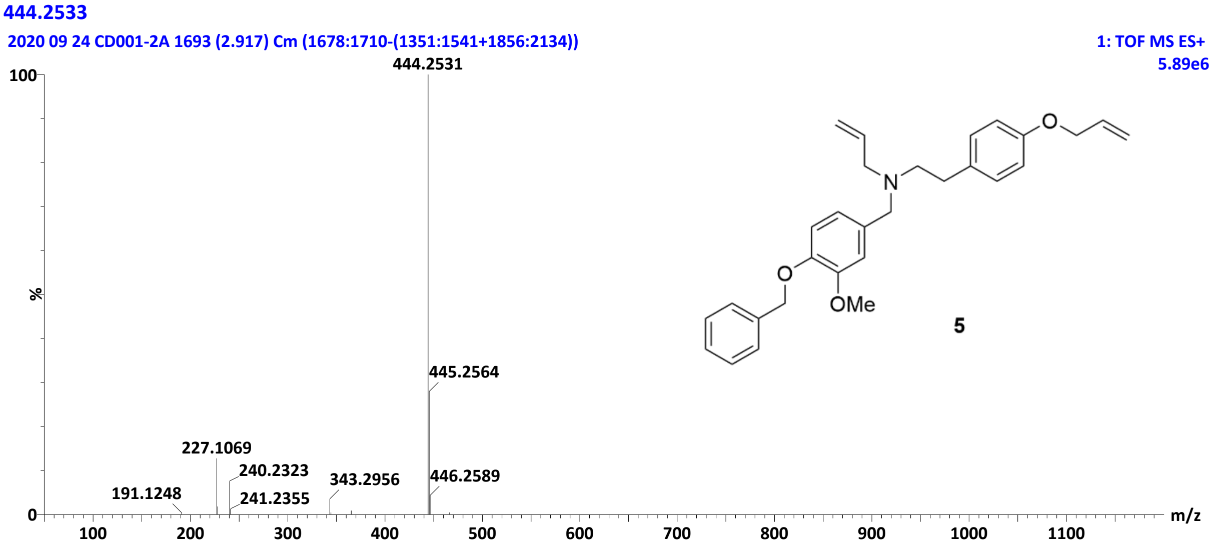

Figure S8. ^{1}H NMR spectrum of *N*-allyl-*N*-(4-benzyloxy-3-methoxybenzyl)-2-(4-allyloxyphenyl)ethan-1-amine (5) in $CDCl_3$

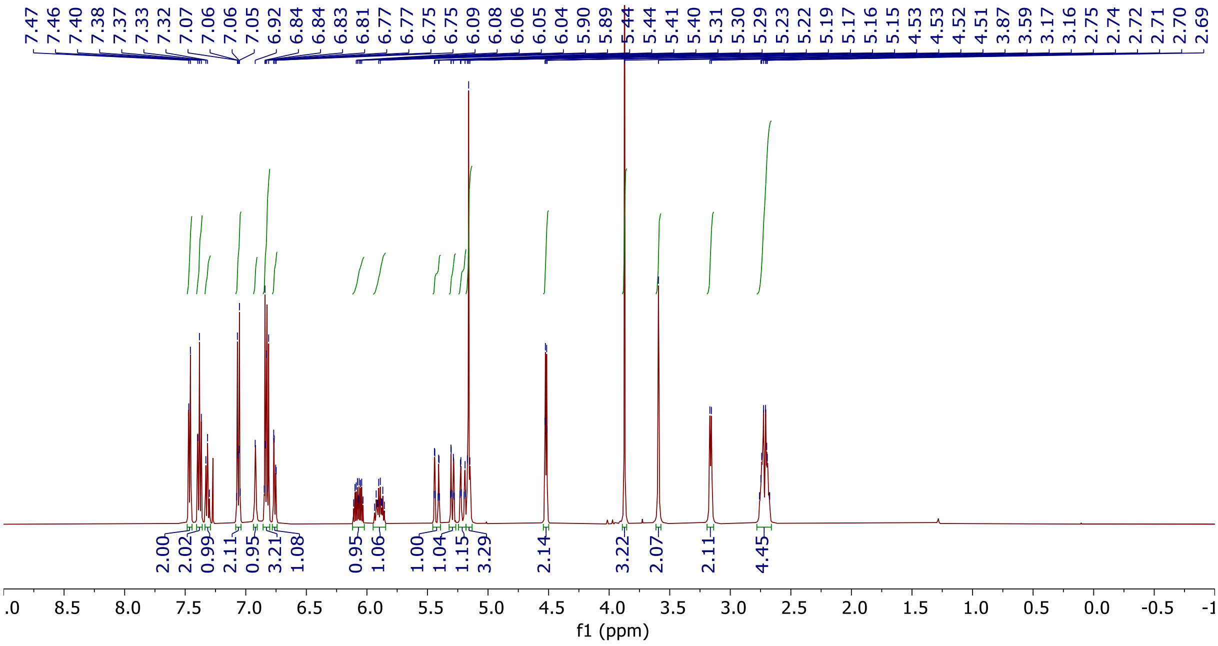

Figure S9. ^{13}C NMR spectrum of of *N*-allyl-*N*-(4-benzyloxy-3-methoxybenzyl)-2-(4-allyloxyphenyl)ethan-1-amine (5) in $CDCl_3$

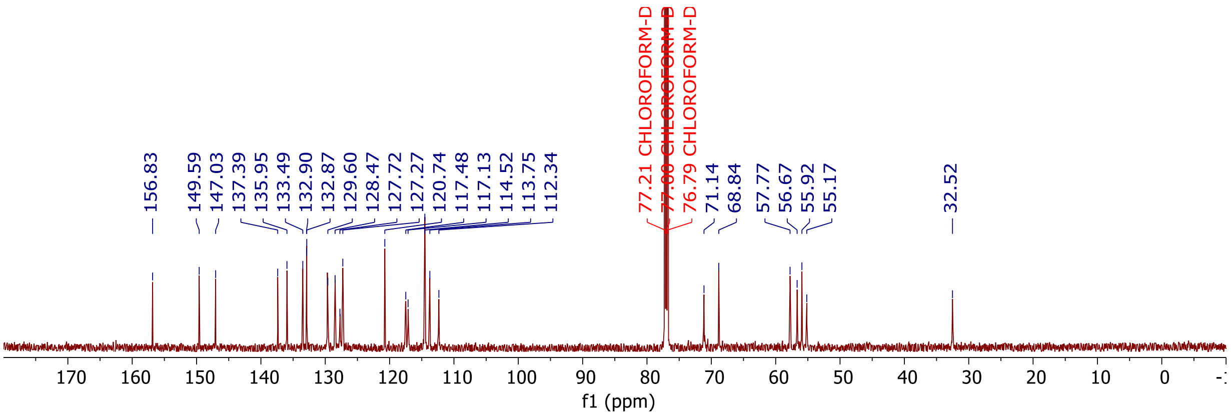

Figure S10. HRMS of *N*-allyl-*N*-(3-benzyloxy-4-methoxybenzyl)-2-(4-allyloxyphenyl)ethan-1-amine (**6**)

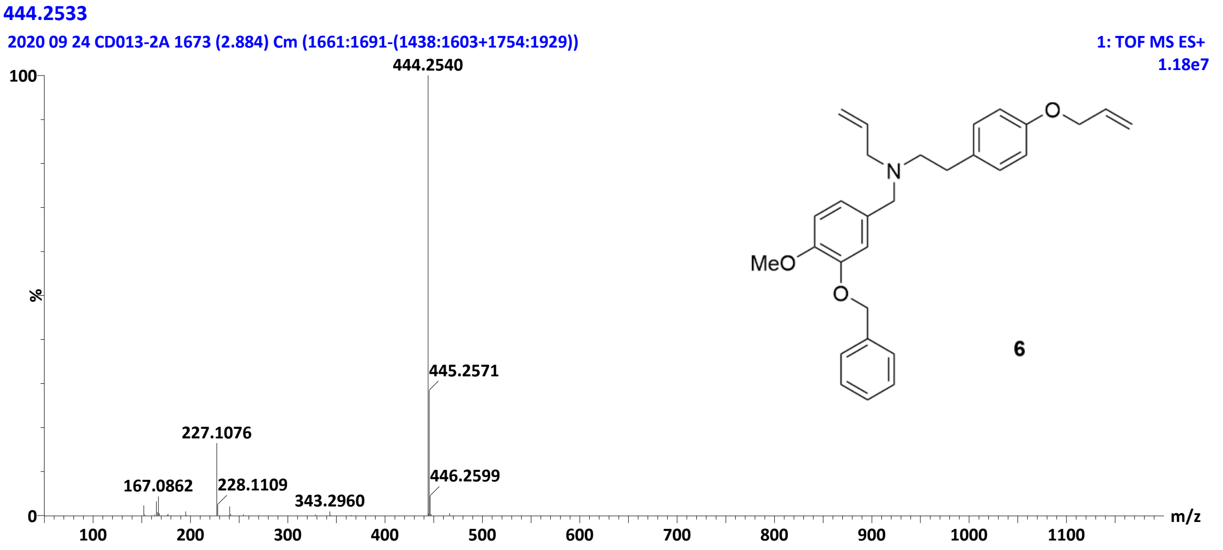

Figure S11. ^{1}H NMR spectrum of *N*-allyl-*N*-(3-benzyloxy-4-methoxybenzyl)-2-(4-allyloxyphenyl)ethan-1-amine (6) in $CDCl_3$

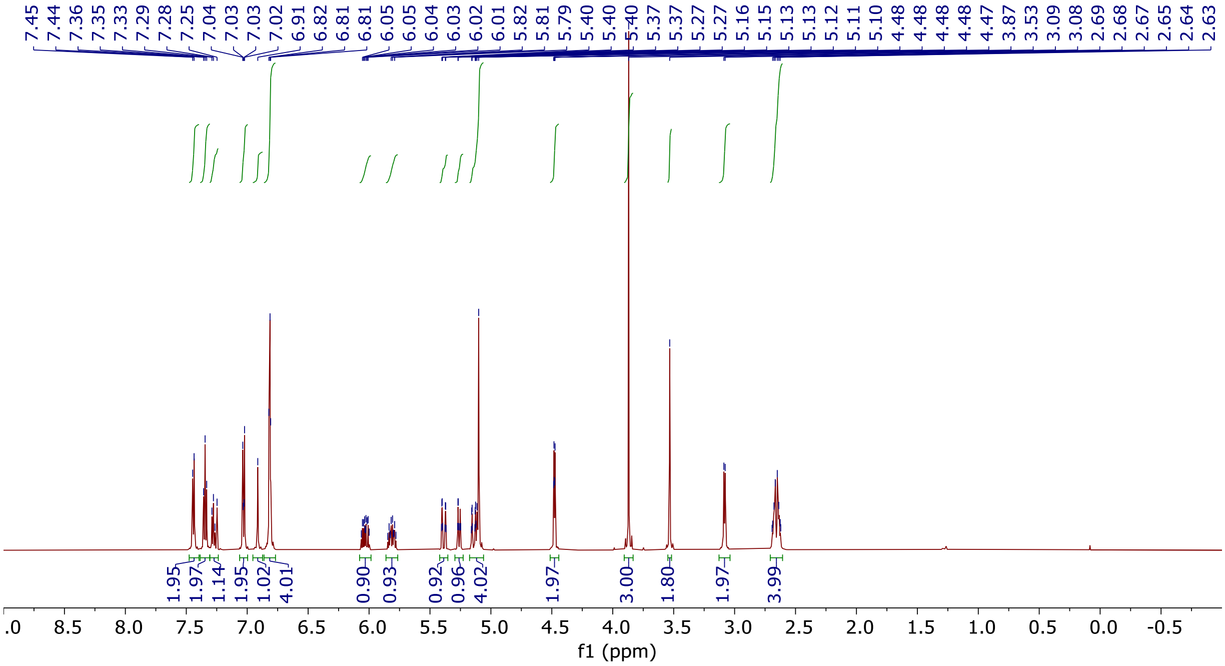

Figure S12. ^{13}C NMR spectrum of *N*-allyl-*N*-(3-benzyloxy-4-methoxybenzyl)-2-(4-allyloxyphenyl)ethan-1-amine (6) in $CDCl_3$

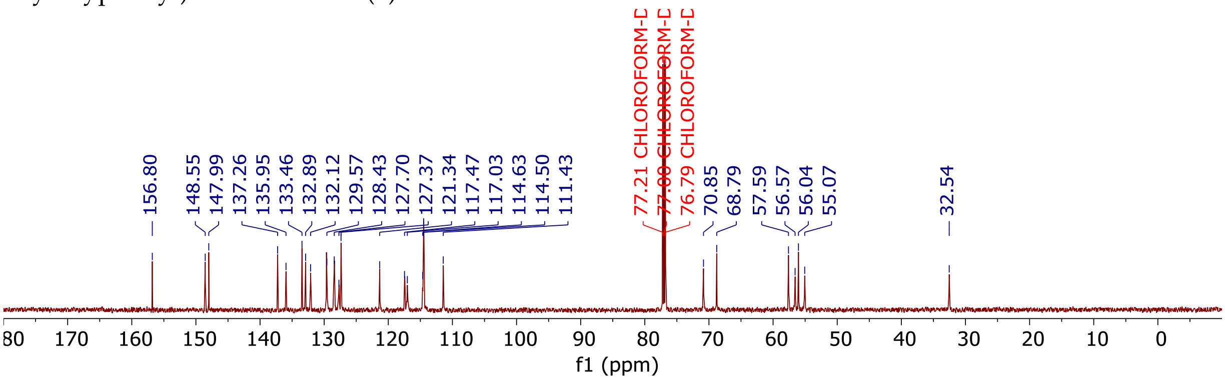

Figure S13. HRMS of *N*-(4-benzyloxy-3-methoxybenzyl)-2-phenylethan-1-amine (**7**)

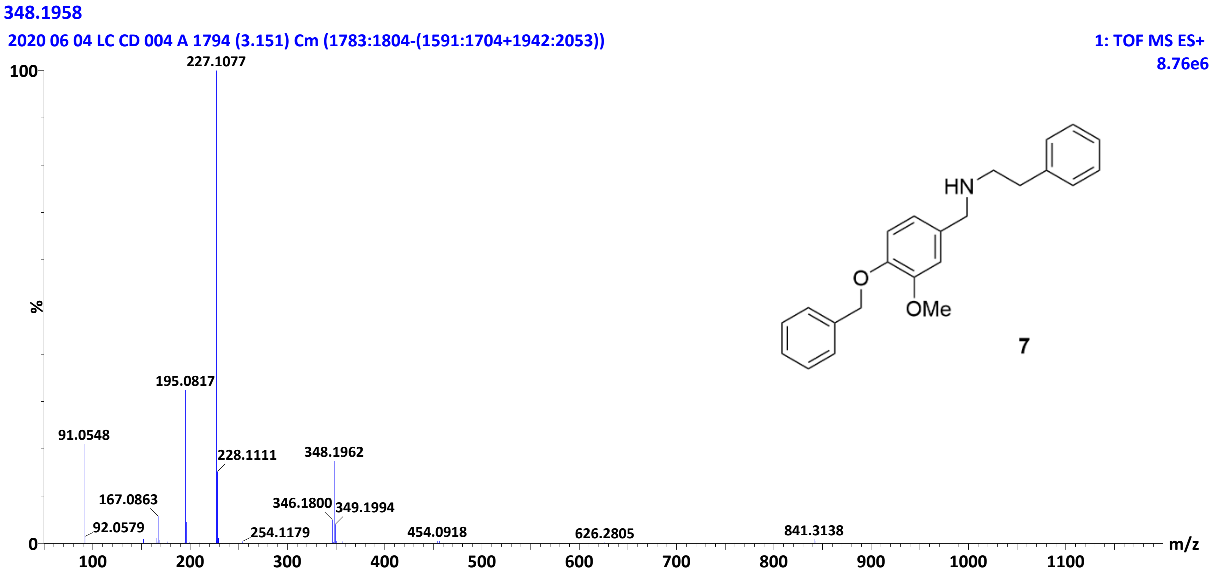

Figure S14. ^{1}H NMR spectrum of *N*-(4-benzyloxy-3-methoxybenzyl)-2-phenylethan-1-amine (7) in $CDCl_3$

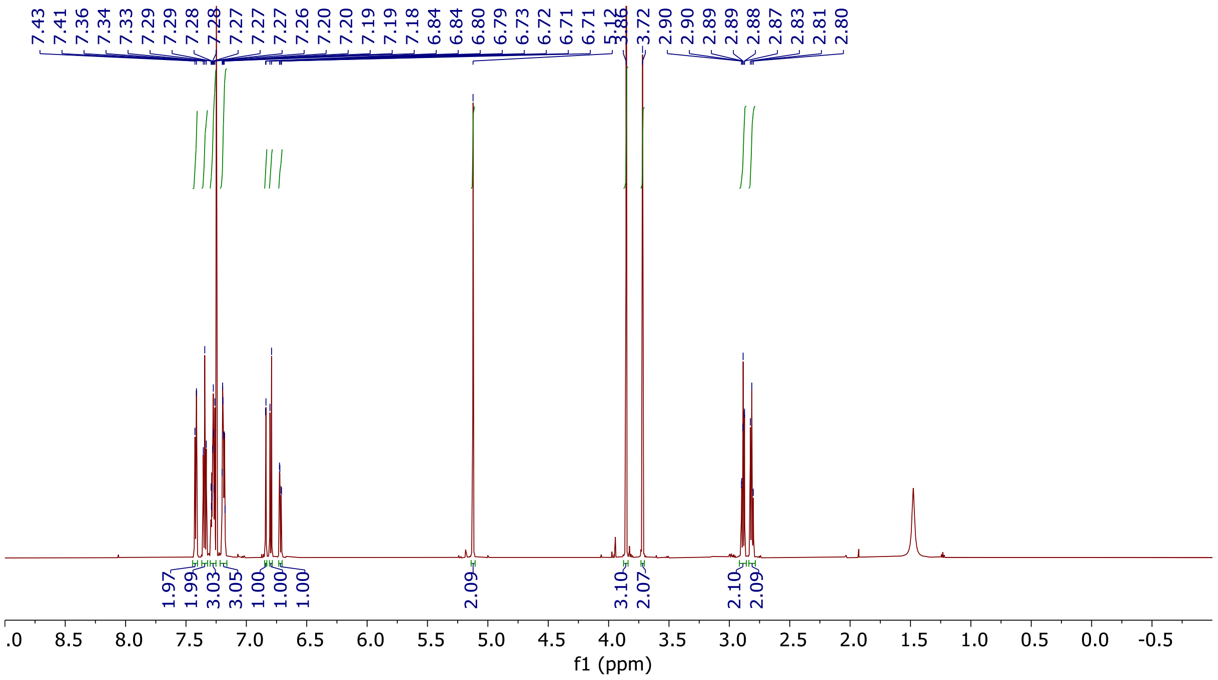

Figure S15. ^{13}C NMR spectrum of *N*-(4-benzyloxy-3-methoxybenzyl)-2-phenylethan-1-amine (7) in $CDCl_3$

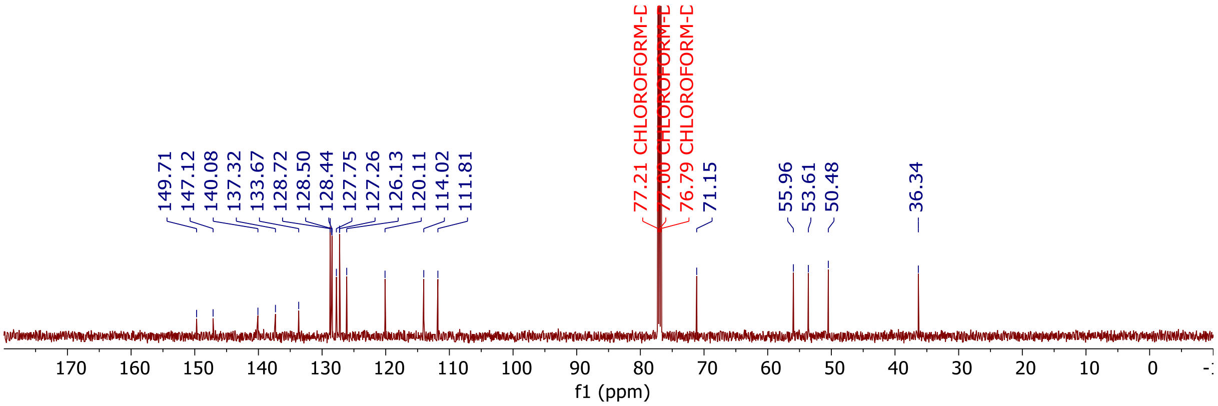

Figure S16. HRMS of *N*-(3-benzyloxy-4-methoxybenzyl)-2-phenylethan-1-amine (**8**)

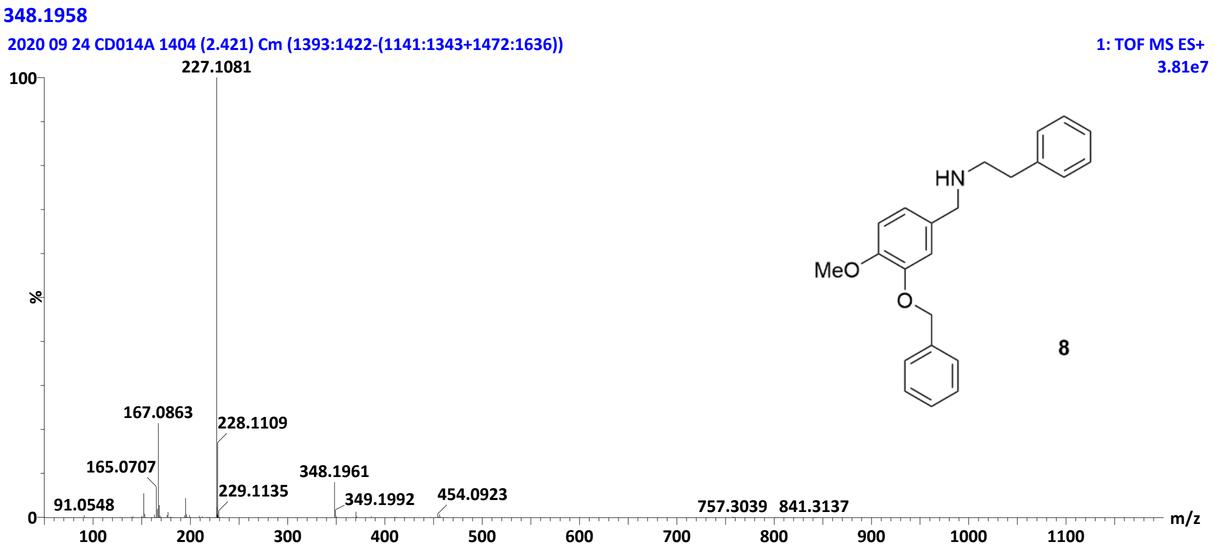

Figure S17. ¹H NMR spectrum of *N*-(3-benzyloxy-4-methoxybenzyl)-2-phenylethan-1-amine (8) in $CDCl_3$

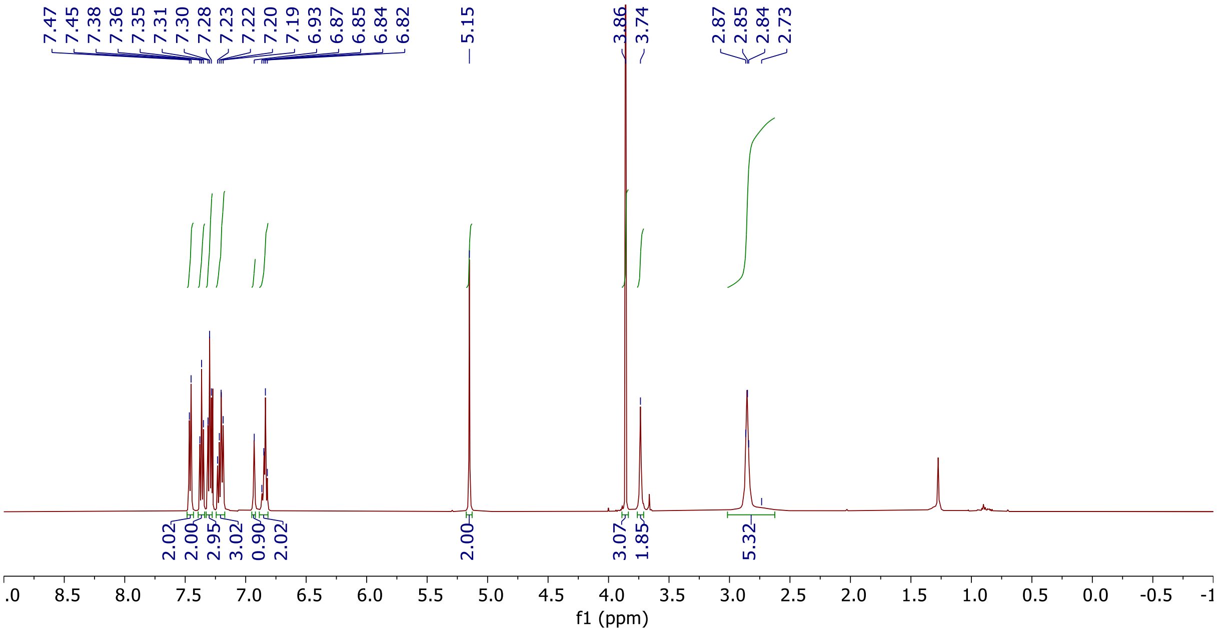

Figure S18. ^{13}C NMR spectrum of *N*-(3-benzyloxy-4-methoxybenzyl)-2-phenylethan-1-amine (8) in $CDCl_3$

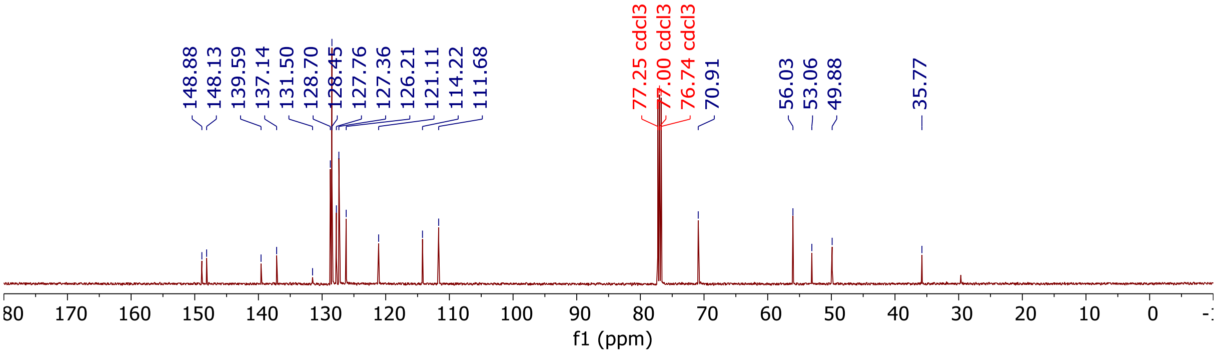

Figure S19. HRMS of *N*-allyl-*N*-(4-benzyloxy-3-methoxybenzyl)-2-phenylethan-1-amine (**9**)

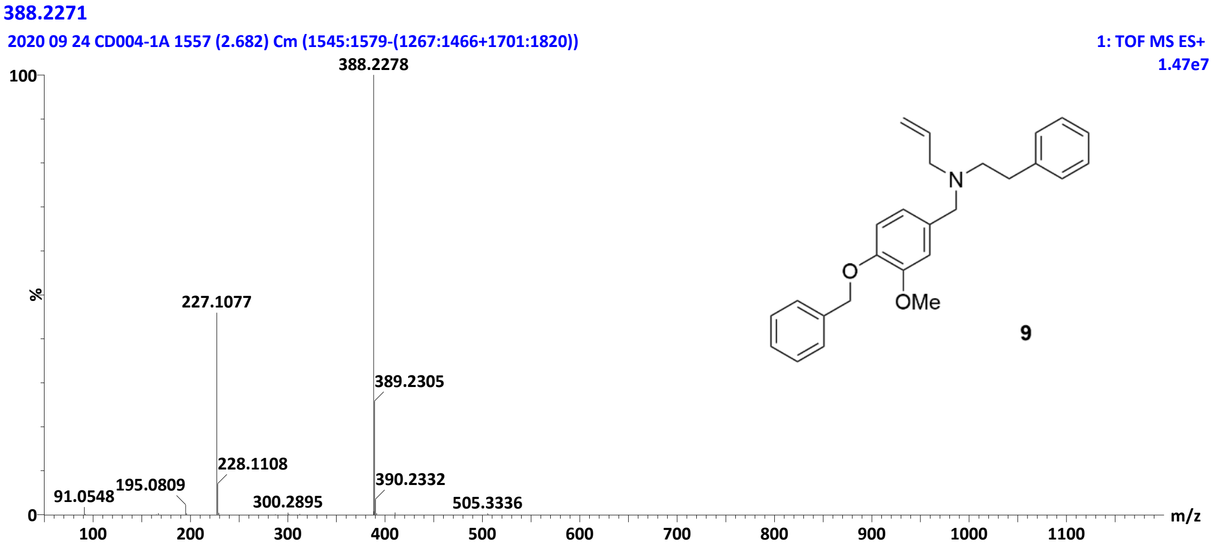

Figure S20. ^{1}H NMR spectrum of *N*-allyl-*N*-(4-benzyloxy-3-methoxybenzyl)-2-phenylethan-1-amine (9) in $CDCl_3$

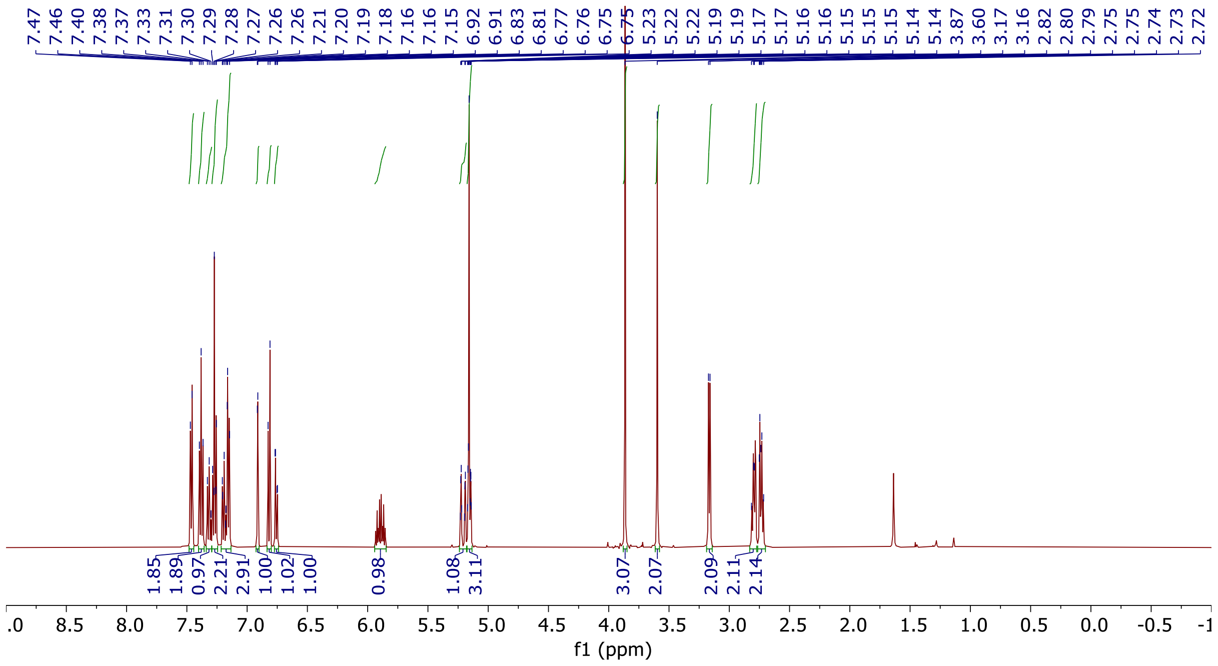

Figure S21. ^{13}C NMR spectrum of *N*-allyl-*N*-(4-benzyloxy-3-methoxybenzyl)-2-phenylethan-1-amine (9) in $CDCl_3$

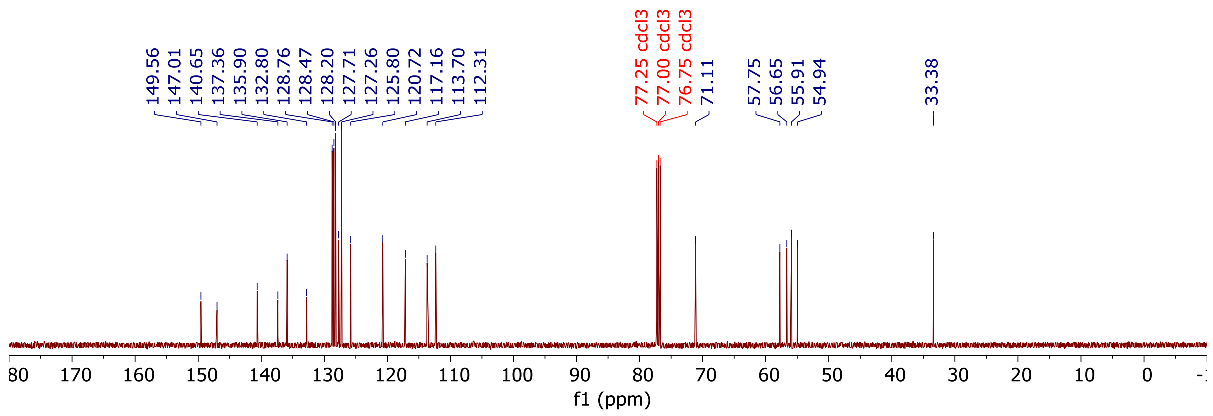

Figure S22. HRMS of *N*-allyl-*N*-(3-benzyloxy-4-methoxybenzyl)-2-phenylethan-1-amine (**10**)

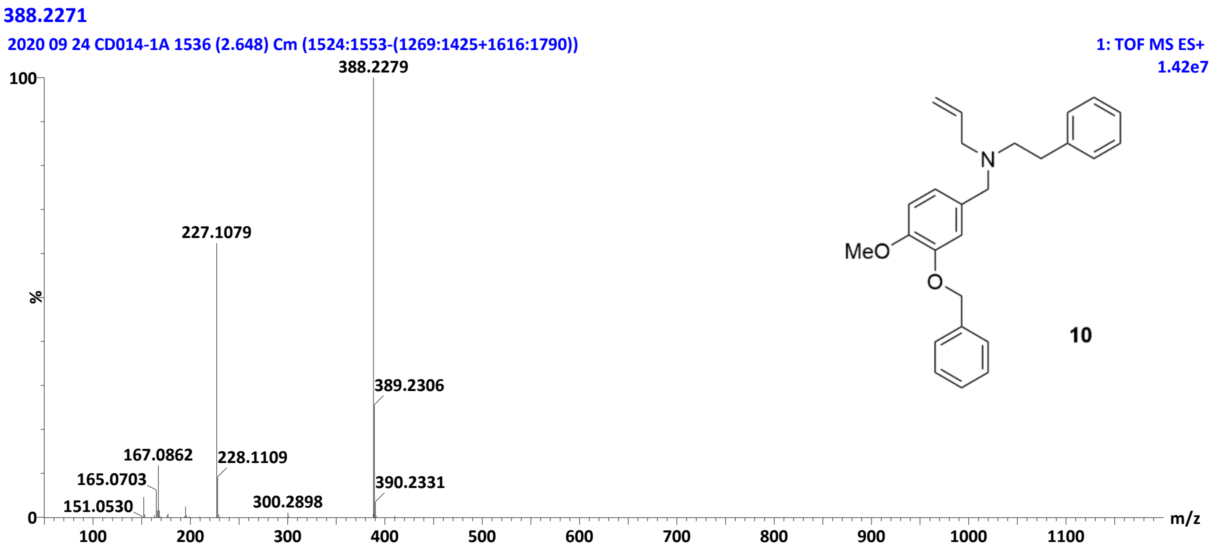

Figure S23. ^{1}H NMR spectrum of *N*-allyl-*N*-(3-benzyloxy-4-methoxybenzyl)-2-phenylethan-1-amine (10) in $CDCl_3$

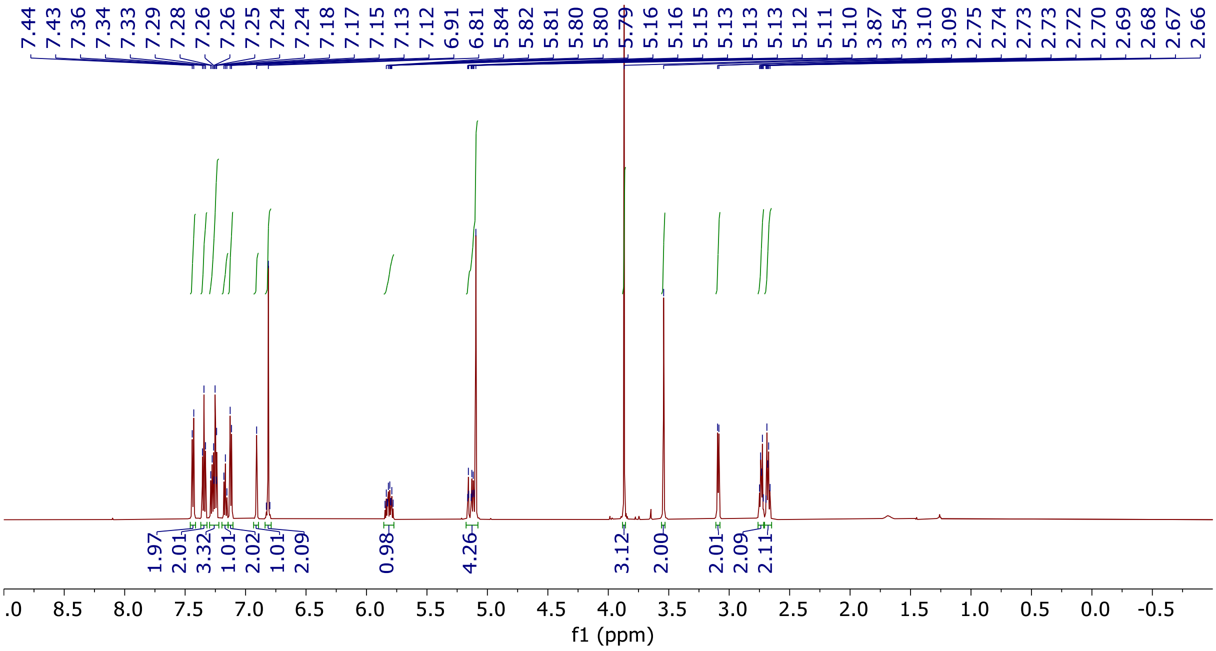

Figure S24. ^{13}C NMR spectrum of *N*-allyl-*N*-(3-benzyloxy-4-methoxybenzyl)-2-phenylethan-1-amine (10) in $CDCl_3$

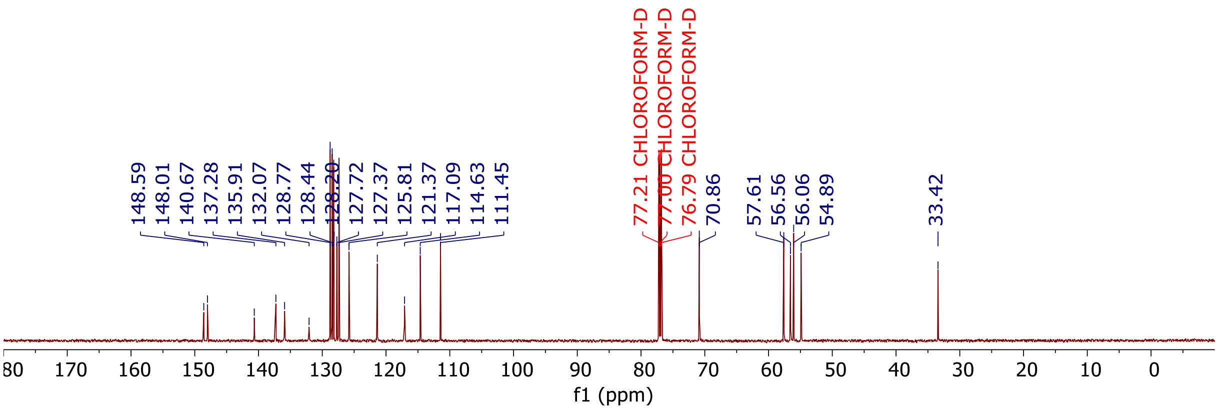

Figure S25. HRMS of *N*-(4-benzyloxy-3-methoxybenzyl)-2-(4-methoxyphenyl)ethan-1-amine (**11**)

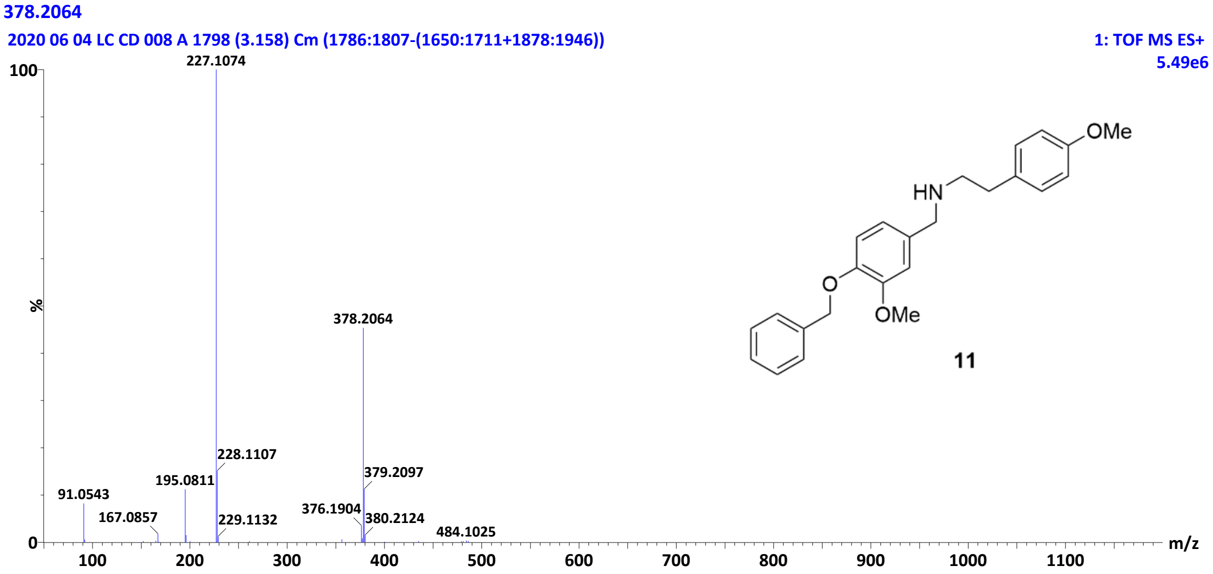

Figure S26. ^{1}H NMR spectrum of *N*-(4-benzyloxy-3-methoxybenzyl)-2-(4-methoxyphenyl)ethan-1-amine (11) in $CDCl_3$

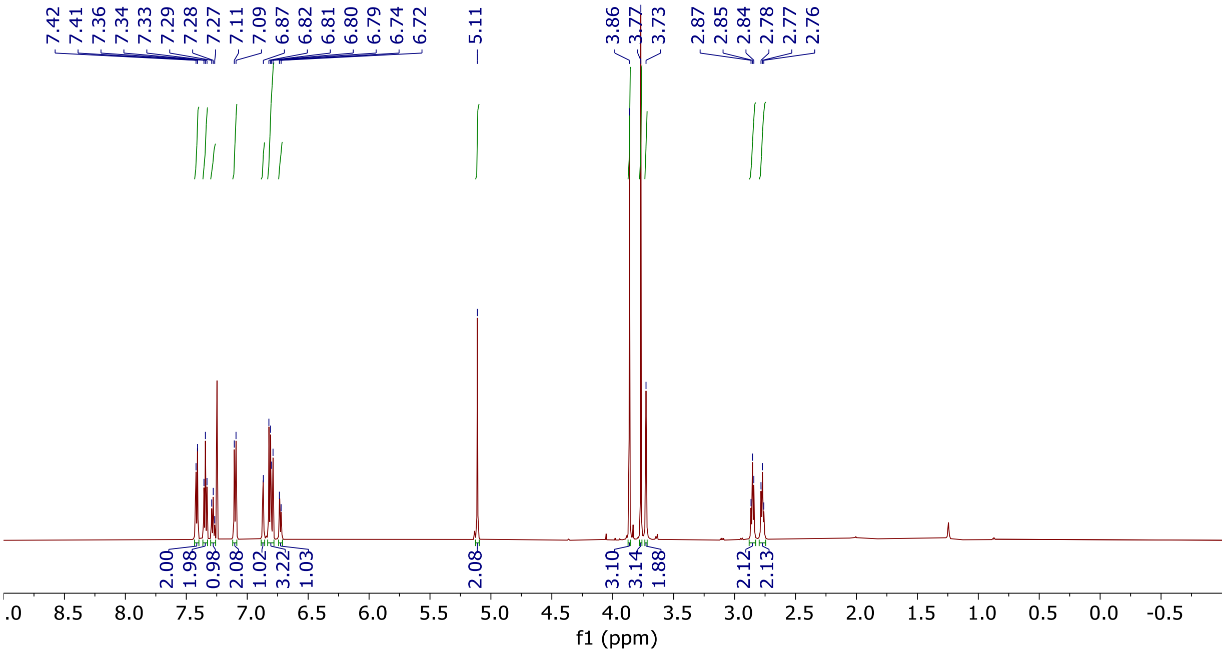

Figure S27. ^{13}C NMR spectrum of *N*-(4-benzyloxy-3-methoxybenzyl)-2-(4-methoxyphenyl)ethan-1-amine (11) in $CDCl_3$

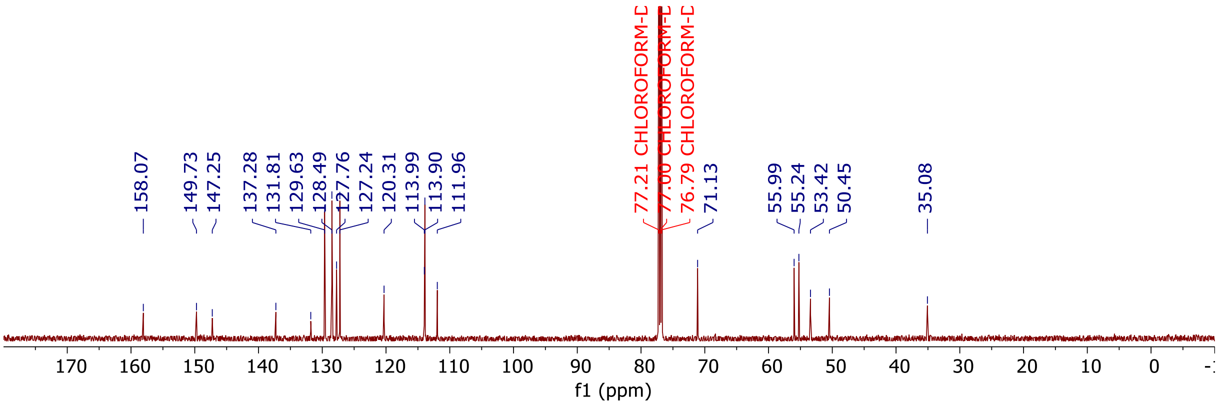

Figure S28. HRMS of *N*-(3-benzyloxy-4-methoxybenzyl)-2-(4-methoxyphenyl)ethan-1-amine (**12**)

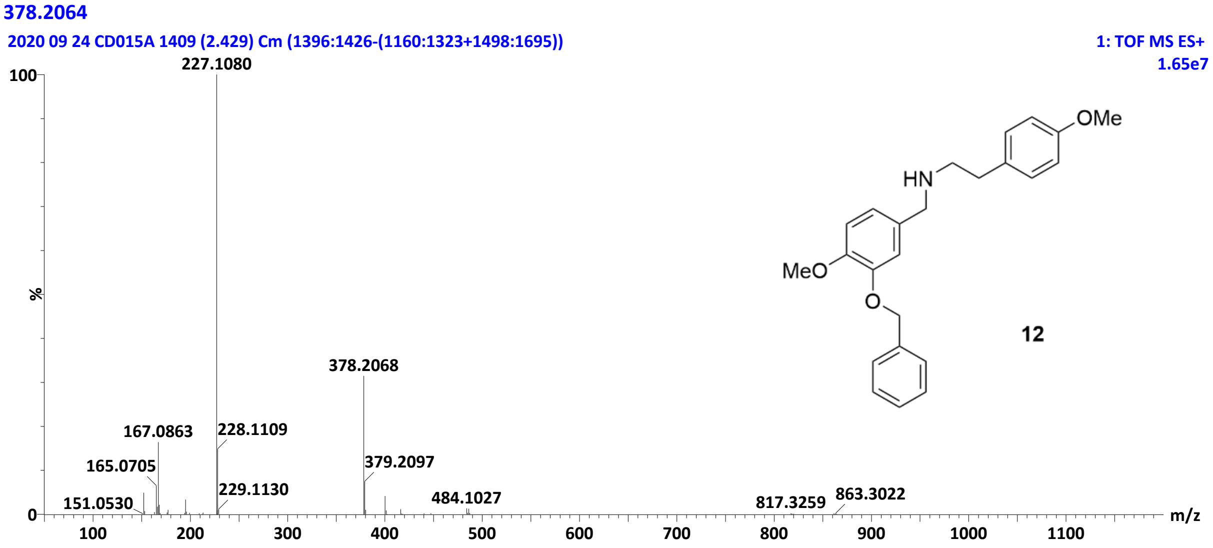

Figure S29. ^{1}H NMR spectrum of *N*-(3-benzyloxy-4-methoxybenzyl)-2-(4-methoxyphenyl)ethan-1-amine (12) in $CDCl_3$

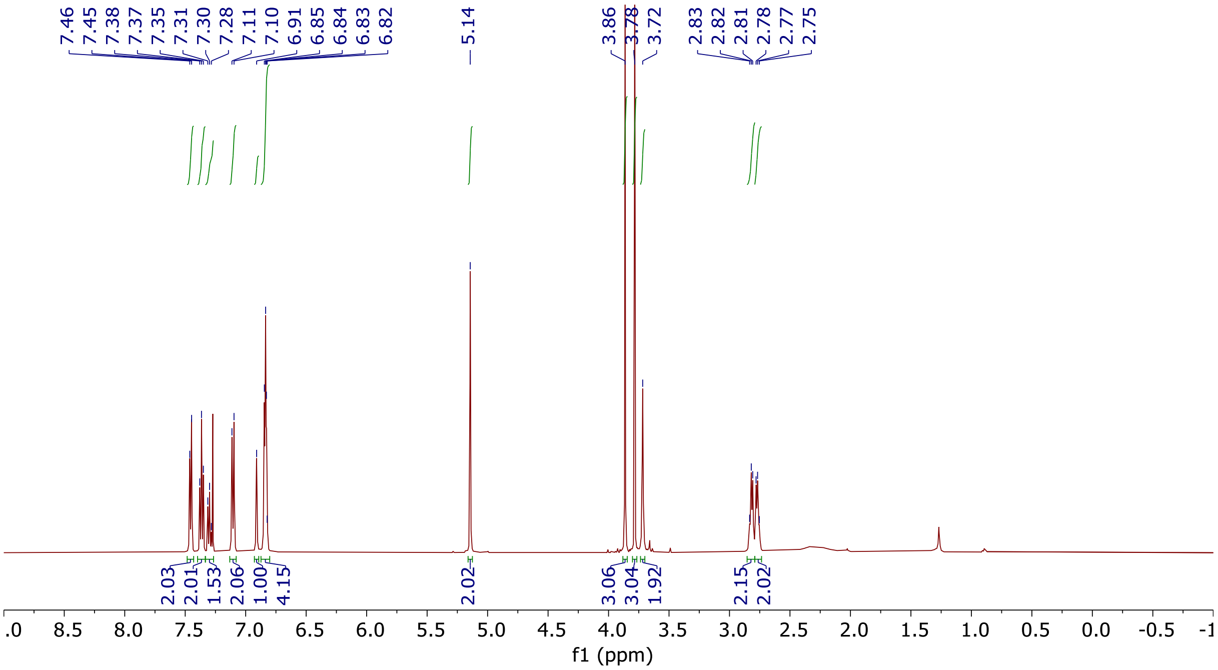

Figure S30. ^{13}C NMR spectrum of *N*-(3-benzyloxy-4-methoxybenzyl)-2-(4-methoxyphenyl)ethan-1-amine (12) in $CDCl_3$

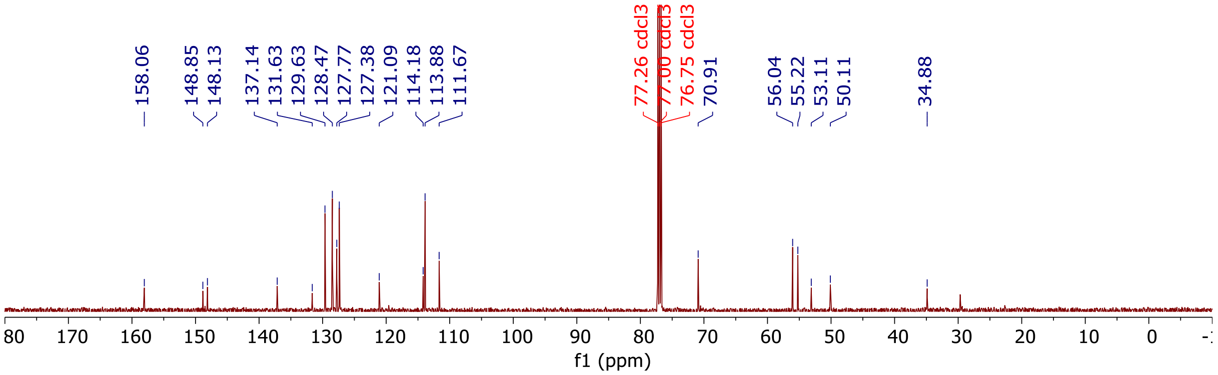

Figure S31. HRMS of *N*-allyl-*N*-(4-benzyloxy-3-methoxybenzyl)-2-(4-methoxyphenyl)ethan-1-amine (**13**)

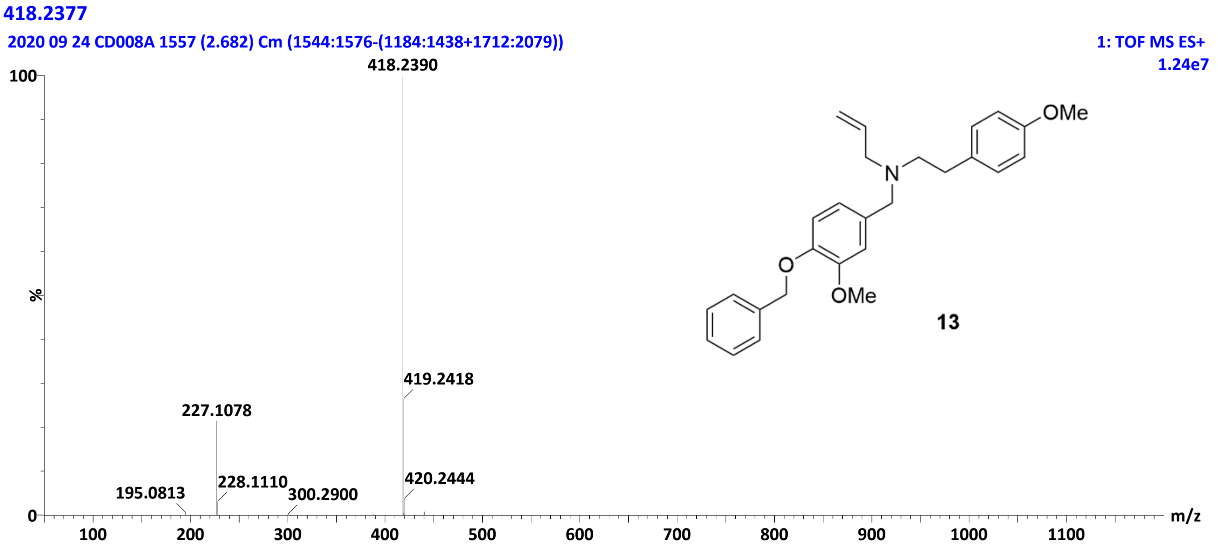

Figure S32. ^{1}H NMR spectrum of *N*-allyl-*N*-(4-benzyloxy-3-methoxybenzyl)-2-(4-methoxyphenyl)ethan-1-amine (13) in $CDCl_3$

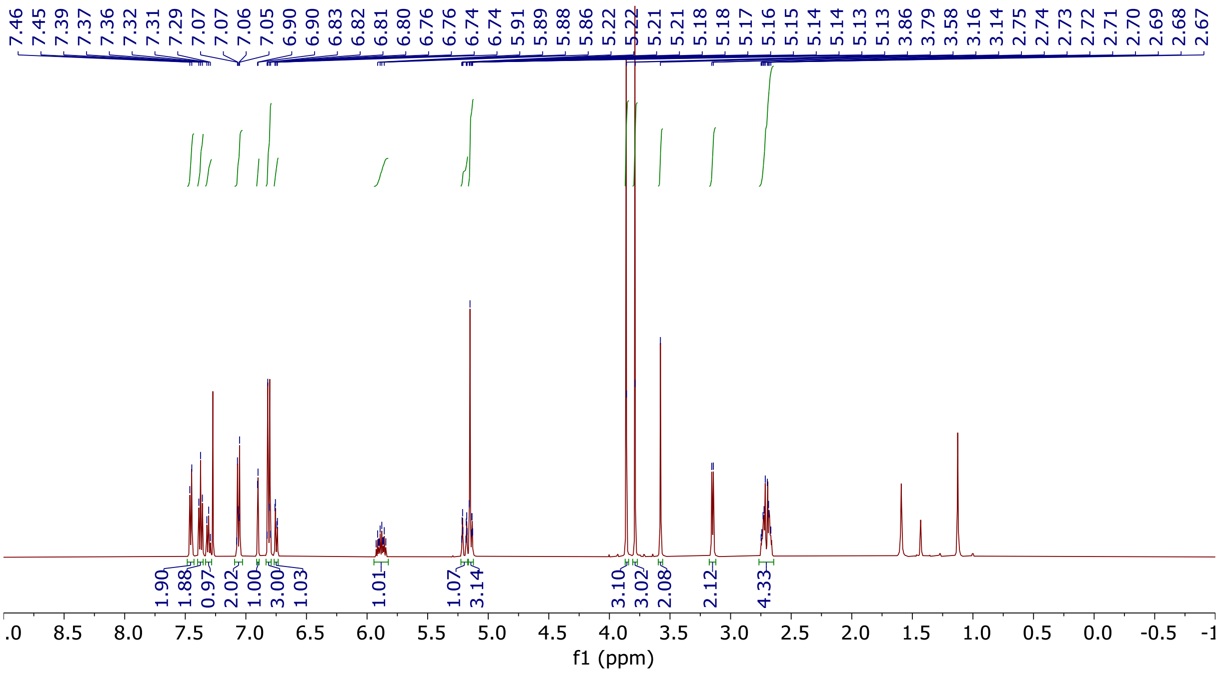

Figure S33. ^{13}C NMR spectrum of *N*-allyl-*N*-(4-benzyloxy-3-methoxybenzyl)-2-(4-methoxyphenyl)ethan-1-amine (13) in $CDCl_3$

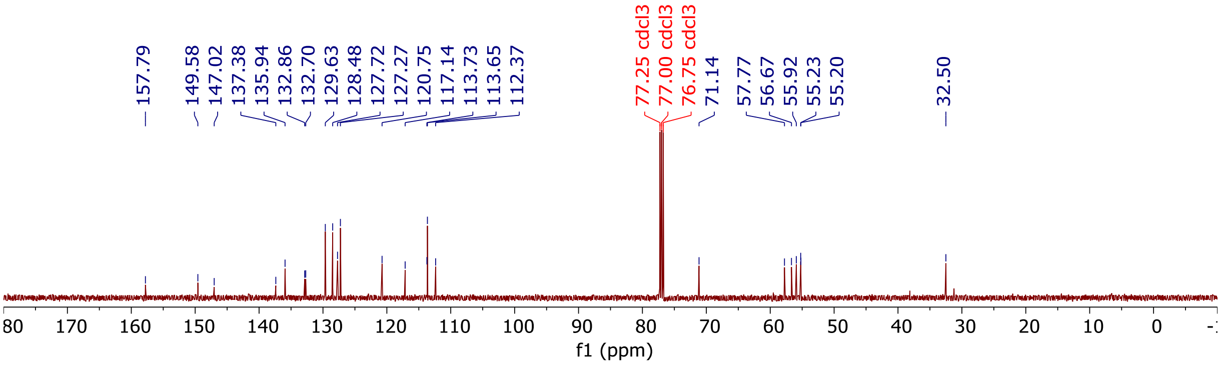

Figure S34. HRMS of *N*-allyl-*N*-(3-benzyloxy-4-methoxybenzyl)-2-(4-methoxyphenyl)ethan-1-amine (**14**)

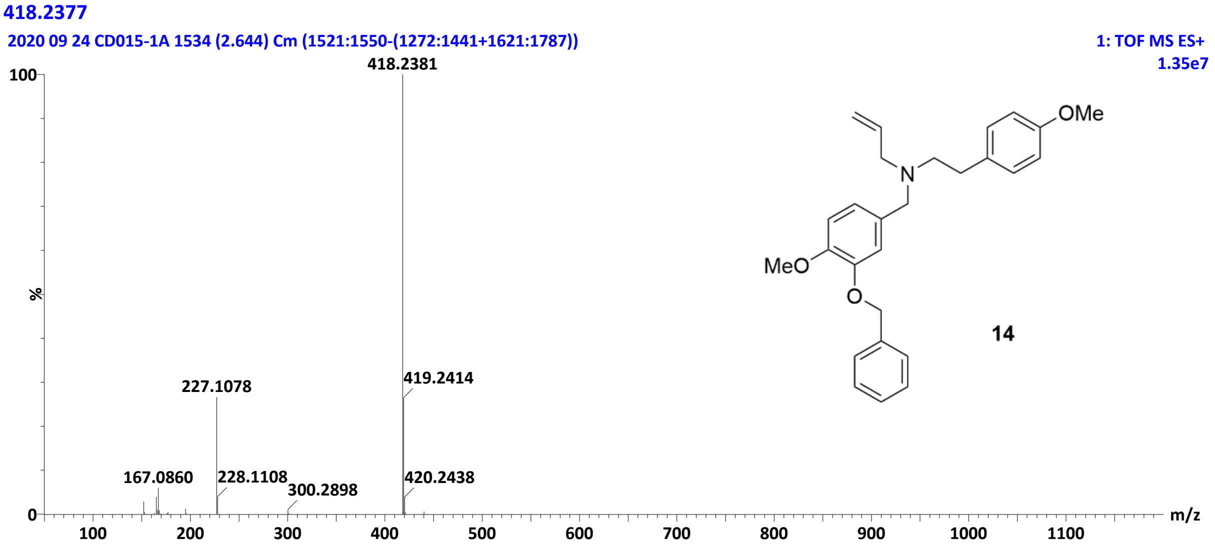

Figure S35. ^1H NMR spectrum of *N*-allyl-*N*-(3-benzyloxy-4-methoxybenzyl)-2-(4-methoxyphenyl)ethan-1-amine (14) in $CDCl_3$

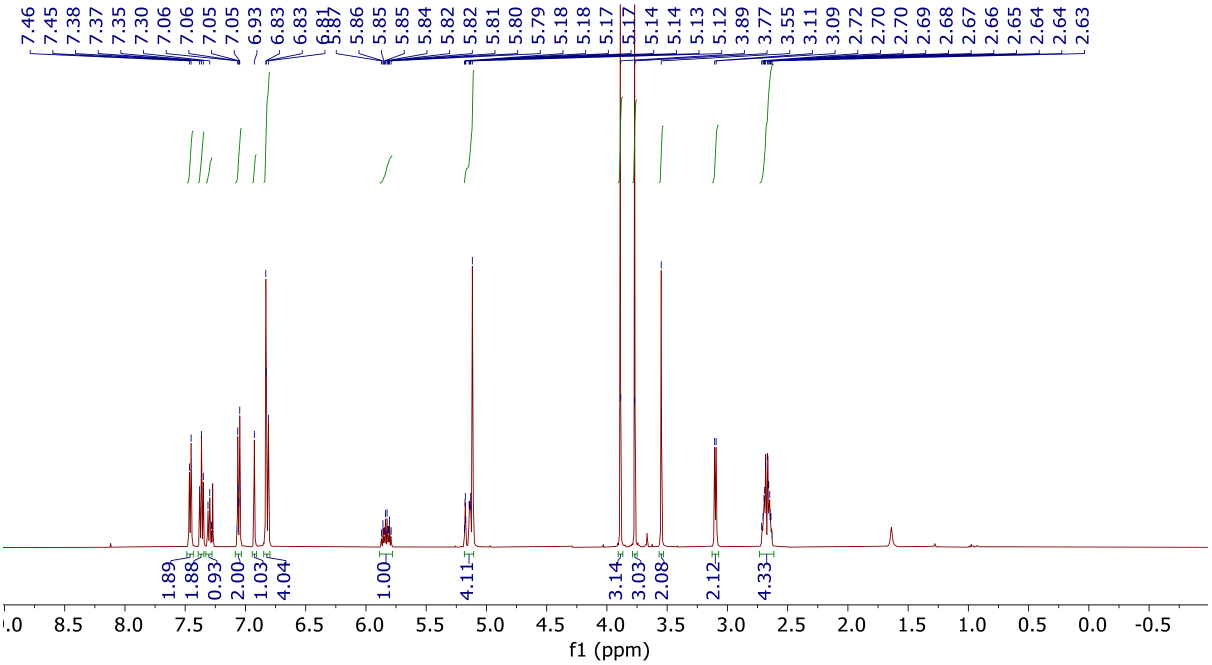

Figure S36. ^{13}C NMR spectrum of *N*-allyl-*N*-(3-benzyloxy-4-methoxybenzyl)-2-(4-methoxyphenyl)ethan-1-amine (14) in $CDCl_3$

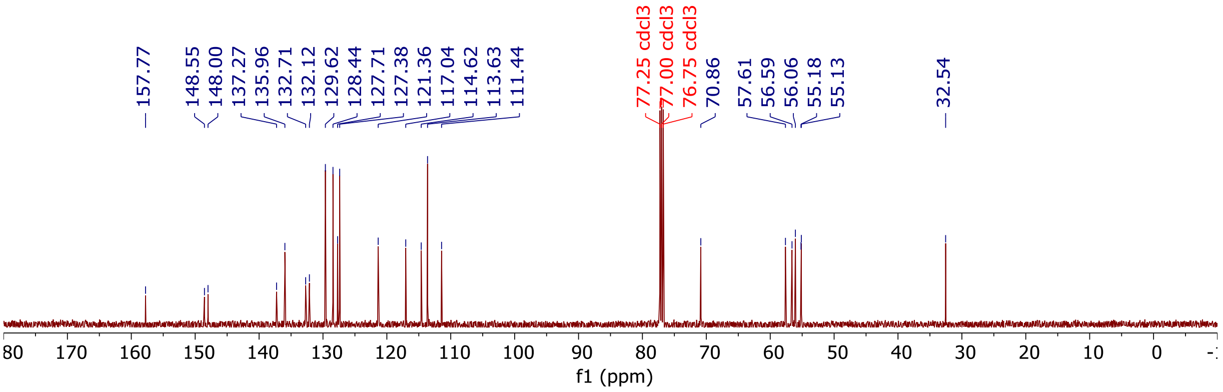

Figure S37. HRMS of *N*-(3-ethoxy-4-methoxybenzyl)-2-(4-hydroxyphenyl)ethan-1-amine (**15**)

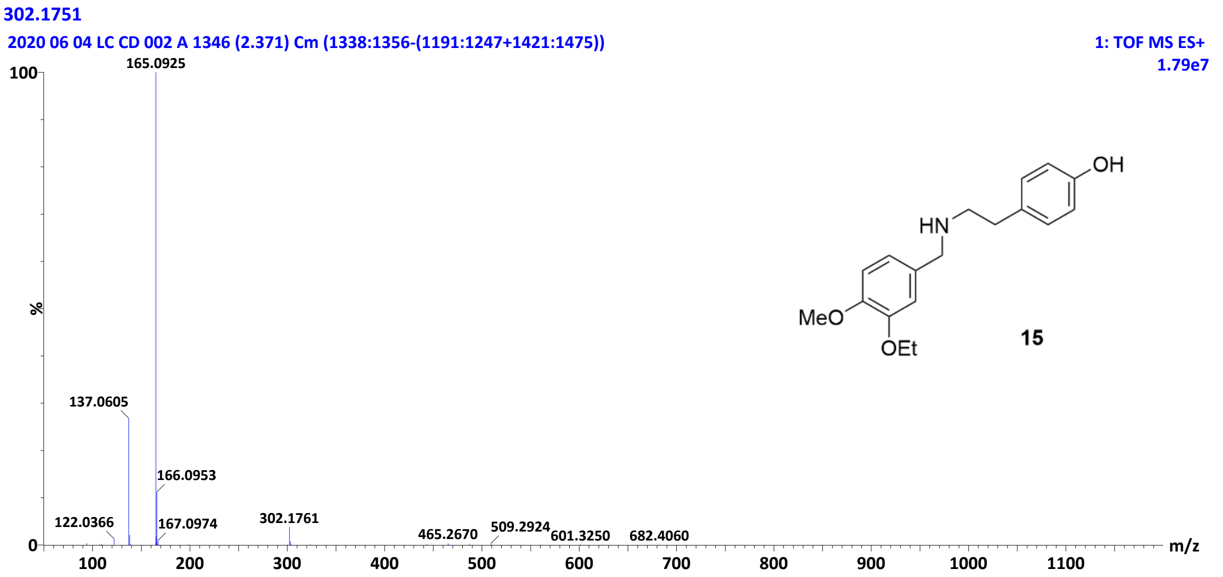

Figure S38. ^{1}H NMR spectrum of *N*-(3-ethoxy-4-methoxybenzyl)-2-(4-hydroxyphenyl)ethan-1-amine (15) in $CDCl_3$

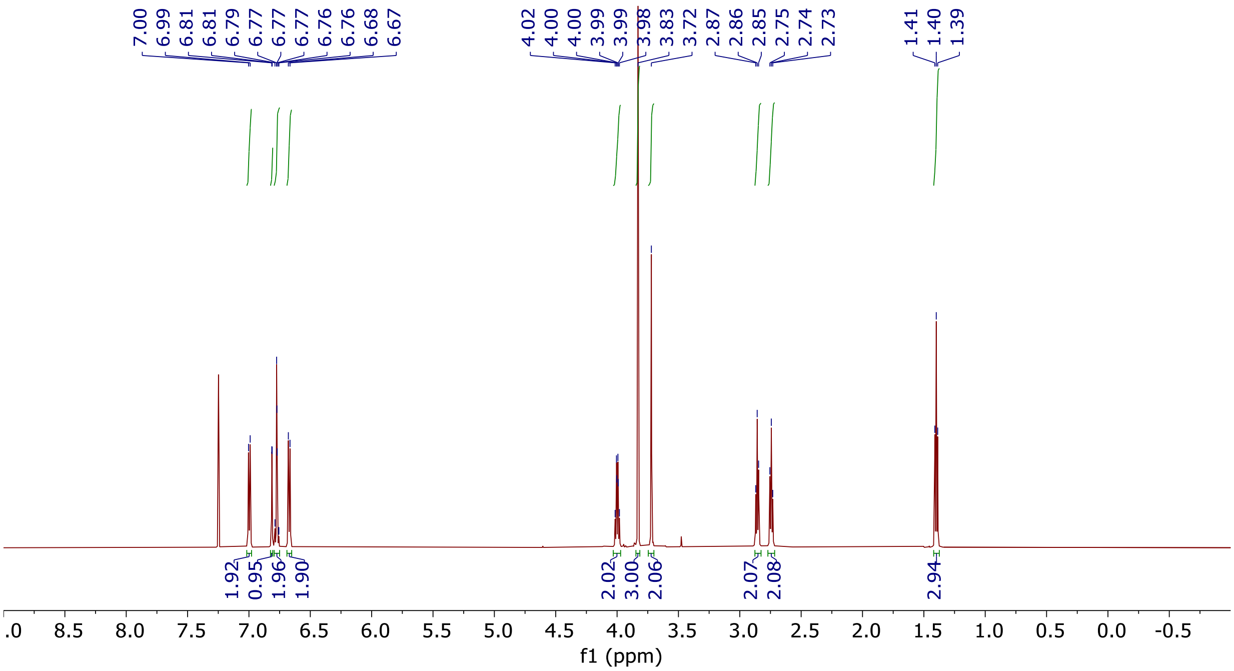

Figure S39. ^{13}C NMR spectrum of *N*-(3-ethoxy-4-methoxybenzyl)-2-(4-hydroxyphenyl)ethan-1-amine (15) in $CDCl_3$

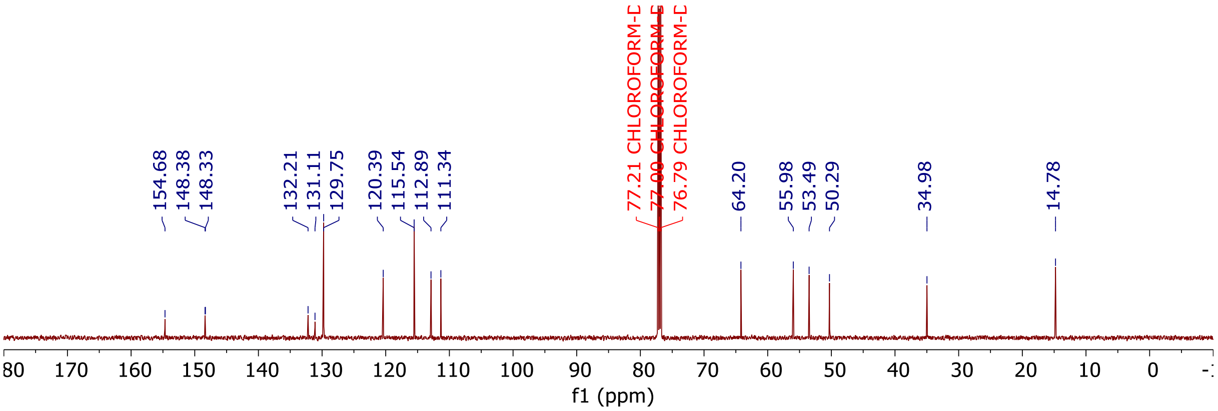

Figure S40. HRMS of *N*-allyl-*N*-(3-ethoxy-4-methoxybenzyl)-2-(4-hydroxyphenyl)ethan-1-amine (**16**)

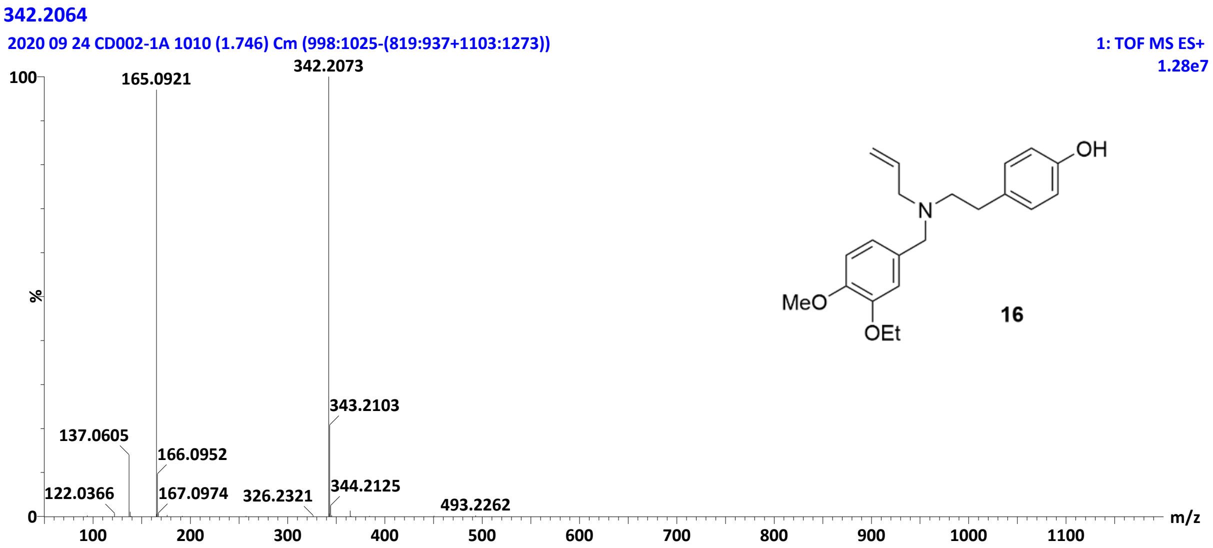

Figure S41. ^{1}H NMR spectrum of *N*-allyl-*N*-(3-ethoxy-4-methoxybenzyl)-2-(4-hydroxyphenyl)ethan-1-amine (16) in $CDCl_3$

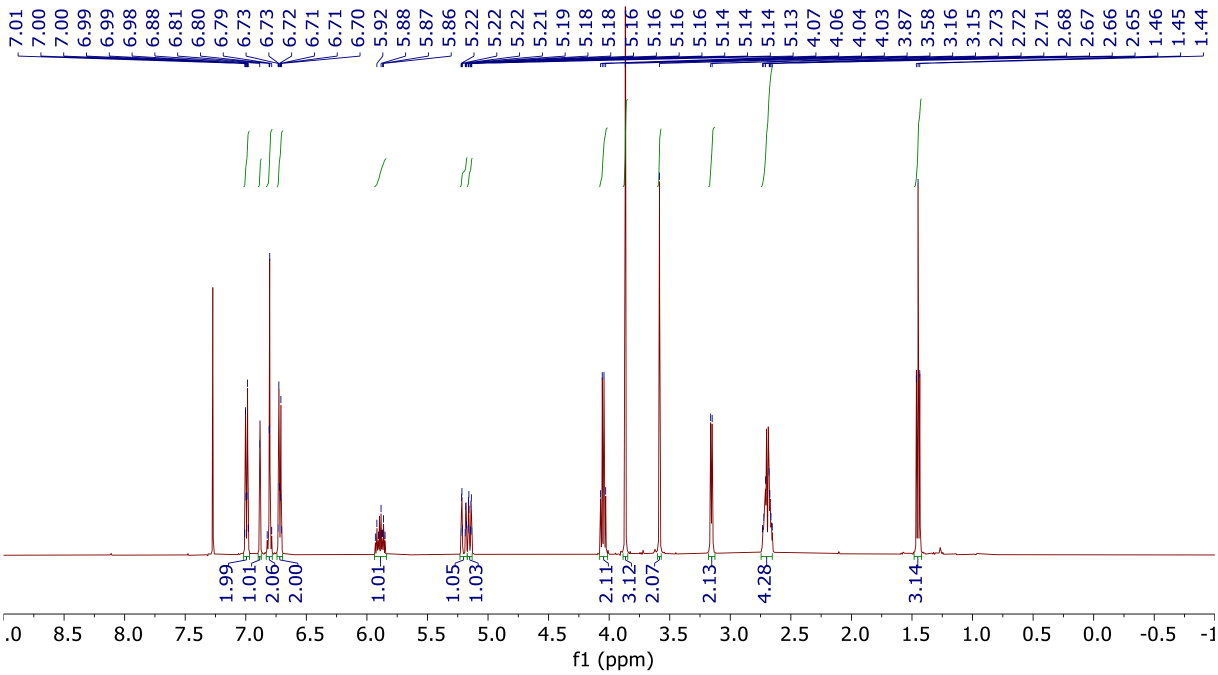

Figure S42. ^{13}C NMR spectrum of *N*-allyl-*N*-(3-ethoxy-4-methoxybenzyl)-2-(4-hydroxyphenyl)ethan-1-amine (16) in $CDCl_3$

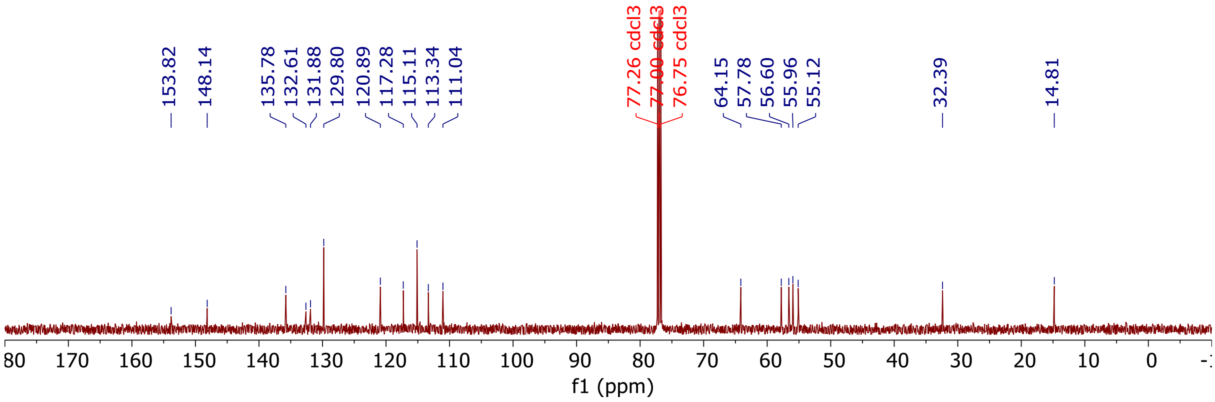

Figure S43. HRMS of *N*-allyl-*N*-(3-ethoxy-4-methoxybenzyl)-2-(4-allyloxyphenyl)ethan-1-amine (**17**)

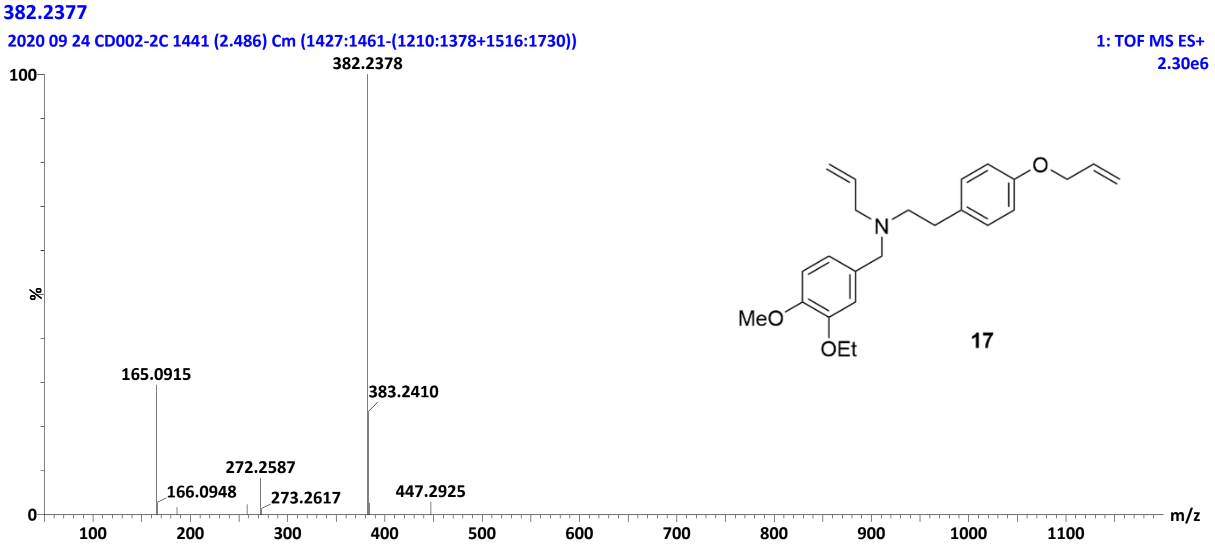

Figure S44. ^{1}H NMR spectrum of *N*-allyl-*N*-(3-ethoxy-4-methoxybenzyl)-2-(4-allyloxyphenyl)ethan-1-amine (17) in $CDCl_3$

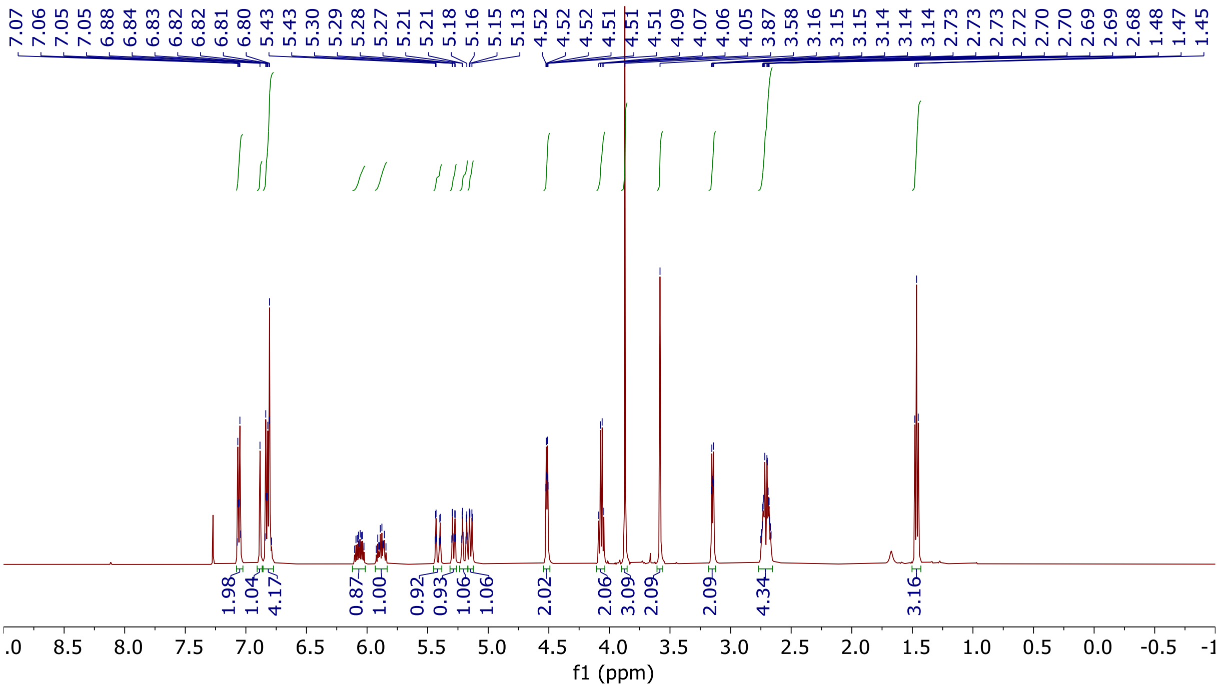

Figure S45. ^{13}C NMR spectrum of *N*-allyl-*N*-(3-ethoxy-4-methoxybenzyl)-2-(4-allyloxyphenyl)ethan-1-amine (17) in $CDCl_3$

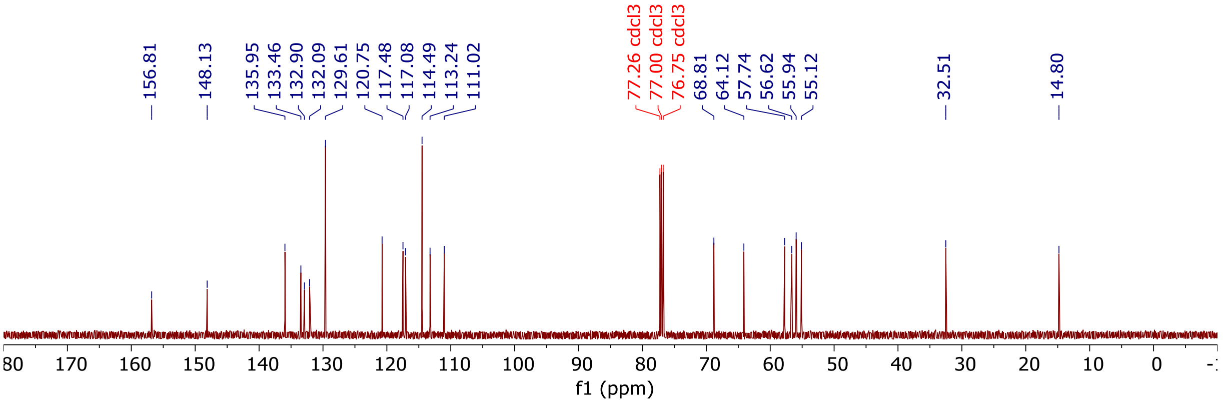

Figure S46. HRMS of *N*-(3-ethoxy-4-methoxybenzyl)-2-phenylethan-1-amine (**18**)

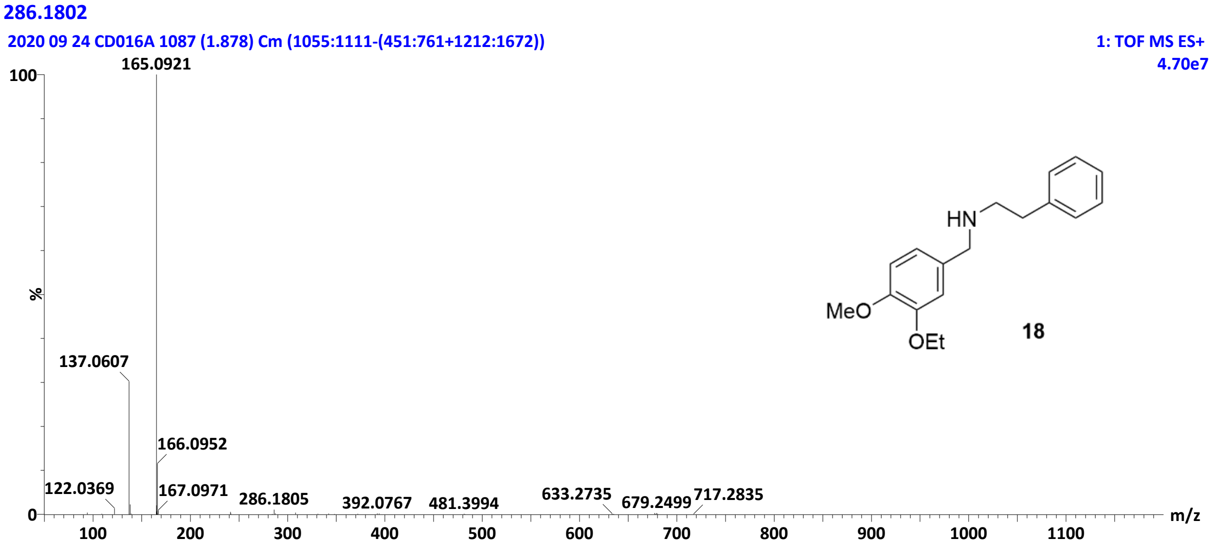

Figure S47. ^{1}H NMR spectrum of *N*-(3-ethoxy-4-methoxybenzyl)-2-phenylethan-1-amine (18) in $CDCl_3$

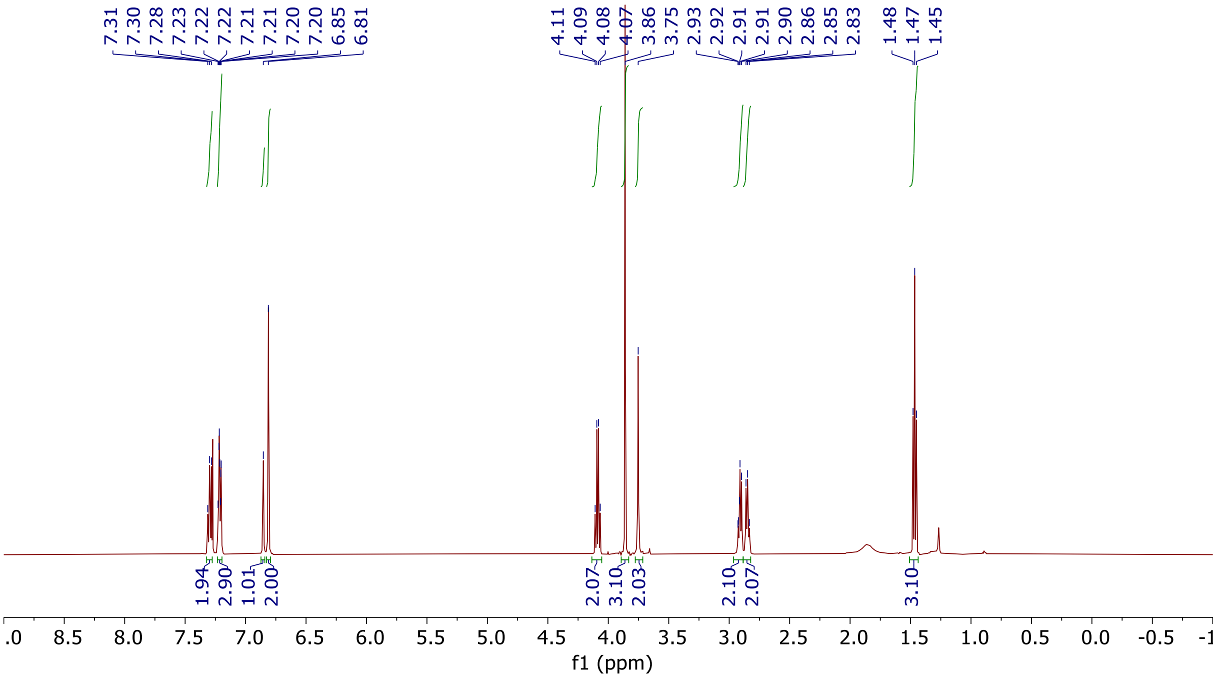

Figure S48. ^{13}C NMR spectrum of *N*-(3-ethoxy-4-methoxybenzyl)-2-phenylethan-1-amine (18) in $CDCl_3$

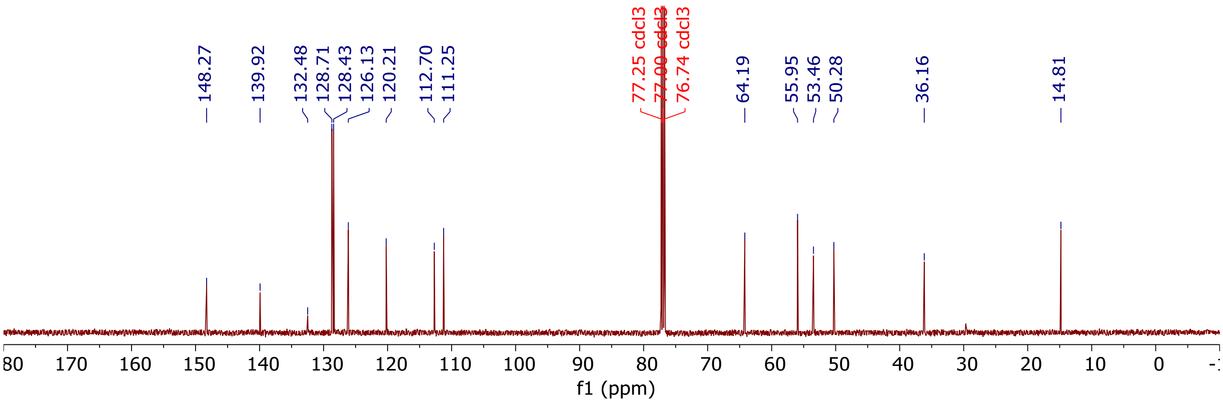

Figure S49. HRMS of *N*-allyl-*N*-(3-ethoxy-4-methoxybenzyl)-2-phenylethan-1-amine (**19**)

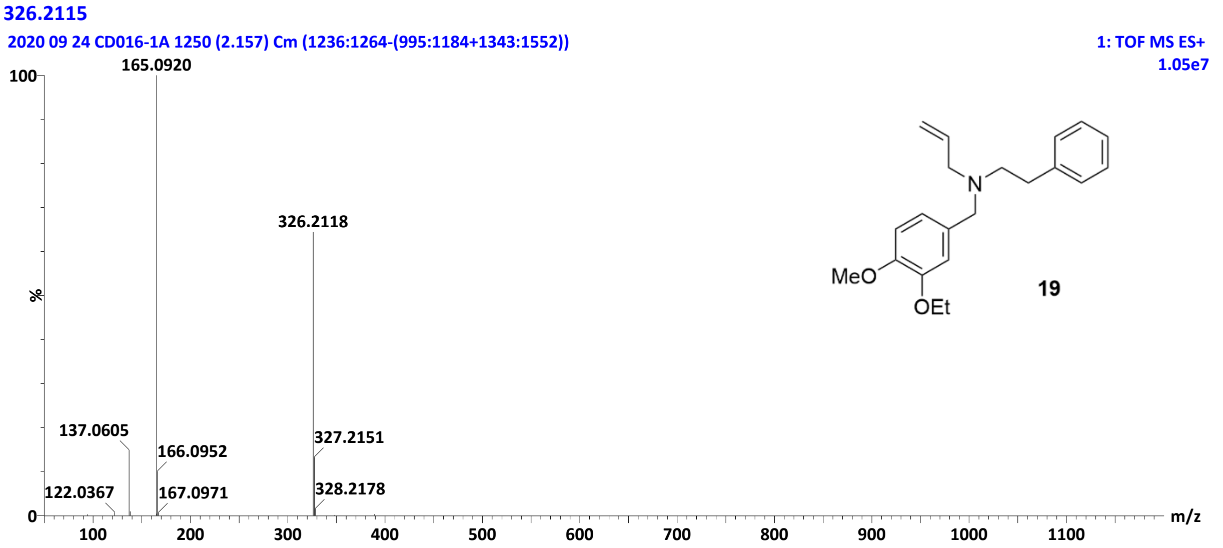

Figure S50. ^{1}H NMR spectrum of *N*-allyl-*N*-(3-ethoxy-4-methoxybenzyl)-2-phenylethan-1-amine (19) in $CDCl_3$

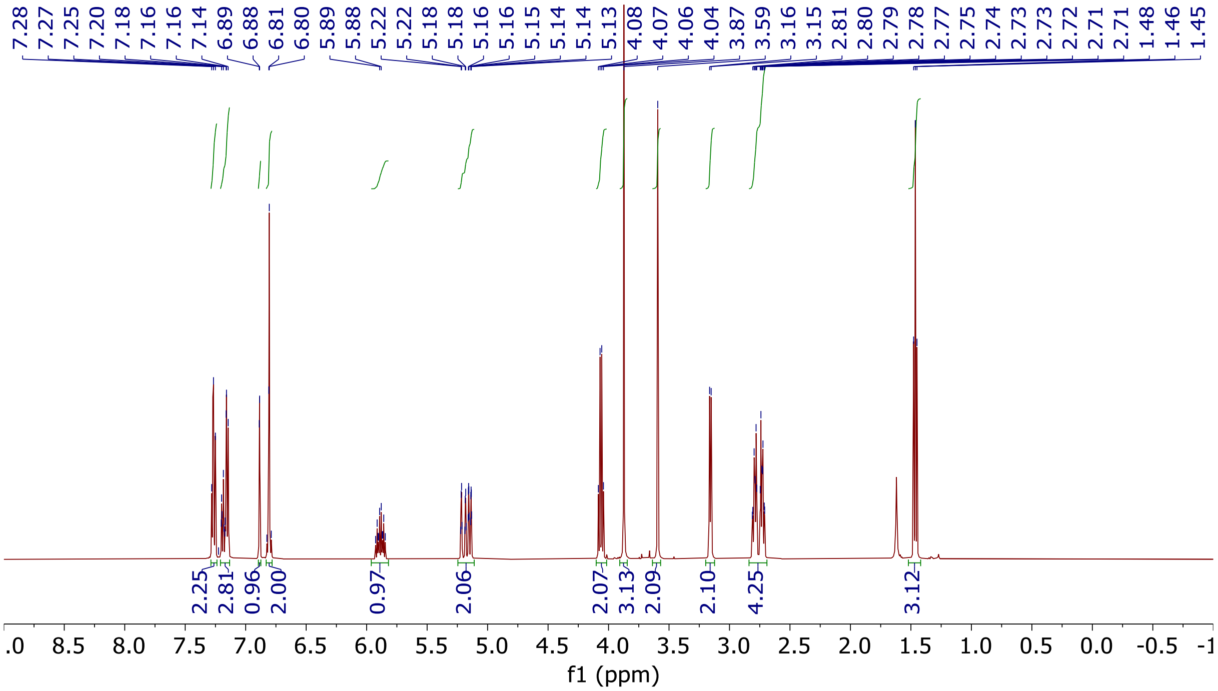

Figure S51. ^{13}C NMR spectrum of *N*-allyl-*N*-(3-ethoxy-4-methoxybenzyl)-2-phenylethan-1-amine (19) in $CDCl_3$

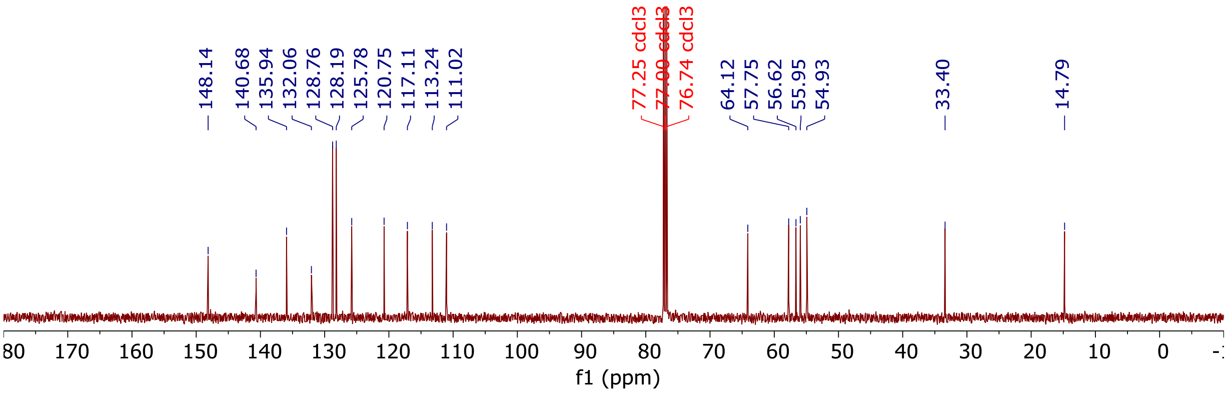

Figure S52. HRMS of *N*-benzyl-*N*-(3-ethoxy-4-methoxybenzyl)-2-phenylethan-1-amine (**2**0)

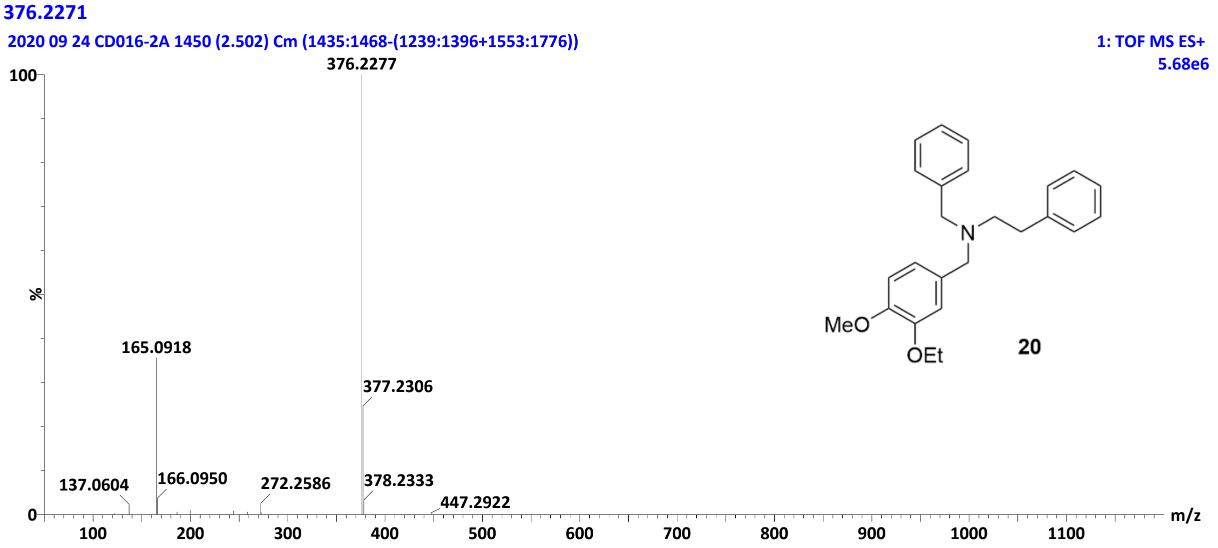

Figure S53. ^{1}H NMR spectrum of *N*-benzyl-*N*-(3-ethoxy-4-methoxybenzyl)-2-phenylethan-1-amine (20) in $CDCl_3$

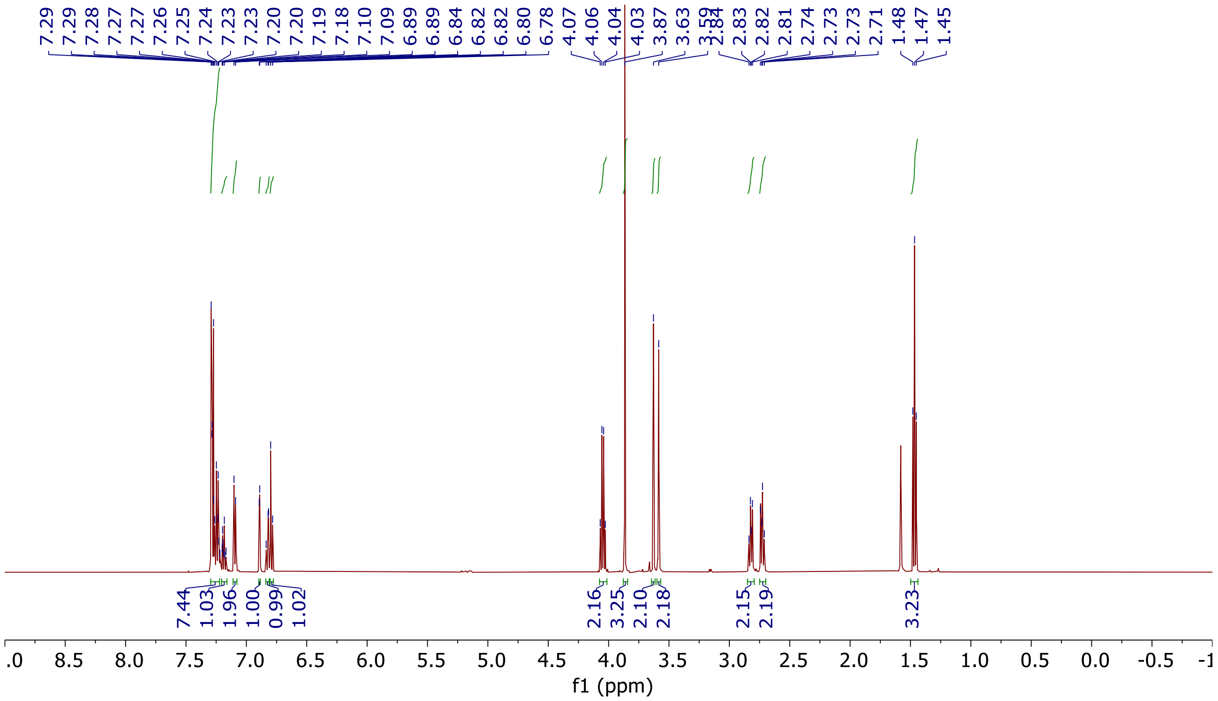

Figure S54. ^{13}C NMR spectrum of *N*-benzyl-*N*-(3-ethoxy-4-methoxybenzyl)-2-phenylethan-1-amine (20) in $CDCl_3$

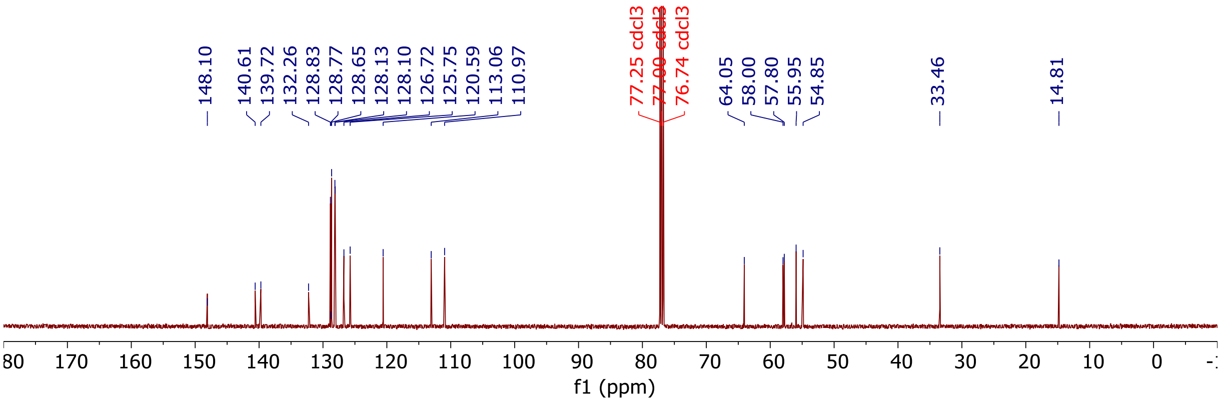

Biological assays in detail

hAChE and hBuChE Inhibition Assay

The inhibitory activities of prepared compounds and standards against human recombinant AChE (E.C. 3.1.1.7) and human plasma BChE (E.C. 3.1.1.8) were determined using modified Ellman's method [1-3] and expressed as IC_{50} (the concentration of the compound that is required to reduce 50% of cholinesterase activity). Human recombinant AChE, phosphate buffer (PB, pH = 7.4), 5,5'-dithio-bis(2-nitrobenzoic) acid (Ellman's reagent, DTNB), acetylthiocholine (ATCh), butyrylthiocholine (BTCh), and other used compounds were purchased from Sigma-Aldrich (Prague, Czech Republic). Human plasma was used as a source of BChE and was prepared from heparinized human blood. Blood was centrifuged for 20 minutes (4 °C, 2300 × g) by Hettich Universal 320R centrifuge. The plasma was separated and stored at -80 °C. During the measurement, 96-well microplates from polystyrene (ThermoFisher Scientific, Waltham, MA, USA) were used.

The solutions of she corresponding cholinesterase in PB were prepared up to the final activity 0.002 U/µL. The assay medium (100 µL) consisted of cholinesterase (10 µL), DTNB (20 µL of 0.01 M solution), and PB (40 µL of 0.1 M solution). The solutions of the tested compounds (10 µL of different concentrations) were pre-incubated for 5 minutes in the assay medium and then a solution of the substrate (20 µL of 0.01 M ATCh or BTCh iodide solution) was added to initiate the reaction. The increase of absorbance was measured at 412 nm using Multimode microplate reader Synergy 2 (BioTek Inc., Winooski, VT, USA). For the calculation of the resulting measured activity (the percentage of inhibition I) following formula was used:

$$I = \left(1 - \frac{\Delta A_i}{\Delta A_0}\right) \times 100$$

where ΔA_i indicates absorbance change provided by adequate enzyme exposed to corresponding inhibitor and ΔA_0 indicates absorbance change when a solution of PB was added instead of a solution of inhibitor. Software Microsoft Excel (Redmont, WA, USA) and GraphPad Prism version 6.07 for Windows (GraphPad Software, San Diego, CA, USA) were used for the statistical data evaluation.

Kinetic Study of Cholinesterase Inhibition

The kinetic study *h*BuChE was performed by using above mentioned modified Ellman's method. The values of V_{max} and K_m of the Michaelis-Menten kinetics as well as the values of K_i and $K_{i'}$ were calculated by nonlinear regression from the substrate velocity curves. Linear regression was used for the calculation of Lineweaver-Burk plots. All calculations were performed using GraphPad Prism software version 6.07 for Windows (San Diego, CA, USA).

POP inhibition assay

POP (EC 3.4.21.26) was dissolved in phosphate-buffered saline (PBS; 0.01 M Na/K phosphate buffer, pH 7.4, containing 137 mM NaCl and 2.7 mM KCl); the specific activity of the enzyme was 0.2 U/mL. The assay was performed in standard polystyrene 96-well microplates with a flat and clear bottom. Stock solutions of tested compounds were prepared in DMSO (10 mM). Dilutions (10^{-3} to 10^{-7} M) were prepared from the stock solution with deionized H_2O; the control was performed with the same DMSO concentration. POP substrate, (*Z*)-Gly-Pro-p-nitroanilide, was dissolved in 50% 1,4-dioxane (5 mM). For each reaction, PBS (170 µL), tested compound (5 µL), and POP (5 µL) were incubated for 5 min at 37 °C. Then, substrate (20 µL) was added, and the microplate was incubated for 30 min at 37 °C. The formation of p-nitroanilide, directly proportional to the POP activity, was measured spectrophotometrically at 405 nm using a microplate ELISA reader (Multimode microplate reader Synergy 2, BioTek Instruments, Winooski, VT). The inhibition potency of tested compounds was calculated by nonlinear regression analysis and was expressed as an IC_{50} value (concentration of inhibitor which causes 50% POP inhibition). All calculations were performed using GraphPad Prism software version 6.07 for Windows (GraphPad Software).

MAOs inhibition assay

The reaction mixture contains 2.5mg/mL MAO-A or 6.25mg/mLMAO-B enzyme (Merck, Germany) and inhibitor in final concentrations of 1, 5, 8, 10, 15, 30, 50, and 80mM in 50 mM potassiumphosphate buffer with 20% (v/v) glycerol (pH 7.5). The mixture was pre-incubated at 37 °C for 5 min and subsequently, substrate kynuramine was added to the final concentration of 60mM in the case of MAO-A and 30mM in the case of MAO-B. The final volume of the reaction mixture was 0.1 mL. The whole reaction mixture was incubated at 37 °C for 30 min. The reaction was stopped by the addition of 200mL acetonitrile:methanol mixture (1:1) and cooling down to 0 °C. The sample was then centrifuged (16.500 g) for10 min.The deamination product of kynuramine formed during the enzymatic reaction 4-hydroxyquinoline (4-HQ) was determined by UHPLC-MS on a Zorbax RRHD Eclipse plus C18 column (2.1 mm x 50 mm, 1.8mm) (Agilent Technologies, USA), by using a 6470 Series Triple Quadrupole mass spectrometer (Agilent Technologies, USA) as detector with electrospray ionisation – positive ion mode. Three m/zMRM transitions were followed for kynuramine (165.1=>30.2, 165.1=>118.0, 165.1=>136.0) and 4-HQ (146.1=>51.1, 146.1=>77.0, 146.1=>91.0). Eluents: (A) 0.1% formic acid in water; (B) 0.1% formic acid in acetonitrile. $IC_{50}$of individual compounds were determined by nonlinear regression usingGraphPad Prism 8.0 (GraphPad Software, USA).
